# Crystalline Half-Parent Vinyl-tetrylenes: Synthesis and Coordination Chemistry

**DOI:** 10.1021/acs.inorgchem.5c04745

**Published:** 2025-12-05

**Authors:** Annika Schulz, Terrance J. Hadlington

**Affiliations:** Fakultät für Chemie, School of Natural Sciences, TU München, Lichtenberg Strasse 4, 85749 Garching, Germany

## Abstract

A family of stable, crystalline half-parent vinyl-tetrylenes ^R^L­(C_2_H_3_)­E: (**4**-**6**; ^R^L = {[R_2_PCH_2_Si­(^i^Pr)_2_]­(Dipp)­N}^−^; Dipp = 2,6-^i^Pr_2_C_6_H_3_; R = Ph, Cy) is reported, accessed via a straightforward salt-metathesis pathway, and their electronic properties and initial reactivity explored. All compounds display downfield-shifted signals in both the ^1^H and ^13^C NMR spectra for their [C_2_H_3_] ligand, being most extreme for Pb due to relativistic effects. UV–vis spectra of compounds **4**–**6** reveal bathochromic shifts relative to the halo-tetrylene derivatives (i.e., ^R^LEX; X = Cl, Br), due to stabilization of the LUMO through overlap of the E-centered *p*-orbital and the π*-orbital of the [C_2_H_3_] unit. Vinyl group elimination is demonstrated in the plumbylene system, whereby reaction with the strong electrophile [Ph_3_C]­[BAr^F^_4_] (Ar^F^ = 3,5–(CF_3_)_2_C_6_H_3_) afforded low-coordinate Pb^II^ cation **7**, stabilized by the chelating ligand and secondary interactions with the weakly coordinating BAr^F^_4_ anion. Finally, addition of vinyl-germylenes (**4**) to Ni^0^ synthons enabled the synthesis of mono- and bis­(vinylgermylene)-nickel(0) complexes **8** and **9**, whereby binding occurs through the tetryl centers and not the vinyl fragment, representing the first examples of vinyl-tetrylene coordination complexes reported to date.

## Graphical abstract

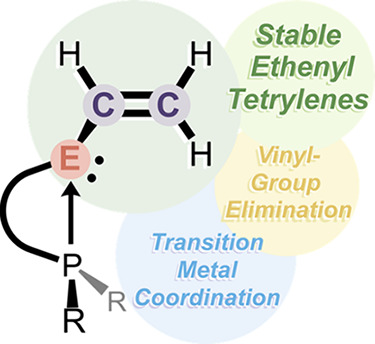

## Introduction

Recent years have demonstrated a remarkable reactivity of low-valent main group compounds akin to transition-metal mediated transformations, including small molecule activation, insertion processes, and even reversible bond scission.^1,2^ Such reactivity is particularly attractive in the search for alternatives to scarce and potentially toxic transition metals in catalysis.^3,4^ Among these systems, tetrylenes stand out for their ability to cleave strong bonds in numerous substrates (e.g., H_2_,^5–7^ NH_3_,^8–10^ C_6_H_6_,^11^ C_6_H_5_X,^12–14^ (X = Hal)).^2^ Their ambiphilic electronic structure, combining a lone electron pair and a vacant *p*-orbital, drives this reactivity, and additionally makes them valuable in broader coordination chemistry.^15^ Accordingly, tetrylenes and their role as ligands in transition metal complexes has also been intensively explored in recent years.^16,17^ Since heavier tetrylenes predominantly adopt a singlet ground state,^18,19^ their reactivity is largely governed by the energy gap between the occupied high *s*-character orbital (Highest Occupied Molecular Orbital; HOMO) and the vacant *p*-orbital (Lowest Unoccupied Molecular Orbital; LUMO), i.e. Δ*E*_HOMO/LUMO_. This energy gap generally increases on descending group 14.^18,20^ Nevertheless, this can be more specifically tuned by modifying the chemical environment surrounding the tetrylene center, e.g. by adapting the steric bulk, and σ- and π-donating/accepting properties of the ligand at the tetryl element center.^21^

Electron-withdrawing substituents stabilize the tetrylene center via the inductive (−I) effect by lowering the energy of the nonbonding σ-orbital. This results in an increased Δ*E*_HOMO/LUMO_.^22,23^ Alternatively, the divalent group 14 center can be stabilized via the mesomeric (+M) effect by in-plane donation of a lone electron pair to the vacant *p*-orbital of the tetrylene.^21,24^ A prime example here is the class of diaminocarbenes, which have garnered significant importance in modern chemistry following their landmark discovery by Arduengo and co-workers in 1991.^25,26^ Combining inductive and mesomeric concepts generates a ‘push–pull’ effect: donation of two nitrogen lone pairs into the empty *p*-orbital of the carbene center, combined with inductive effects arising from the electronegative nitrogen atoms, leads to highly stable carbene ligands.^23^ Conjugated π-systems, e.g. aryl, allyl, and vinyl substituents, allow for the delocalization of electrons and therefore can greatly influence the electronic properties of a tetrylene center. In this line, Power and co-workers demonstrated that bis­(aryl)-germylene and -stannylene complexes can readily cleave dihydrogen and ammonia, leading to tetravalent germanium­(IV) species but divalent tin­(II) complexes,^5,27^ reactivity which has not yet been demonstrated for e.g. bis­(amido)­tetrylenes.^28,29^

In a pioneering study, Apeloig and co-workers investigated the influence of ethynyl and ethenyl substituents on the frontier orbital energies of silylene species.^30^ These theoretical studies examined the electronic absorption spectra of the compounds, demonstrating that vinyl substituents cause a bathochromic shift of the absorption due to a decrease in Δ*E*_HOMO/LUMO_. This is attributed to the electronic effects of the unsaturated ligand, as the excited state is significantly stabilized by an efficient *p*(Si)−π*­(vinyl) interaction, that is a delocalization of the vacant *p*-orbital at Si across the vinyl ligand. Despite this theoretical contribution to the study of vinyl-substituted tetrylenes, no ‘bottleable’ tetrylenes bearing the parent vinyl ligand, [C_2_H_3_], have been experimentally realized to date. A number of substituted vinyl tetrylenes, however, are known, bearing various fragments at the α– or β–carbon centers. These have been accessed through alkyne insertion into E-X bonds (Figure 1(a); E = Ge, Sn, Pb; X = H,^31–34^ C,^35^ B,^36^ Si^37^), [2 + 2] cycloaddition of alkynes to E^I^ dimers (Figure 1(b)),^38,39^ or in the specific case of bulky N-heterocyclic vinyl ligands, through salt-metathesis (Figure 1(c)),^40–42^ whereby bis­(vinyl)­tetrylenes were accessed. For those latter species, Density Functional Theoretical (DFT) calculations were indicative of significant delocalization of the C = C π-units across the E^II^ center’s *p*-orbital, while this was not observed for the π*-orbitals likely due to the presence of N-heterocyclic substituents at the β-carbon centers.^41^

**Figure 1:**
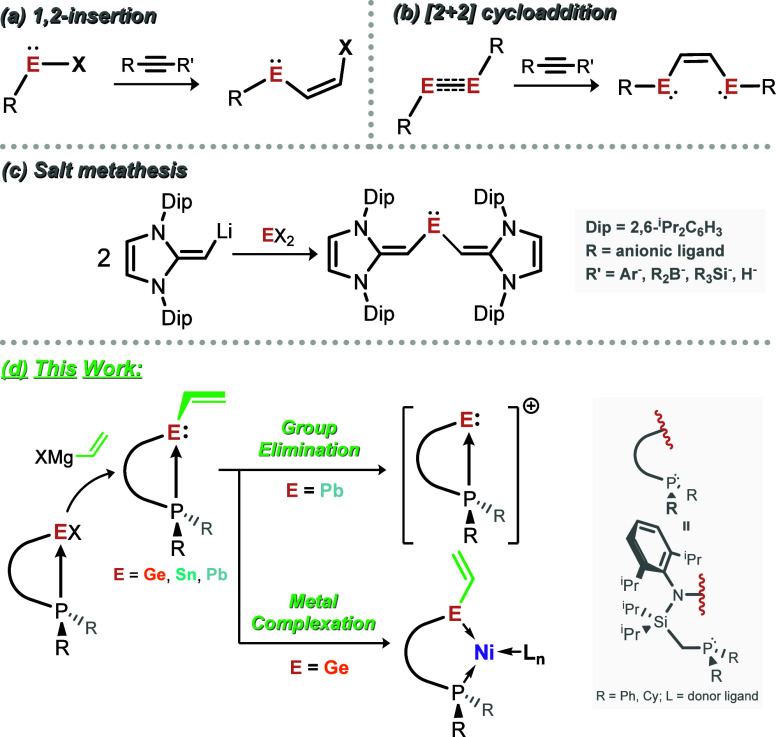
(a–c) Known pathways to vinyl-tetrylene compounds, and (d) this work, demonstrating the first syntheses of stable ethenyl-tetrylenes, and their reactivity in vinyl-group elimination, and Ni^0^ complexation.

Our own work has sought to understand the electronic effects of tetrylene ligand modifications on their bonding with transition metals. This makes the incorporation of conjugated systems into the ligand framework particularly attractive, as Apeloig has shown that such substituents can significantly influence the electronic properties of the tetrylene center.^30^ In addition, the small [C_2_H_3_] ligand may open additional opportunities for further functionalization at the tetrylene center, e.g. in group elimination. As mentioned, to date, no free tetrylenes featuring the [C_2_H_3_] ligand have been reported, and, while closely related systems (e.g., metallacyclobutenes, heavier vinylidene complexes) are known,^43–46^ no transition metal complexes which feature any vinyl-tetrylene ligand have been reported. Herein, we describe initial examples of half-parent vinyl-tetrylenes for Ge, Sn, and Pb, accessed via simple salt-metathesis with commercially available Grignard reagents. Vinyl-group elimination and transition metal coordination are described, giving the first insights into the reactive character of this class of tetrylene system, with key electronic insights given through DFT calculations.

## Results and Discussion

### Synthesis and Characterization of Half-Parent Ethenyl-Tetrylenes

In recent years, our group has reported a variety of chloro-tetrylene species which feature phosphine-appended amido ligands, ^R^L (^R^L = {[R_2_PCH_2_Si­(^i^Pr)_2_]­(Dipp)­N}^−^; Dipp = 2,6-^i^Pr_2_C_6_H_3_; R = Ph, Cy). These have been utilized as scaffolds to support a variety of neutral and cationic germylene- or stannylene-ligated transition metal complexes, whereby the phosphine arm greatly aids in complex stabilization.^47–50^ In the context of this current study, we have expanded this scope to halo-plumbylenes, and sought to explore the utility of this family of halo-tetrylenes as precursors to access as yet unknown ethenyl-functionalized tetrylenes. The addition of 1 M THF solutions of a commercial vinyl Grignard reagent, BrMg­(C_2_H_3_), to the respective colorless ^R^L­(X)­E: (X = Cl or Br; R = Ph, E = Ge (**1a**), Sn (**2a**), Pb (**3a**); R = Cy, E = Ge (**1b**), Sn (**2b**), Pb (**3b**)) solution in THF led to a gradual color change to dark yellow (Scheme 1), with reaction times of between 3 and 24 h. In situ ^31^P­{^1^H} revealed the formation of a single new species in all cases (e.g., for **4a**: δ = 1.34 ppm; for **5a**: δ = – 6.30 ppm, ^1^ *J*_117SnP_ = 609 Hz, ^1^ *J*_119SnP_ = 637 Hz; for **6a**: δ = 7.89 ppm, ^1^ *J*_207PbP_ = 898 Hz), attributed to the successful synthesis of vinyl tetrylenes **4** (E = Ge), **5** (E = Sn), and **6** (E = Pb). Following extraction with toluene and trituration with pentane, off-white powders were yielded in all cases, in reasonable to good yields (e.g., **4a**: 72%; **5a**: 65%; **6a:** 54%).

**Scheme 1:**
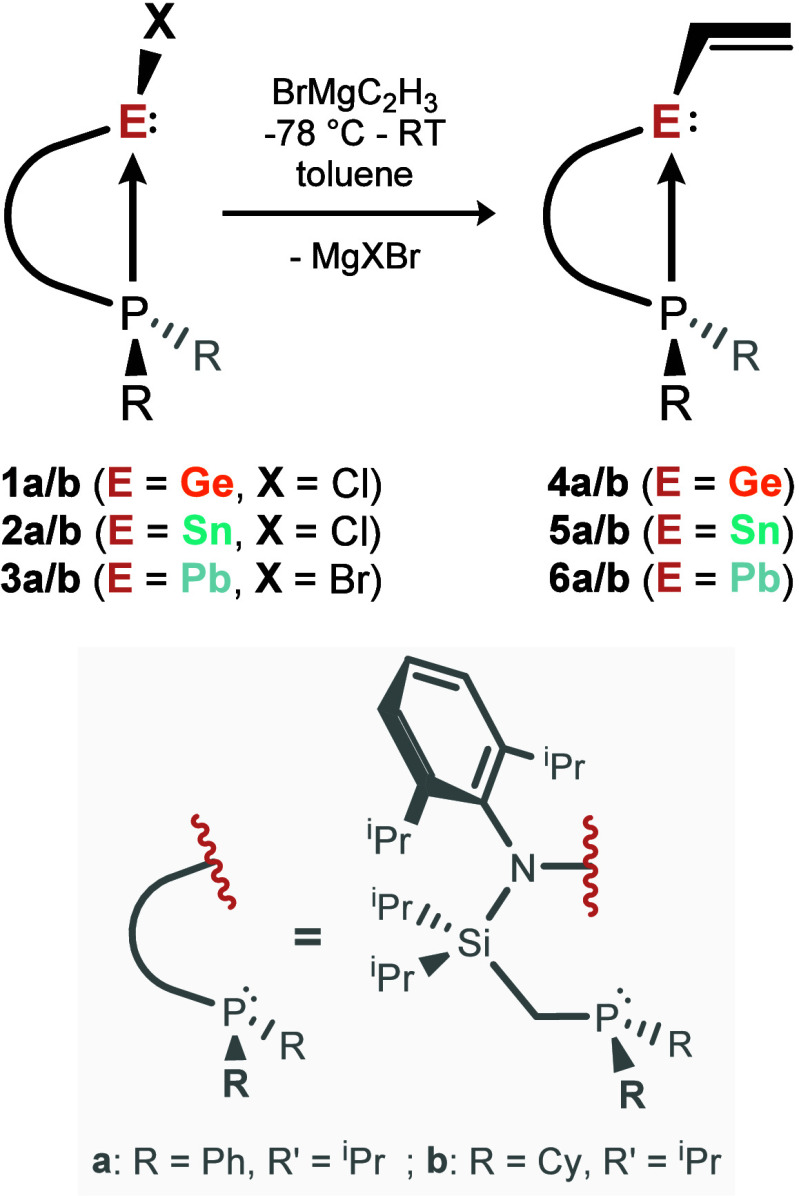
Synthesis of Vinyl-tetrylenes **4**-**6** via the Corresponding Halides and Vinylmagnesium Bromide

Single crystal X-ray analyses of **4a**, **5b**, and **6b** reveal that, in each case, the three-coordinate E^II^ centers are bonded to the chelating ligand system, via the amido and phosphine moieties, in addition to the vinyl-substituent, which is coordinated in a η^1^-fashion (Figure 2). Asymmetric units for **4a** and **5b** contain two independent molecules of those species (see Figures S62 and S63 in ESI), of which one shows the E-center being essentially coplanar with the alkenyl C = C residue and the nitrogen (torsion_NECC_ = 165.5(1)° (Ge) and 164.4(7)° (Sn)), which presumably allows for overlap of the *p*-orbital at E, which lies perpendicular to this plane. For the Pb species **6b** only this planarity is seen, perhaps highlighting the increased Lewis acidy of Pb^II^ vs Ge^II^ and Sn^II^. On moving from Ge to Pb each species exhibits a N–E–C_vinyl_ angle more closely approaching right angles (**4a**: 102.0(3)°; **5b**: 99.9(2)°; **6b**: 93.9(2)°), due to decreased *sp*-mixing on descending the group. Comparing the bond lengths in **4a** to the respective chloro-germylene, a significant elongation of the Ge–P bond is observed (^Ph^L­(Cl)­Ge: 2.3404(9) Å; **4a**: 2.483(2) Å) as well as the Ge–N bond (^Ph^L­(Cl)­Ge: 1.925(2) Å, **4a**: 1.959(7) Å). This may be attributed to the π-donor properties of the introduced vinyl-group, filling the *p*-orbital at Ge, in addition to the delocalization of the π-symmetry acceptor orbital at Ge over the vinyl unit. The E–C_vinyl_ bond lengths in **4a** (2.020(9) Å), **5b** (2.203(9) Å), and **6b** (2.316(7) Å) fall within the range of literature-reported σ-bound vinyl-tetrylenes, and related compounds featuring E^II^–C single bonds.^31,35,40–42^ With this, single-bond character is implied, negating any multiple-bond character between E and the vinyl-substituent. In accordance, the vinylic C = C bond lengths in each vinyl-tetrylene align with the expected average length of a C = C double bond of around 1.32 Å (i.e., d_C=C_: **4a** = 1.27(1) Å; **5a** = 1.32(1) Å; **6b** = 1.32(1) Å).[Fn fn1]

**Figure 2:**
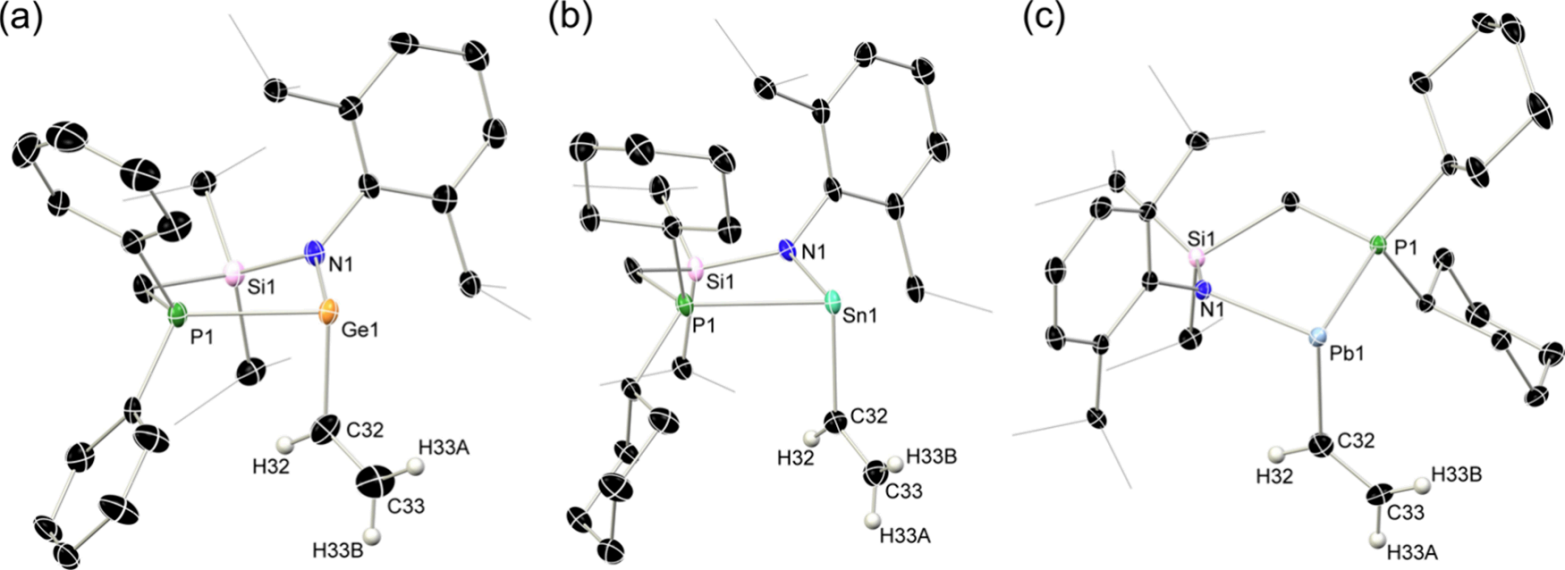
Molecular structures in the solid state of **4a**, **5b**, and **6b** with thermal ellipsoids at 30% probability, and hydrogen atoms omitted for clarity. Selected bond distances (Å) and angles (°) for **4a**: Ge1–C32 2.020(9); C32–C33 1.27(1); Ge1–N1 1.959(7); Ge1–P1 2.483(2); P1–Ge1–C32 88.3(3); N1–Ge1–C32 102.0(3). For **5b**: Sn1–C32 2.203(9); C32–C33 1.316(1); Sn1–N1 2.161(5); Sn1–P1 2.697(2); P1–Sn1–C32 94.5(1); N1–Sn1–C32 99.9(2). For **6b**: Pb1–C32 2.316(7); C32–C33 1.317(8); Pb1–N1 2.313(3); Pb1–P1 2.829(2); P1–Pb1–C32 89.8(2); N1–Pb1–C32 93.9(2).

Multinuclear NMR experiments give further insights into the electronic nature of **4**-**6**. ^1^H NMR spectra exhibit characteristic splitting patterns for all vinylic protons (Figure 3). In **6a**, proton **H** **_a_** appears as a doublet of doublet of doublets (ddd), exhibiting H–H_trans_ (^3^ *J*_H–H_ = 20.8 Hz), H–H_cis_ (^3^ *J*_H–H_ = 13.2 Hz) and weak H–P (^3^ *J*_H–P_ = 1.5 Hz) coupling. In contrast, **H** **_b_** only shows coupling to adjacent protons, H–H_cis_ (^3^ *J*_H–H_ = 13.2 Hz) and H–H_vic_ (^2^ *J*_H–H_ = 2.8 Hz), appearing as a doublet of doublets, i.e. without coupling to the ^31^P nucleus. **H** **_c_** also appears as a ddd, despite only having two neighboring protons; these involve a H–H_trans_ (^3^ *J*_H–H_ = 20.8 Hz), H–H_vic_ (^2^ *J*_H–H_ = 2.8 Hz), and a weak H–P coupling (^4^ *J*_H–P_ = 1.8 Hz). Notably, signals relating to **H** **_a_**, bound at the α-carbon, are shifted significantly downfield, in both the ^1^H and ^13^C spectra, with the greatest shifts being for Pb compounds **6** (e.g., **6a**; δ_1H_ = 10.52 ppm; δ_13C_ = 231.5 ppm). This pronounced deshielding arises from a combination of electronic and relativistic effects; one well-established concept is heavy atom on light atom (HALA) shielding, which originates from spin–orbit coupling and scalar effects.^51,52^ This was found to be especially pronounced in Pb^II^ units, to a smaller extent for Sn^II^, and in heavier chalcogenide species.^53^ This leads to remarkably deshielded proton and carbon signals in the respective NMR spectra.^54^ We hypothesize that this, in conjunction with the π-acceptor nature of low-valent tetrylenes, which may overlap with the vinyl ligand π-system, leads to these extreme chemical shifts. Notably, known Pb^II^-*alkyl* systems do not demonstrate an abnormal shift for either terminal-C or α-H centers, while closely related Pb^II^ (e.g., aryl) systems do.^31,55^

**Figure 3:**
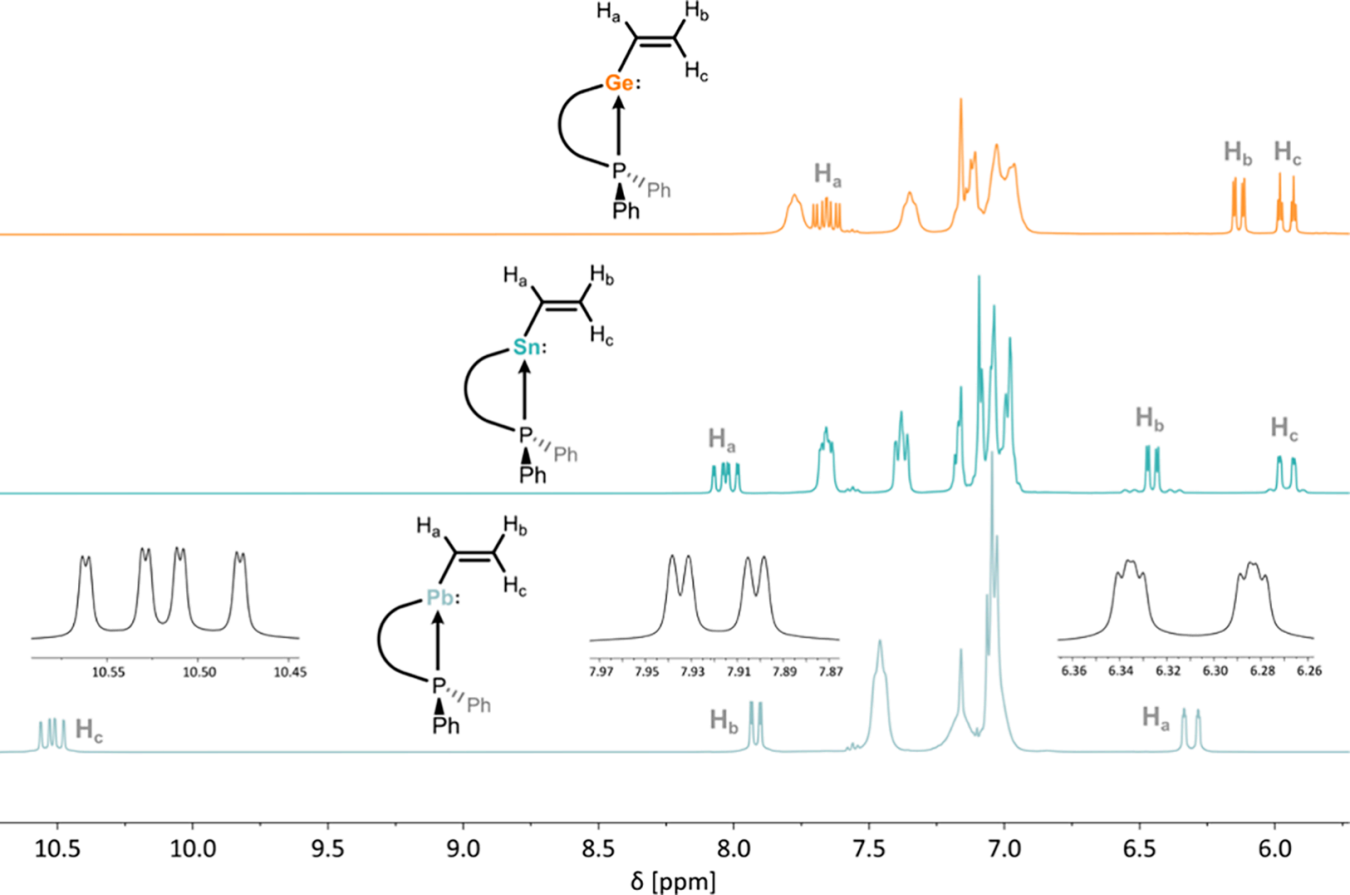
Excerpt of the ^1^H NMR spectra of **4a**, **5a**, and **6a** comparing the relative shifts of the vinylic protons. Zoomed portions are included for **6a**.

In the earlier discussed theoretical study from Apeloig et al., it was demonstrated that a bathochromic shift is found for vinyl-silylene relative to the hydrogen-substituted analogue, showing that the red-shifted absorption maximum is the result of mixing of the π*-orbital of the unsaturated alkenyl with the vacant *p*-orbital at Si^II^.^30^ To further elucidate related electronic effects in **4**-**6**, UV–vis spectra of **4a**, **5a** and **6a** were collected (Figure 4), alongside data for ^Ph^L­(Cl)­Ge: (**1a**) as a benchmark. The vinyl-substituted derivative **4a** exhibits an absorption maximum of λ_max_ = 331 nm, slightly red-shifted from the chloro-analogue **1a** for which no absorption maximum can be observed above 300 nm. This can be rationalized by the interaction of the alkenyl with the tetrel-center, as per the predictions of Apeloig et al.: interaction of the partially filled p-orbital at Ge overlaps with the low-lying π*-orbital of the vinyl unit, lowering the energy of the LUMO, and reducing Δ*E*_HOMO/LUMO_. The near-UV absorption displayed by **4a** nevertheless corresponds to a large Δ*E*_HOMO/LUMO_ for this system, calculated to be 212.2 kcal·mol^–1^ by DFT methods in model complex **4’** (**4’** = L’(C_2_H_3_)­Ge:; L’ = {[Me_2_PCH_2_Si­(Me)_2_]­(Xyl)­N}^−^; Xyl = 2,6-Me_2_C_6_H_3_). Here, however, one can clearly observe delocalization of the Ge-centered *p*-orbital over the π*-system of the vinyl ligand as the LUMO, while the HOMO represents a nonbonding electron pair at Ge (see Figure S75 in ESI). Comparing the UV–vis spectra of **4a**, **5b**, and **6b**, a slightly more pronounced bathochromic effect can be observed on descending group 14. While the vinyl-stannylene **5a** exhibits a maximum absorption at λ_max_ = 339 nm, differing only slightly from its germylene analogue **4a**, the lead-containing compound **6a** shows a more pronounced red shift with λ_max_ = 368 nm. A similar trend was observed in comparable systems bearing N-heterocyclic-vinyl ligands.^41^ However, in several aryl-based tetrylenes, the trend is reversed, with germanium having a more pronounced red shift, decreasing with tin and lead.^56,57^ This would suggest that the observed bathochromic effect is not an intrinsic property of heavier low-valent group 14 species, but rather directly related to the unsaturated vinyl substituent.

**Figure 4:**
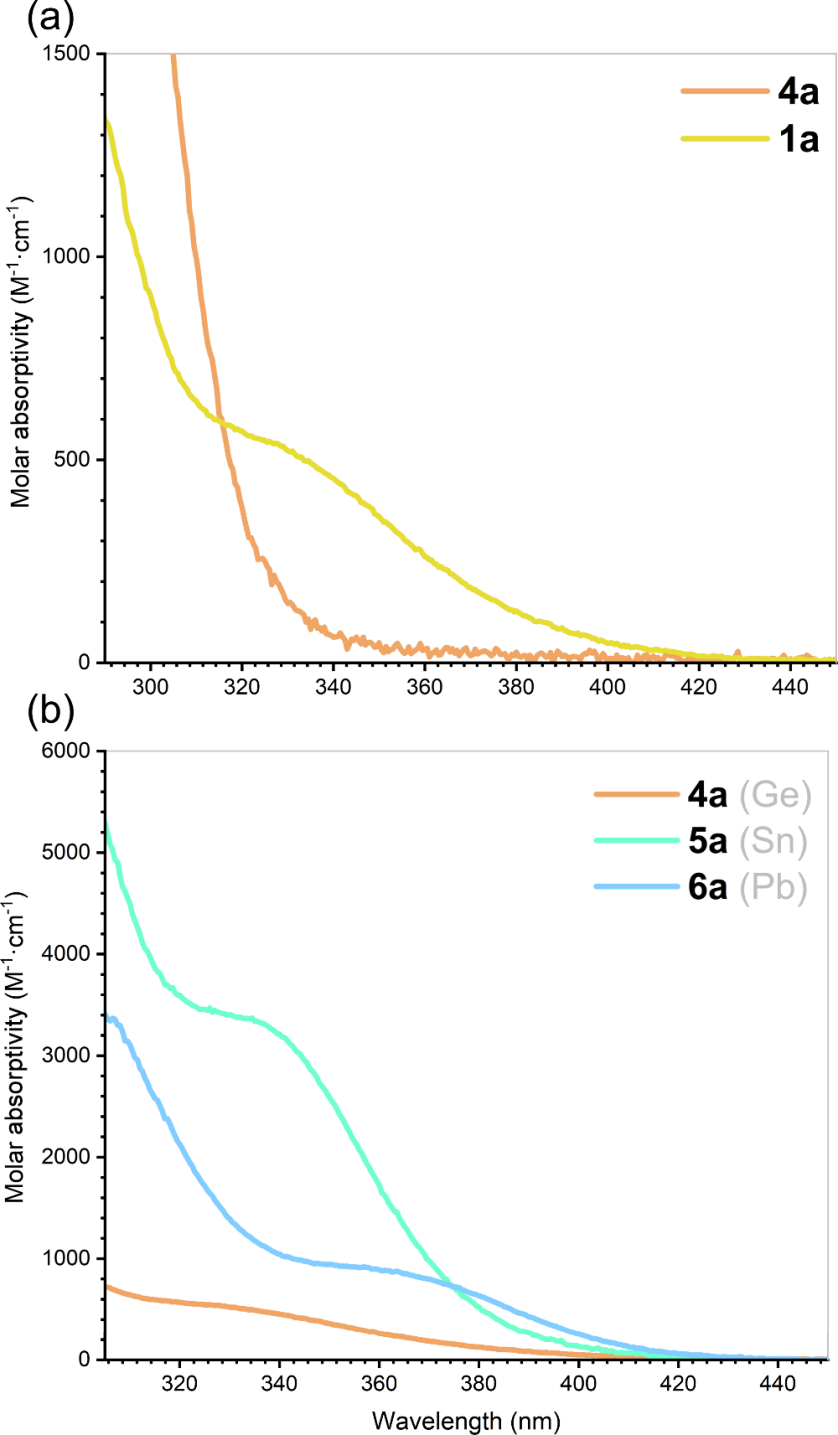
UV–vis spectra of (a) **1a** and **4a**; (b) vinyl-tetrylenes **4a**, **5a**, and **6a**.

### Group Elimination in Formation of a Pb^II^ Cation

Following the successful isolation of vinyl-tetrylenes **4**-**6**, we sought to begin exploring their utility. That began with the attempted generation of cationic tetrylenes, [^R^LE:]^+^, as an extension to our earlier synthesis of [^Ph^LE:]^+^ through hydride abstraction from the element­(II) hydride congeners ^Ph^L­(H)­E: (E = Ge, Sn),^48^ with the trityl cation, [Ph_3_C]^+^. Compounds featuring low-coordinate cationic tetrylene units are known, but remain uncommon.^58–63^ Lead derivatives are perhaps the most rare, despite Jutzi et al. reporting the first cationic half-sandwich [Pb^II^]^+^ complex in 1989 (N.B. that species in fact has short bridging Pb···F­(BF_2_)­F···Pb interactions [d_PbF_ = 2.831(9) and 2.901(9) Å]).^64^ Nevertheless, over the past years, synthetic methods were developed to access highly electrophilic, cationic Pb^II^ species.^55,65,66^ Notably, we have previously been unable to access the phosphine-appended lead­(II) cations, [^R^LPb:]^+^, as halide abstraction is not possible, and the hydrido-plumbylene is not stable, impeding our developed hydride abstraction pathway. Therefore, we first attempted to access [^Ph^LPb]­[BAr^F^_4_] (**7**) via vinyl elimination from **6** with the strong electrophile [Ph_3_C]­[BAr^F^_4_] (Figure 5). This would proceed through hydride abstraction, and elimination of C_2_H_2_, in a similar vein to alkaline-earth cation chemistry,^67–70^ and related group 14 element­(IV) cations which utilize allyl elimination.^71,72^ Addition of [Ph_3_C]­[BAr^F^_4_] to cooled solutions of **6a** led to an immediate color change to dark orange, further intensifying over the course of two hours. An *in situ* ^31^P­{^1^H} NMR analysis indicated the presence of an extremely downfield-shifted peak at δ = 132.5 ppm, attributed to the lead cation, whereby the highly electrophilic heavy atom significantly deshields the phosphorus center. Ph_3_CH is additionally observed in the ^1^H NMR spectra of crude mixtures. Deep yellow-orange single crystals with a yield of 62% were grown from these reaction mixtures, which confirmed the successful synthesis of **7** through X-ray diffraction analysis (Figure 5). The lead center in **7** is coordinated and therefore stabilized by the chelating ligand. However, the distance between the Pb atom and a fluorine atom of the weakly coordinating [BAr^F^_4_]^−^ is 3.40 Å, which is only 0.09 Å smaller than the sum of van der Waals radii of 3.49 Å but longer than the expected length of a covalent bond around 2.08 Å,^73,74^ indicating possible weak interaction and thereby further stabilization by a fluoride of the [BAr^F^_4_]^−^ counteranion. With this additional fluoride contact, we rationalize the existence of a second species observed in NMR spectra of dissolved crystals of **7**, these solutions always exhibiting the same ratio in solution. This would indicate a dynamic process in solution, e.g. between the free cation, and the weak Pb···F–C contact pair. This is supported by ^19^F­{^1^H} NMR: besides the expected shift of – 62.9 ppm for the counteranion,^75^ another resonance at – 62.8 ppm is observed. The presence of this slightly more deshielded signal can be an indication of a fluoride from the counteranion coordinating to the cationic lead species. In addition, a second ^31^P NMR shift is observed at 33.5 ppm, this relative upfield shift supporting a high coordinate cationic Pb^II^ center.

**Figure 5:**
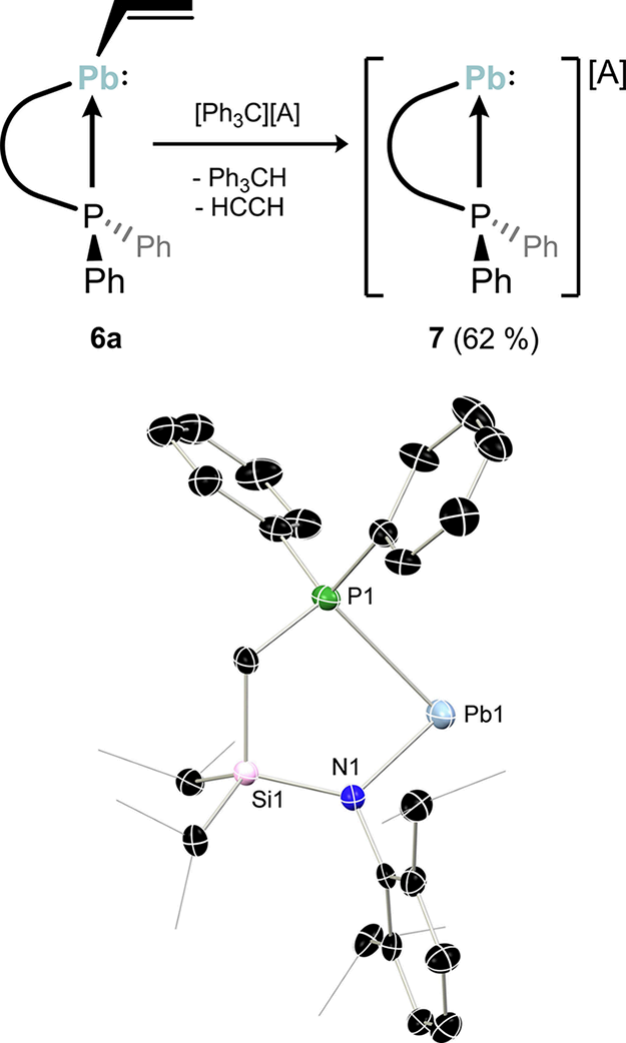
(a) Reaction of vinyl plumbylene **6a** with [Ph_3_C]­[BAr^F^_4_] forming cationic **7** (A = BAr^F^_4_); (b) molecular structure in the solid state of the cationic part of **7** with thermal ellipsoids at 30% probability, and hydrogen atoms omitted for clarity. Selected bond distances (Å) and angle (°) for **7**: Pb1–P1 2.743(2); Pb1–N1 2.133(6); N1–Pb1–P1 81.1(1); Pb1···F13 3.401(7) (see Figure S74 in ESI).

Comparing the bond lengths of the cationic species **7** with the corresponding lead-bromide **3a**, a distinct shortening of the Pb–N and Pb–P bonds is observed (**3a**: d_Pb–N_ = 2.253(6) Å, d_Pb–P_ = 2.815(2) Å); **7**: d_Pb–N_ = 2.133(6) Å, d_Pb–P_ = 2.743(2) Å), aligning the electrophilicity of the Pb center in the former. Following the successful synthesis of **7**, we sought to generate the known Ge^II^ and Sn^II^ cations [^Ph^LGe:]­[BAr^F^_4_] and [^Ph^LSn:]­[BAr^F^_4_] via a similar methodology, particularly since the latter cannot be obtained by a simple salt metathesis, but only via its related hydride which itself shows instability in solution.^48^ Surprisingly, though a reaction is observed between both **4a** and **5a**, and [Ph_3_C]­[BAr^F^_4_], the lighter group 14 cations could not be observed, and rather complicated reaction mixtures are found, and hence cannot be obtained via this approach.

### Transition Metal Coordination of a Vinyl-tetrylene

Finally, given the discussed unique electronic nature of the vinyl tetrylenes **4**-**6**, we aimed to synthesize the first examples of their metal coordination complexes. To this end, **4a** and **4b** were reacted with two different Ni^0^ species, targeting both mono- and bis-tetrylene complexes of this metal (Scheme 2). The reaction of one equiv of **4a** with [(PPh_3_)_2_Ni­(cod)] led to an immediate color change to dark red. *In situ* ^31^P­{^1^H} NMR spectroscopic analysis revealed full consumption of **4a**, and new triplet and doublet signals at δ = 9.34 and 42.1 ppm, respectively; the observed multiplicity indicates the formation of target complex **8**, confirmed by X-ray analysis of single crystals of this complex (Figure 6(a)). Now utilizing two equiv. **4a**, and Ni­(cod)_2_ in place of [(PPh_3_)_2_Ni­(cod)], the bis­(vinyltetrylene)-Ni^0^ complex **9** was accessed (Figure 6(b)), supported by ^31^P­{^1^H} NMR spectroscopic analysis, which indicates a single new peak at 35.4 ppm. The Ni^0^ center in the solid-state structure of **8** is tetracoordinate, coordinated by the chelating vinyl-germylene ligand and two triphenylphosphine ligands in a tetrahedral fashion. Since the angles around the transition metal center deviate from the ideal tetrahedral angle of 109.47° (e.g., Ge1–Ni1–P1 97.85(7)°), the coordination around it can be seen as a distorted tetrahedral. The vinyl-germylene unit lies in a plane with the amido-group, evidenced by the sum of angles around germanium being 360°. The planarity of this unit is indicative of favorable π-orbital overlap between the nitrogen, germanium and vinyl substituent, which presumably allows for electron delocalizaton over these centers. The C = C bond length is elongated compared to the respective vinyl-tetrylene **4a**, indicating a less pronounced donation from germanium to the vinyl group, due to Ge→Ni donation. Comparison with the literature-known complexes ^R^L­(X)­Ge·Ni·(PPh_3_)_2_ (X = Cl, NH_2_, OH) reveals a relative elongation of core metrical parameters in **8**.^47^ For example, in the chloro-germylene derivative, a Ge1–Ni1 distance of 2.188(1) Å is observed, being slightly longer than in **8** (2.229(1) Å). The same is true for the Ge1–N1 bond, with a length shortened by 0.02 Å compared to the herein reported complex. Similar trends are observed within the -NH_2_ and −OH substituted germylene-nickel compounds. This is in agreement with donation from germanium to the vinyl-substituent, leaving the germanium center less electron-rich than in comparable compounds and therefore contributing to an elongation of the Ge–Ni bond. Consistent with this trend, the bond metrics in **8** closely resemble those reported for the related aryl-substituted germylene ^Ph^LGe­(Ph)·Ni·IPr (IPr = [{(H)­CN­(Dipp)}_2_C:]), which features Ge1–Ni1 and Ge1–N1 distances of 2.219(1) and 1.898(8) Å, respectively.^76^ The close similarity underscores that conjugated substituents at germanium exert a distinct influence on the electronic and structural parameters of these complexes.

**Scheme 2:**
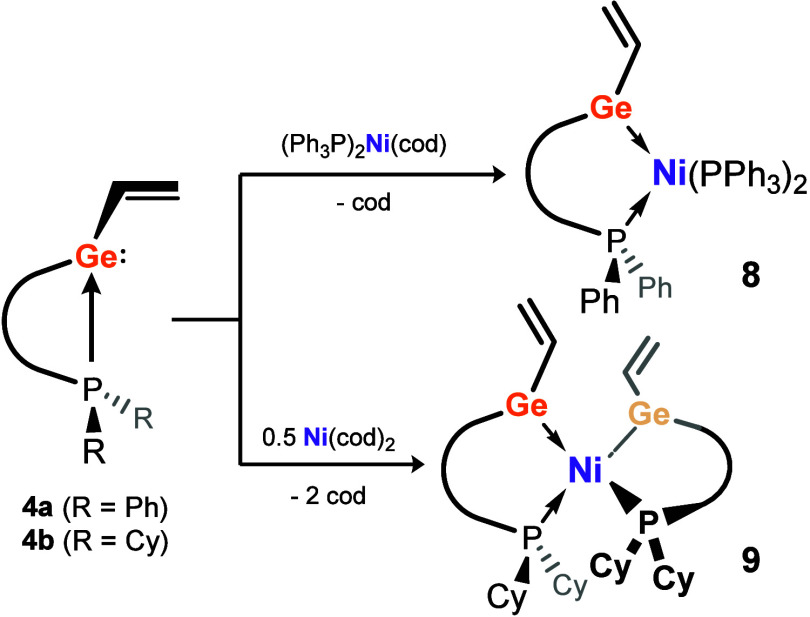
Complexation of Ni^0^ Reagents with **4a** and **4b**

**Figure 6:**
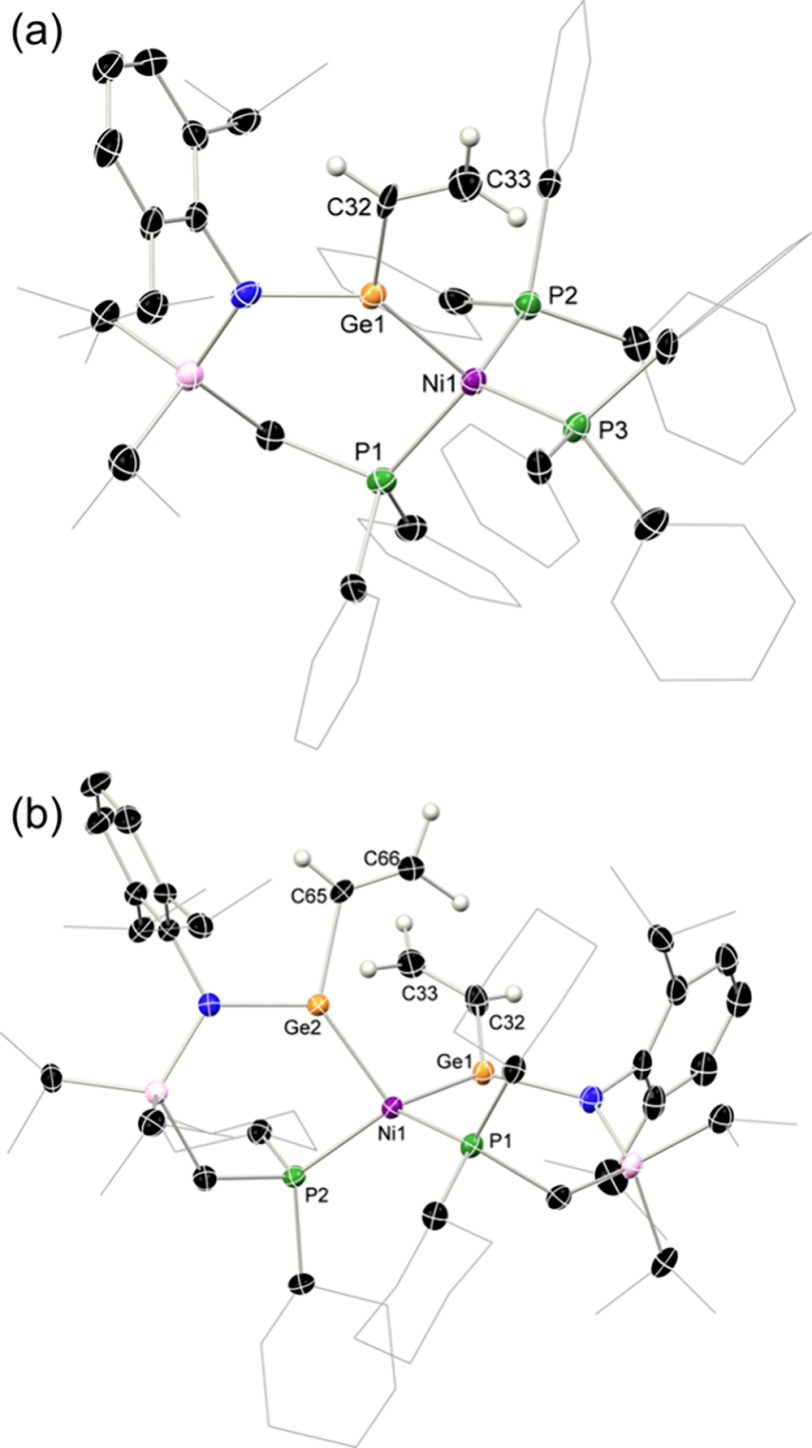
Molecular structures in the solid state of (a) **8** and (b) **9**, with thermal ellipsoids at 30% probability, and hydrogen atoms omitted for clarity. Selected bond distances (Å) and angle (°) for **8**: Ge1–C32 1.953(9); Ge1–Ni1 2.229(1); Ge1–N1 1.891(6); Ni1–P1 2.206(2); Ge1–Ni1–P1 99.80(1); Ge1–Ni1–P2 108.33(7); Ge1–Ni1–P3 109.38(7). For **9**: Ge1–C32 1.981(7); Ge1–Ni1 2.227(1); Ge2–Ni1 2.215(1); Ge1–N1 1.895(1); P1–Ni1 2.200(2); Ge1–Ni1–Ge2 109.14(4); Ge1–Ni1–P1 97.85(6); Ge2–Ni1–P1 115.26(6).

Despite the observed elongation of the Ge–Ni bond length in **8** relative to our earlier reported Ni^0^ germylene complexes ^Ph^L­(X)­Ge·Ni­(PPh_3_)_2_, e.g. where X = Cl or NH_2‑_, a computational analysis, using the L’ ligand in place of ^Ph^L, suggests that **8** does in fact have a stronger Ge–Ni bonding interaction, with a Mayer Bond Order (MBO) of 1.136, comparing to 1.083 (X = Cl) and 1.036 (X = NH_2_). A Second-Order Perturbation Theory analysis suggests that the vinyl-substituted germylene ligand in **8** is a stronger σ-donor than even the -NH_2_ derivative, with a total Ge→Ni interaction of 127.5 kcal·mol^–1^ for the former, vs 119.0 kcal·mol^–1^ for the latter, and 102.3 kcal·mol^–1^ for the -Cl derivative. Given that Second-Order Perturbation Theory does not energetically define interactions across multiple acceptor units, the Ni→Ge back-donation energies are likely underestimated for **8**; nevertheless, a value for **8** of 31.1 kcal·mol^–1^ is found, comparing to 27.5 kcal·mol^–1^ and 36.7 kcal·mol^–1^ for the -NH_2_ and -Cl derivatives, respectively. Looking to the calculated LUMO for **8**, a clear combination of the Ge-centered *p*-orbital with the π*-orbital of the vinyl unit is observed, forming an empty Ge–C π-orbital (Figure 7). Ge–Ni bonding is seen clearly in both the HOMO (σ-bond) and HOMO–1 (π-bond), accounting for interactions described above.

**Figure 7:**
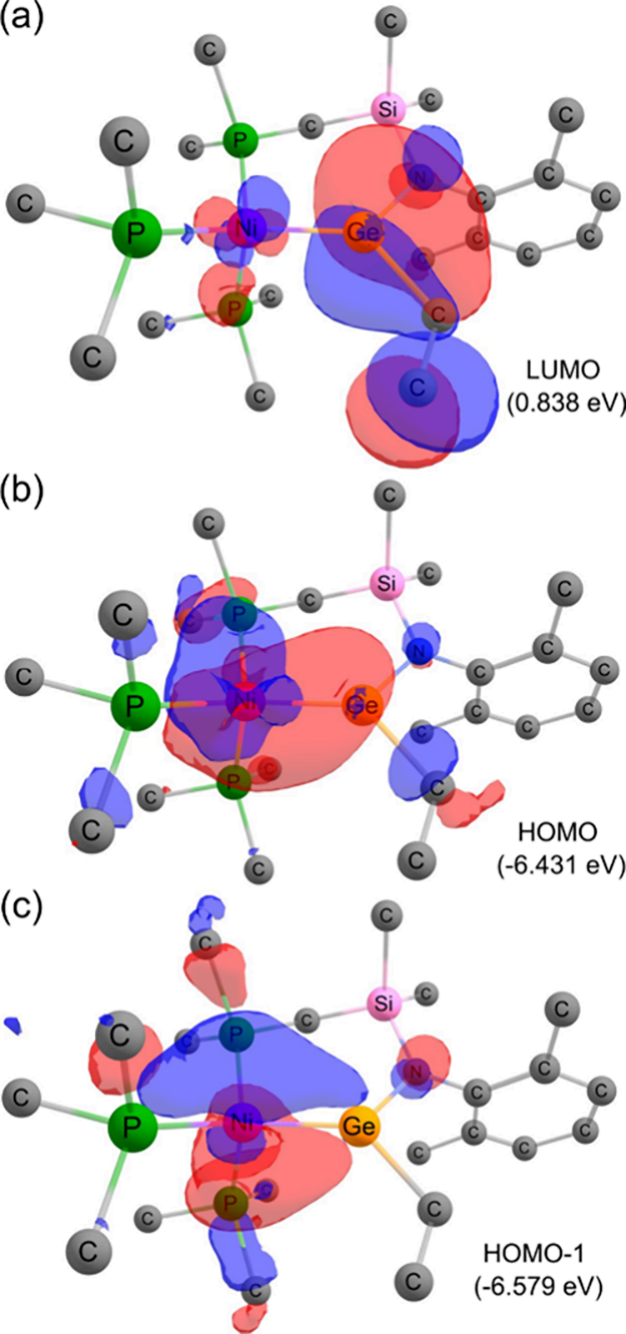
Computationally derived frontier orbitals of 8’. Calculations were carried out at the ωB97D3//def2SVP­(Ge,Ni,P: def2-TZVPP) level.

Now looking to complex **9**, the nickel center features a slightly distorted tetrahedral geometry, with angles of Ge1–Ni1–Ge2 = 109.14(4)°, Ge1–Ni1–P1 = 97.85(6)° and Ge2–Ni1–P1 = 115.26(6)°, while the coordination sphere around the germanium centers is trigonal planar, with the sum of angles around germanium of exactly 360°, analogous to **8**. Both germanium units are essentially equidistant from Ni (d_Ge1–Ni1_ = 2.227(1) Å; d_Ge2–Ni1_ = 2.215(1) Å), and lie in plane with the respective vinyl substituent and the nitrogen donor of the chelating ligand, thereby enabling delocalization of electron density across these atoms and contributing to the stabilization of the low-valent germylene centers. In this coordination environment, the Ge–Ni as well as the Ge–N bond lengths in **9** are slightly elongated compared to those in **8**. This elongation can be attributed to the presence of two germylene donors coordinated to the same nickel atom, resulting in reduced Ni→Ge back-donation per individual germylene unit. Notably, the observed bond lengths are significantly longer than in the previously discussed germylene-nickel(0) complexes, further supporting the assignment of a highly electron-rich Ni^0^ species in **9**.^15,76^ In solution, both vinyl substituents are chemically equivalent, as evidenced by ^1^H NMR spectroscopy: three doublets of doublets at 6.04 (^3^ *J* = 12.5 Hz, ^2^ *J* = 3.4 Hz), 6.18 (^3^ *J* = 20.4 Hz, ^2^ *J* = 3.4 Hz) and 6.43 ppm (^3^ *J* = 20.4 Hz, ^3^ *J* = 12.5 Hz) are observed for solutions of **9**. These signals correspond to the six vinylic protons; the α-proton with respect to the heavy group 14 atom exhibits the most deshielded peak at 6.43 ppm, showing H–H_trans_ coupling of 20.4 Hz, while the other two peaks can be assigned to the protons at the β-carbon. Compared to the vinyl-tetrylenes **4**-**6**, the vinylic signals do not show any indication of H–P coupling, due to the insertion of a nickel atom into the Ge–P bond. As a whole, the isolation and characterization of complexes **8** and **9** marks a new entry into the coordination chemistry of tetrylenes, further expanding our understanding of their electronic nature.

## Conclusions

In conclusion, the successful isolation of a series of half-parent vinyl-tetrylenes is described, for Ge, Sn, and Pb, synthesized through a straightforward salt-methathesis process using vinyl Grignard reagents. Spectroscopic and computational characterization of these species gives key insights into the conjugative electronic effects of the vinyl ligand. In addition, we demonstrate the utility of such species in the synthesis of a cationic plumbylene **7**, through hydride abstraction and C_2_H_4_ elimination, as well as the first transition metal coordination complexes of vinyl-tetrylenes. The facile synthesis of this new ligand class, in addition to a developed understanding of the effects of the vinyl group, opens the door to further investigations utilizing this synthetic strategy.

## Experimental Section

### General Considerations

All experiments and manipulations were carried out under dry oxygen-free argon atmosphere using standard Schlenk techniques or in a MBraun inert atmosphere glovebox containing an atmosphere of high-purity argon. C_6_D_6_ was dried, degassed and stored over a potassium mirror. THF and Et_2_O were dried over Na/Benzephenone, distilled and stored over 4Å molecular sieves. All other solvents were dried over activated 4 Å molecular sieves and thoroughly degassed before use. ^Ph^LK,^47 Cy^LK,^77^ **1a**,^47^ **2a**,^15^ Ni­(cod)_2_,^78^ and (cod)­Ni­(PPh_3_)_2_ ^79^ were synthesized using reported procedures. All other reagents were used as received. NMR spectra were recorded on a Bruker AV 400 Spectrometer. ^1^H and ^13^C­{^1^H} NMR spectra were referenced to the residual solvent signals as internal standards. ^29^Si­{^1^H} NMR spectra were externally calibrated with SiMe_4_. ^31^P­{^1^H} NMR spectra were externally calibrated with H_3_PO_4_. Liquid Injection Field Desorption Ionization Mass Spectrometry (LIFDI-MS) was measured directly from an inert atmosphere glovebox with a Thermo Fisher Scientific Exactive Plus Orbitrap equipped with an ion-source from Linden CMS.^80^ Commercial hydrogen gas with a purity of ≥ 99.999% was used in all hydrogenation experiments. Elemental analyses (C, H, N) were performed with a combustion analyzer (elementar vario EL, Bruker). Absorption spectra (UV/vis) were recorded on an Agilent Cary 60 UV/vis spectrophotometer. Syntheses for compounds **1b**, **2b**, **3a**, and **3b** can be found in the Supporting Information. All other syntheses are included here. No uncommon hazards are noted.

### General Method for Preparation of ^R^L­(C_2_H_3_)­E: (*i.e.*, **4**–**6**)

To a solution of ^R^L­(X)­E: (1.0 eq.; R = Ph, Cy, X = Cl, Br) in THF (0.1 mL/mg), vinyl-magnesium bromide (1.0 M in THF, 1.2 equiv) was added dropwise at −80 °C. The solution was allowed to gradually warm overnight with stirring, leading to a dark orange solution in all cases. After this time, all volatiles were removed *in vacuo*, the mixture suspended in toluene (0.1 mL/mg of tetrylene starting material), and 1,4-dioxane (1.21 equiv) was added to aid in precipitation of magnesium salts. Filtration and the subsequent removal of all volatiles *in vacuo* yielded a pale yellow-orange solid, which was suspended in a small amount of pentane (e.g., 2 mL/100 mg tetrylene starting material), and the supernatant filtered off and discarded. The resulting analytically pure off-white powders are dried *in vacuo*, yielding the desired product.

#### ^Ph^L­(C_2_H_3_)­Ge: **4a**

The germylene was synthesized according to the general method using ^Ph^L­(Cl)­Ge: (**1a**; 500 mg, 0.84 mmol). An off-white powder of **4a** (356 mg, 0,61 mmol, 72%) was isolated. Colorless crystals suitable for X-ray diffraction analysis were obtained from a concentrated diethyl ether solution, which was stored at 7 °C for 3 days.

**^1^** **H NMR** (C_6_D_6_, 400 MHz, 298 K): δ = 0.73 (d, 3H, ^3^J_HH_ = 7.3 Hz, Si–Pr^i^–C*H*_3_), 0.99 (d, 4H, ^3^J_HH_ = 7.2 Hz, Dipp–Pr^i^–C*H*_3_, Si–Pr^i^–C*H*), 1.11 (d, 3H, ^3^J_HH_ = 7.6 Hz, Si–Pr^i^–C*H*_3_), 1.17 (d, 3H, ^3^J_HH_ = 6.3 Hz, Dipp–Pr^i^–C*H*_3_), 1.22–1.33 (overlapping m, 9H, Si–Pr^i^–C*H*_3_, Dipp–Pr^i^–C*H*_3_), 1.39–1.41 (overlapping m, 4H, Dipp–Pr^i^–C*H*_3_, Si–Pr^i^–C*H*), 1.85 (t, 2H, ^2^J_HP_ = 14.5 Hz, Ph_2_P–C*H*_2_), 3.04 (br sept, 1H, Dipp–Pr^i^–C*H*), 4.07 (br sept, 1H, Dipp–Pr^i^–C*H*), 5.96 (dt, 1H, ^3^J_HH_ = 21.0 Hz, ^2^J_HH_ = 3.4 Hz, CH = C*H*_2_), 6.13 (dd, 1H, ^3^J_HH_ = 13.1 Hz, ^2^J_HH_ = 3.4 Hz, CH = C*H*_2_), 6.80–7.10 (br m, 6H, Ar–C*H*), 7.10–7.25 (br m, 3H, Ar–C*H*), 7.35 (m, 2H, Ar–C*H*), 7.66 (ddd, 1H, ^3^J_HH_ = 13.1 Hz, ^2^J_HH_ = 3.4 Hz, ^3^J_HP_ = 1.8 Hz, C*H*=CH_2_), 7.71–7.85 (br m, 2H, Ar–C*H*).

**^13^** **C­{** **^1^** **H} NMR** (C_6_D_6_, 101 MHz, 298 K) δ = 6.2 (d, ^1^J_CP_ = 4.0 Hz, Ph_2_P–*C*H_2_), 15.8 and 16.7 (Si–Pr^i^–*C*H), 17.9 (Si–Pr^i^–*C*H_3_), 19.4 and 19.7 (Dipp–Pr^i^–*C*H_3_), 20.5 (Si–Pr^i^–*C*H_3_), 22.8 and 22.9 (Dipp–Pr^i^–*C*H_3_), 27.1 (Dipp–Pr^i^–*C*H), 27.7 (Si–Pr^i^–*C*H_3_), 28.1 (Dipp–Pr^i^–*C*H), 123.0, 123.6, 123.8, and 128.5 (Ar–*C*), 129.1 (d, ^3^J_CP_ = 16.9 Hz, CH = *C*H_2_), 129.9, 130.7, 131.7, and 132.8 (Ar–*C*), 158.5 (*C*H = CH_2_).

**^31^** **P­{** **^1^** **H} NMR** (C_6_D_6_, 162 MHz, 298 K): δ = 1.3 (s, *P*-Ph_2_).

**^29^** **Si­{** **^1^** **H} NMR** (C_6_D_6_, 99 MHz, 298 K): δ = 10.9 (d, ^2^J_SiP_ = 12.7 Hz, CH_2_–*Si*-^i^Pr_2_).

**MS/LIFDI-HRMS** found (calcd.) *m*/*z*: 589.2325 (589.2349) for [M].

**λ** **_max_**, nm (ε, L mol^–1^ cm^–1^): 331 (521).

**Anal. Calcd** for C_33_H_46_GeNPSi: C, 67.36%; H, 7.88%; N, 2.38%; found C, 65.80%; H, 7.64%; N, 2.43%.

N.B. Repeated element analysis gave variable but consistently low values for C, possibly due to Si carbide formation.

#### ^Cy^L­(C_2_H_3_)­Ge: **4b**

The germylene was synthesized according to the general method using ^Cy^L­(Cl)­Ge: (**1b**; 600 mg, 0.99 mmol). A colorless powder of **4b** (408 mg, 0.68 mmol, 69%) was isolated.

**^1^** **H NMR** (C_6_D_6_, 400 MHz, 298 K): δ = 0.73 (d, 3H, ^3^J_HH_ = 7.2 Hz, Si–Pr^i^–C*H*_3_), 1.00–1.13 (overlapping m, 9H_,_ Si–Pr^i^–C*H*_3_, Cy-*H*), 1.14–1.22 (overlapping m, 6H, Si–Pr^i^–C*H*, Si–Pr^i^–C*H*_3_, Cy-*H*), 1.22–1.28 (m, 6H, Dipp–Pr^i^–C*H*_3_, Cy-*H*), 1.33–1.37 (m, 5H, Si–Pr^i^–C*H*_3_, Cy-*H*), 1.39–1.43 (m, 6H, Dipp–Pr^i^–C*H*_3_), 1.49–1.52 (m, 4H, Si–Pr^i^–C*H*, Dipp–Pr^i^–C*H*_3_), 1.57–1.59 (m, 3H, Cy-*H*), 1.70–1.74 (m, 4H, Cy-*H*), 1.86–1.88 (m, 3H, Ph_2_P–C*H*_2_, Cy-*H*), 2.33–2.28 (m, 1H, Cy-*H*), 3.53 (sept, 1H, ^3^J_HH_ = 6.5 Hz, Dipp–Pr^i^–C*H*), 4.01 (sept, 1H, ^3^J_HH_ = 6.9 Hz, Dipp–Pr^i^–C*H*), 6.06 (dt, 1H, ^3^J_HH_ = 21.0 Hz, ^2^J_HH_ = 3.7 Hz, CH = C*H*_2_), 6.22 (dd, 1H, ^3^J_HH_ = 13.0 Hz, ^2^J_HH_ = 3.7 Hz, CH = C*H*_2_), 7.14–7.25 (m, 3H, Ar-*H*), 7.80 (ddd, 1H, ^3^J_HH_ = 21.0 Hz, ^3^J_HH_ = 13.0 Hz, ^3^J_HP_ = 3.5 Hz, C*H*=CH_2_).

**^13^** **C­{** **^1^** **H} NMR** (C_6_D_6_, 101 MHz, 298 K) δ = 0.7 (d, ^1^J_CP_ = 4.0 Hz, Cy_2_P–*C*H_2_), 17.0 (d, J_PC_ = 5.1 Hz, Cy-*C*H_2_), 18.2, 19.4, 20.2, and 20.8 (Si–Pr^i^–*C*H_3_), 23.4 and 23.9 (Dipp–Pr^i^–*C*H_3_), 26.3 and 26.5 (Si–Pr^i^–*C*H), 27.1 (Dipp–Pr^i^–*C*H), 27.5 (Cy-*C*H_2_), 27.6 (Dipp–Pr^i^–*C*H), 27.7, 27.9 (Cy-*C*H_2_), 28.0 (d, J_PC_ = 5.9 Hz, Cy-*C*H_2_), 28.1 (Cy-*C*H_2_), 28.4 (d, J_PC_ = 4.8 Hz, Cy-*C*H_2_), 29.6 (Dipp–Pr^i^–*C*H_3_), 29.8 (d, J_PC_ = 2.6 Hz, Cy-*C*H), 30.6 (d, J_PC_ = 2.6 Hz, Cy-*C*H), 123.5, 123.7, and 124.1 (Ar-*C*), 128.7 (d, ^3^J_CP_ = 16.5 Hz, CH = *C*H_2_), 160.3 (d, ^2^J_CP_ = 2.6 Hz, *C*H = CH_2_).

**^31^** **P­{** **^1^** **H} NMR** (C_6_D_6_, 162 MHz, 298 K): δ = 12.2 (s, *P*-Cy_2_).

**^29^** **Si­{** **^1^** **H} NMR** (C_6_D_6_, 99 MHz, 298 K): δ = 11.3 (d, ^2^J_SiP_ = 9.8 Hz, CH_2_–*Si*-^i^Pr_2_).

**MS/LIFDI-HRMS** found (calcd.) *m*/*z*: 574.3010 (574.3053) for [M-C_2_H_3_].

**Anal. Calcd** for C_33_H_58_GeNPSi: C, 66.00%; H, 9.74%; N, 2.33%; found C, 64.50%; H, 9.67%; N, 2.45%.

N.B. Repeated element analysis gave variable but consistently low values for C, possibly due to Si carbide formation.

#### ^Ph^L­(C_2_H_3_)­Sn: **5a**

The stannylene was synthesized according to the general method using ^Ph^LSnCl **2a** (450 mg, 0.70 mmol). An off-white powder of **5a** (290 mg, 0.46 mmol, 65%) was isolated.

**^1^** **H NMR** (C_6_D_6_, 400 MHz, 298 K): δ = 0.77 (d, 3H, ^3^J_HH_ = 7.3 Hz, Si–Pr^i^–C*H*_3_), 0.99–1.05 (m, 1H, Si–Pr^i^–C*H*), 1.11 (d, ^3^J_HH_ = 6.8 Hz, Dipp–Pr^i^–C*H*_3_), 1.13–1.15 (m, 6H, Si–Pr^i^–C*H*_3_, Dipp–Pr^i^–C*H*_3_), 1.26–1.31 (m, 9H, Si–Pr^i^–C*H*_3_, Dipp–Pr^i^–C*H*_3_), 1.40 (d, ^3^J_HH_ = 7.0 Hz, Dipp–Pr^i^–C*H*_3_, Si–Pr^i^–C*H*), 1.86–1.95 (m, 2H, Ph_2_P–C*H*_2_), 3.16 (sept, 1H, ^3^J_HH_ = 6.8 Hz, Dipp–Pr^i^–C*H*), 4.19 (sept, 1H, ^3^J_HH_ = 6.8 Hz, Dipp–Pr^i^–C*H*), 5.96 (ddd, 1H, ^3^J_HH_ = 21.0 Hz, ^2^J_HH_ = 3.5 Hz, ^4^J_HP_ = 3.0 Hz, CH = C*H*_2_), 6.45 (dd, 1H, ^3^J_HH_ = 13.9 Hz, ^3^J_HH_ = 3.5 Hz, CH = C*H*_2_), 6.95–7.16 (m, 8H, Ar–C*H*), 7.19–7–20 (m, 1H, Ar–C*H*), 7.36–7.42 (m, 2H, Ar–C*H*), 7.60–7.69 (m, 2H, Ar–C*H*), 8.03 (ddd, 1H, ^3^J_HH_ = 21.0 Hz, ^3^J_HH_ = 13.9 Hz, ^3^J_HP_ = 3.0 Hz, C*H*=CH_2_).

**^13^** **C­{** **^1^** **H} NMR** (C_6_D_6_, 101 MHz, 298 K) δ = 9.2 (d, ^1^J_CP_ = 6.6 Hz, Ph_2_P–*C*H_2_), 16.5 (d, ^3^J_CP_ = 5.5 Hz, Si–Pr^i^–*C*H), 17.6 (d, ^3^J_CP_ = 2.2 Hz, Si–Pr^i^–*C*H), 18.5, 19.8, 19.9, and 21.0 (Si–Pr^i^–*C*H_3_), 23.2 and 23.5 (Dipp–Pr^i^–*C*H_3_), 27.3 (Dipp–Pr^i^–*C*H), 27.7 and 28.0 (Dipp–Pr^i^–*C*H_3_), 28.4 (Dipp–Pr^i^–*C*H), 123.1, 123.4, 124.1 (Ar–*C*), 129.0 (d, J_CP_ = 9.2 Hz, Ar–*C*), 129.1 (d, J_CP_ = 9.5 Hz, Ar–*C*), 130.4 (d, J_CP_ = 2.6 Hz, Ar–*C*), 131.0 (d, J_CP_ = 2.2 Hz, Ar–*C*), 132.2 (d, ^3^J_CP_ = 12.1 Hz, CH = *C*H_2_), 132.3 (d, J_CP_ = 11.7 Hz, Ar–*C*), 133.3 (d, J_CP_ = 12.8 Hz, Ar–*C*), 168.6 (*C*H = CH_2_).

^31^ **P­{** **^1^** **H} NMR** (C_6_D_6_, 162 MHz, 298 K): δ = −6.3 (s, *P*-Ph_2_, ^1^J_117SnP_ = 609 Hz, ^1^J_119SnP_ = 637 Hz).

**^29^** **Si­{** **^1^** **H} NMR** (C_6_D_6_, 99 MHz, 298 K): δ = 8.2 (d, ^2^J_SiP_ = 10.3 Hz, CH_2_–*Si*-^i^Pr_2_).

**MS/LIFDI-HRMS** found (calcd.) *m*/*z*: 608.1878 (608.1924) for [M-C_2_H_3_].

**λ** **_max_**, nm (ε, L mol^–1^ cm^–1^): 339 (3216).

**Anal. Calcd** for C_33_H_46_NPSiSn: C, 62.47%; H, 7.31%; N, 2.21%; found C, 62.10%; H, 7.46%; N, 2.25%.

#### ^Cy^L­(C_2_H_3_)­Sn: **5b**

The stannylene was synthesized according to the general method using ^Cy^LSnCl **2b** (300 mg, 0.46 mmol). A colorless powder of **5b** (179 mg, 0,28 mmol, 60%) was isolated. Colorless crystals suitable for X-ray diffraction analysis were obtained from a concentrated diethyl ether solution, which was stored at 7 °C for 3 days.

^1^ **H NMR** (C_6_D_6_, 400 MHz, 298 K): δ = 0.79 (d, 3H, ^3^J_HH_ = 7.1 Hz, Si–Pr^i^–C*H*_3_), 0.99–1.09 (m, 4H_,_ Cy-*H*), 1.10–1.13 (m, 5H, Si–Pr^i^–C*H*_3_, Cy-*H*), 1.14–1.25 (m, 7H, Si–Pr^i^–C*H*_3_, Cy-*H*), 1.28 (d, 6H, ^3^J_HH_ = 7.0 Hz, Dipp–Pr^i^–C*H*_3_), 1.32 (d, 3H, ^3^J_HH_ = 7.0 Hz, Si–Pr^i^–C*H*_3_), 1.39–1.43 (m, 10H, Dipp–Pr^i^–C*H*_3_, Si–Pr^i^–C*H*_3_, Si–Pr^i^–C*H*), 1.51–1.60 (m, 4H, Cy-*H*), 1.65–1.75 (m, 4H, Cy-*H*), 1.78–1.86 (m, 2H, Ph_2_P–C*H*_2_), 1.92–1.98 (m, 1H, Cy-*H*), 2.05–2.13 (m, 1H, Cy-*H*), 3.56 (sept, 1H, ^3^J_HH_ = 6.9 Hz, Dipp–Pr^i^–C*H*), 4.17 (sept, 1H, ^3^J_HH_ = 6.8 Hz, Dipp–Pr^i^–C*H*), 6.03 (ddd, 1H, ^3^J_HH_ = 21.0 Hz, ^2^J_HH_ = 3.8 Hz, ^4^J_HP_ = 3.4 Hz, CH = C*H*_2_), 6.50 (dd, 1H, ^3^J_HH_ = 13.8 Hz, ^2^J_HH_ = 3.8 Hz CH = C*H*_2_), 7.11–7.20 (m, 3H, Ar-*H*), 8.14 (ddd, 1H, ^3^J_HH_ = 21.0 Hz, ^2^J_HH_ = 13.8 Hz, ^3^J_HP_ = 1.8 Hz, C*H*=CH_2_).

**^13^** **C­{** **^1^** **H} NMR** (C_6_D_6_, 101 MHz, 298 K) δ = 2.51 (d, ^1^J_CP_ = 9.2 Hz, Cy_2_P–*C*H_2_), 18.5, 19.9, 20.1, and 20.9 (Si–Pr^i^–*C*H_3_), 23.4 and 24.0 (Dipp–Pr^i^–*C*H_3_), 26.3 (d, J_PC_ = 12.1 Hz, Cy-*C*H_2_), 27.4 (Cy-*C*H_2_), 27.5 (Si–Pr^i^–*C*H), 27.6 (d, J_PC_ = 3.3 Hz, Cy-*C*H_2_), 27.7, 27.8, and 28.0 (Cy-*C*H_2_), 28.1 (Dipp–Pr^i^–*C*H), 28.2 (Si–Pr^i^–*C*H), 28.5 (Dipp–Pr^i^–*C*H), 29.5 (d, J_PC_ = 2.2 Hz, Cy-*C*H_2_), 30.0 (d, J_PC_ = 2.6 Hz, Cy-*C*H_2_), 31.1 (d, ^1^J_PC_ = 2.6 Hz, Cy-*C*H), 34.8 (d, J_PC_ = 11.7 Hz, Cy-*C*H_2_), 35.3 (d, ^1^J_PC_ = 4.0 Hz, Cy-*C*H), 122.9, 123.6, and 124.0 (Ar–*C*), 131.7 (d, ^3^J_PC_ = 11.4 Hz, CH = *C*H_2_), 167.8 (d, ^2^J_PC_ = 2.9 Hz, *C*H = CH_2_).

^31^ **P­{** **^1^** **H} NMR** (C_6_D_6_, 162 MHz, 298 K): δ = 7.44 (s, ^1^J_117SnP_ = 650 Hz, ^1^J_119SnP_ = 681 Hz, *P*-Cy_2_).

**^29^** **Si­{** **^1^** **H} NMR** (C_6_D_6_, 99 MHz, 298 K): δ = 8.3 (d, ^2^J_SiP_ = 7.8 Hz, CH_2_–*Si*-^i^Pr_2_).

**MS/LIFDI-HRMS** found (calcd.) *m*/*z*: 620.2875 (620.2863) for [M-C_2_H_3_].

**Anal. Calcd** for C_33_H_58_NPSiSn: C, 61.30%; H, 9.04%; N, 2.17%; found C, 58.85%; H, 8.62%; N, 2.07%.

N.B. Repeated element analysis gave variable but consistently low values for C, possibly due to Si carbide formation.

#### ^Ph^L­(C_2_H_3_)­Pb: **6a**

The plumbylene was synthesized according to the general method using ^Ph^LPbBr **3a** (900 mg, 1.16 mmol). A colorless powder of **6a** (452, 0.63 mmol, 54%) was isolated.

**^1^** **H NMR** (C_6_D_6_, 400 MHz, 298 K): δ = 0.98–1.44 (br m, 26H, Si–Pr^i^–C*H*_3_, Si–Pr^i^–C*H*, Dipp–Pr^i^–C*H*_3_), 2.16 (d, 2H, ^2^J_HP_ = 13.6 Hz, Ph_2_P–C*H*_2_), 3.36 (br sept, 1H, Dipp–Pr^i^–C*H*), 4.25 (br sept, 1H, Dipp–Pr^i^–C*H*), 6.31 (ddd, 1H, ^3^J_HH_ = 20.8 Hz, ^2^J_HH_ = 2.8 Hz, ^4^J_HP_ = 1.8 Hz, CH = C*H*_2_), 6.92–7.12 (m, 7H, Ar–C*H*), 7.12–7.25 (m, 2H, Ar–C*H*), 7.44–7.51 (m, 4H, Ar–C*H*), 7.92 (dd, 1H, ^3^J_HH_ = 13.2 Hz, ^2^J_HH_ = 2.8 Hz, C*H*=CH_2_), 10.52 (ddd, 1H, ^3^J_HH_ = 20.8 Hz, ^3^J_HH_ = 13.2 Hz, ^3^J_HP_ = 1.5 Hz, C*H*=CH_2_).

**^13^** **C­{** **^1^** **H} NMR** (C_6_D_6_, 101 MHz, 298 K) δ = 14.9 (d, ^1^J_CP_ = 10.0 Hz, Ph_2_P–*C*H_2_), 18.2 (Si–Pr^i^–*C*H_3_/Dipp–Pr^i^–*C*H_3_), 18.3 (Si–Pr^i^–*C*H_3_/Si–Pr^i^–*C*H), 18.6 (Si–Pr^i^–*C*H_3_/Si–Pr^i^–*C*H), 19.8 (Si–Pr^i^–*C*H_3_/Si–Pr^i^–*C*H/Dipp–Pr^i^–*C*H_3_), 21.2 (Si–Pr^i^–*C*H_3_/Si–Pr^i^–*C*H/Dipp–Pr^i^–*C*H_3_), 24.0 (Si–Pr^i^–*C*H_3_/Si–Pr^i^–*C*H/Dipp–Pr^i^–*C*H_3_), 27.5 (Si–Pr^i^–*C*H_3_/Si–Pr^i^–*C*H/Dipp–Pr^i^–*C*H_3_/ Dipp–Pr^i^–*C*H), 122.4 and 123.4 (Ar-*C*), 129.0 (d, ^3^J_PC_ = 9.1 Hz, CH = *C*H_2_), 133.0 (d, J_CP_ = 13.6 Hz, Ar–*C*), 231.5 (d, ^2^J_PC_ = 10.4 Hz, *C*H = CH_2_).

^31^ **P­{** **^1^** **H} NMR** (C_6_D_6_, 162 MHz, 298 K): δ = 7.89 (s, ^1^J_207PbP_ = 898 Hz, *P*-Ph_2_).

**^29^** **Si­{** **^1^** **H} NMR** (C_6_D_6_, 99 MHz, 298 K): δ = 9.5 (d, ^2^J_SiP_ = 9.3 Hz, CH_2_–*Si*-^i^Pr_2_).

**MS/LIFDI-HRMS** found (calcd.) *m*/*z*: 696.2637 (696.2669) for [M-C_2_H_3_].

**λ** **_max_**, nm (ε, L mol^–1^ cm^–1^): 368 (820).

**Anal. Calcd** for C_33_H_46_NPSiPb: C, 54.82%; H, 6.41%; N, 1.94%; found C, 57.60%; H, 5.95%; N, 2.08%.

N.B. Repeated element analysis gave variable values for C.

#### ^Cy^L­(C_2_H_3_)­Pb: **6b**

The plumbylene was synthesized according to the general method using ^Cy^LPbBr **4b** (750 mg, 0.95 mmol). A colorless powder of **6b** (401 mg, 0.55 mmol, 57%) was isolated. Colorless crystals suitable for X-ray diffraction analysis were obtained from a concentrated diethyl ether solution, which was stored at 7 °C for 3 days.

^1^ **H NMR** (C_6_D_6_, 400 MHz, 298 K): δ = 0.79 (d, 3H, ^3^J_HH_ = 7.1 Hz, Si–Pr^i^–C*H*_3_), 0.99–1.09 (m, 4H_,_ Cy-*H*), 1.09–1.16 (m, 5H, Si–Pr^i^–C*H*_3_, Cy-*H*), 1.16–1.26 (m, 7H, Si–Pr^i^–C*H*_3_, Cy-*H*), 1.28 (d, 6H, ^3^J_HH_ = 7.0 Hz, Dipp–Pr^i^–C*H*_3_), 1.32 (d, 3H, ^3^J_HH_ = 7.0 Hz, Si–Pr^i^–C*H*_3_), 1.38–1.45 (m, 10H, Dipp–Pr^i^–C*H*_3_, Si–Pr^i^–C*H*_3_, Si–Pr^i^–C*H*), 1.50–1.69 (m, 4H, Cy-*H*), 1.70–1.79 (m, 4H, Cy-*H*), 1.79–1.88 (m, 2H, Ph_2_P–C*H*_2_), 1.90–1.95 (m, 1H, Cy-*H*), 2.05–2.15 (m, 1H, Cy-*H*), 3.56 (sept, 1H, ^3^J_HH_ = 6.9 Hz, Dipp–Pr^i^–C*H*), 4.17 (sept, 1H, ^3^J_HH_ = 6.8 Hz, Dipp–Pr^i^–C*H*), 6.03 (ddd, 1H, ^3^J_HH_ = 20.8 Hz, ^2^J_HH_ = 3.1 Hz, ^4^J_HP_ = 1.7 Hz, CH = C*H*_2_), 6.50 (dd, 1H, ^3^J_HH_ = 13.1 Hz, ^2^J_HH_ = 3.1 Hz, CH = C*H*_2_), 7.10–7.27 (m, 3H, Ar-*H*), 8.14 (dd, 1H, ^3^J_HH_ = 20.8 Hz, ^3^J_HH_ = 13.1 Hz, C*H*=CH_2_).

**^13^** **C­{** **^1^** **H} NMR** (C_6_D_6_, 101 MHz, 298 K) δ = 7.95 (d, ^1^J_CP_ = 12.3 Hz, Cy_2_P–*C*H_2_), 18.6, 19.1, 20.0, and 21.4 (Si–Pr^i^–*C*H_3_), 23.6, 24.3, and 26.4 (Dipp–Pr^i^–*C*H_3_), 26.8 (Dipp–Pr^i^–*C*H), 27.5 (Cy-*C*H_2_), 27.6 (d, J_CP_ = 2.7 Hz, Cy-*C*H_2_), 27.7 (d, J_CP_ = 5.0 Hz, Cy-*C*H_2_), 27.8 (d, J_CP_ = 3.2 Hz, Cy-*C*H_2_), 27.9 (Cy-*C*H_2_), 28.1 (Dipp–Pr^i^–*C*H), 28.5 (Dipp–Pr^i^–*C*H_3_), 30.4 (d, ^1^J_CP_ = 4.0 Hz, Cy-*C*H), 30.5 (Si–Pr^i^–*C*H), 30.9 (Si–Pr^i^–*C*H), 31.4 (d, J_CP_ = 3.6 Hz, Cy-*C*H_2_), 34.5 (d, ^1^J_CP_ = 5.0 Hz, Cy-*C*H), 38.6 (Cy-*C*H_2_), 122.1, 123.5, and 123.8 (Ar-*C*), 128.5 (CH = *C*H_2_), 222.5 (d, ^2^J_PC_ = 11.4 Hz, *C*H = CH_2_).

^31^ **P­{** ^1^ **H} NMR** (C_6_D_6_, 162 MHz, 298 K): δ = 30.4 (s, ^1^J_207PbP_ = 1039 Hz, *P*-Cy_2_).

**^29^** **Si­{** **^1^** **H} NMR** (C_6_D_6_, 99 MHz, 298 K): δ = 10.3 (d, ^2^J_SiP_ = 6.8 Hz, CH_2_–*Si*-^i^Pr_2_).

**MS/LIFDI-HRMS** found (calcd.) *m*/*z*: 709.3590 (709.3641) for [M-C_2_H_3_].

**Anal. Calcd** for C_33_H_58_NPPbSi: C, 53.92%; H, 7.95%; N, 1.91%; found C, 53.94%; H, 8.12%; N, 2.03%.

#### [^Ph^LPb:]­[BAr^F^_4_], **7**

To a solid mixture of vinyl-plumylene **6a** (450 mg, 0.62 mmol, 1.0 equiv) and [Ph_3_C]­[BAr^F^_4_] (689 mg, 0.62 mmol, 1.0 equiv) precooled to −80 °C was added toluene (40 mL) with rapid stirring. After stirring for 30 min at this temperature, the mixture was allowed to warm to room temperature, the color gradually turning orange. After 4 h of further stirring, the mixture appeared as a dark orange suspension with red oil on the walls of the flask, and a yellow supernatant solution. The solution was separated from the oil, and the latter was dried *in vacuo*. Crystallization from a PhF solution (3 mL) layered with pentane (15 mL) yielded **7** as dark orange crystals (598 mg, 0.38 mmol, 62%), which were suitable for X-ray diffraction analysis.

N. B. The NMR spectra contain two forms of **7**, hypothesized to be the ‘free’ cation, and the adduct with the BAr^F^_4_ counteranion.

**^1^** **H NMR** (CD_2_Cl_2_, 400 MHz, 298 K): δ = 0.41 (sept, 2H, ^3^J_HH_ = 7.2 Hz, Si–Pr^i^–C*H*), 0.62 (d, 6H, ^3^J_HH_ = 7.3 Hz, Si–Pr^i^–C*H*_3_), 0.72 (d, 6H, ^3^J_HH_ = 7.3 Hz, Si–Pr^i^–C*H*_3_), 0.80–0.91 (m, 18H, Si–Pr^i^–C*H*_3_), 0.99–1.08 (m, 14H, Si–Pr^i^–C*H*_3_), 1.08–1.14 (m, 24H, Dipp–Pr^i^–C*H*_3_), 1.15–1.17 (m, 2H, Si–Pr^i^–C*H*), 1.23–1.29 (m, 12H, ^3^J_HH_ = 6.8 Hz, Dipp–Pr^i^–C*H*_3_), 1.30–1.33 (m, 2H, Si–Pr^i^–C*H*), 2.57 (d, 2H, ^2^J_HP_ = 19.0 Hz, Ph_2_P–C*H*_2_), 2.87 (d, 4H, ^2^J_HP_ = 19.0 Hz, Ph_2_P–C*H*_2_), 3.13 (sept, 2H, Dipp–Pr^i^–C*H*_3_), 3.17 (sept, 2H, Dipp–Pr^i^–C*H*_3_), 7.00–7.10 (m, 6H, Ar-*H*), 7.10–7.18 (m, 6H, Ar-*H*), 7.25–7.40 (m, 9H, Ar-*H*), 7.44–7.50 (m, 6H, Ar-*H*), 7.57 (s, 12H, Ar_BArF_–H_para_), 7.57–7.65 (m, 8, Ar-*H*), 7.65–7.74 (m, 4H, Ar-*H*), 7.74 (s, 24H, Ar_BArF_–H_ortho_).

**^13^** **C­{** **^1^** **H} NMR** (CD_2_Cl_2_, 101 MHz, 298 K) δ = 7.24 and 11.6 (Ph_2_P–*C*H_2_), 14.0 (Si–Pr^i^–*C*H), 14.2 (Si–Pr^i^–*C*H_3_), 16.8 (Si–Pr^i^–*C*H), 17.4, 17.9, 18.1, and 19.3 (Si–Pr^i^–*C*H_3_), 22.6 (Dipp–Pr^i^–*C*H_3_), 22.8 (Si–Pr^i^–*C*H_3_), 23.7 and 28.0 (Dipp–Pr^i^–*C*H_3_), 29.0 (Dipp–Pr^i^–*C*H), 31.5 (Dipp–Pr^i^–*C*H_3_), 34.6 (Si–Pr^i^–*C*H), 117.9 (m, Ar-*C*), 123.7, 124.1, 124.1, and 126.4 (Ar-*C*), 129.2 (m, Ar-*C*), 129.5 (m, Ar-*C*), 130.3 and 130.4 (Ar-*C*), 131.0 (d, J_CP_ = 10.6 Hz, Ar-*C*) 132.1 (Ar-*C*), 133.2 (d, J_CP_ = 12.1 Hz, Ar-*C*), 133.7 (Ar-*C*), 134.5 (d, J_CP_ = 8.1 Hz, Ar-*C*), 135.2 and 135.9 (Ar-*C*), 161.5, 161.9, 162.4, and 162.9 (Ar-*C*_BArF_).

^31^ **P­{** **^1^** **H} NMR** (CD_2_Cl_2_, 162 MHz, 298 K): δ = 33.5 (s, adducted cation *P*-Ph_2_), 139.0 (s, ‘free’ cation, *P*-Ph_2_).

**^29^** **Si­{** **^1^** **H} NMR** (CD_2_Cl_2_, 99 MHz, 298 K): δ = 2.96 (s, CH_2_–*Si*-^i^Pr_2_), 3.10 (s, CH_2_–*Si*-^i^Pr_2_).

**^19^** **F­{** **^1^** **H}** NMR (CD_2_Cl_2_, 376 MHz, 298 K): δ­(ppm) = −62.89 (s, BAr^F^-C*F*_3_), −62.84 (s, BAr^F^-C*F*_3_).

**MS/LIFDI-HRMS** found (calcd.) *m*/*z*: 696.2620 (696.2663) for [M-BAr^F^_4_]^+^.

**Anal. Calcd** for C_63_H_67_BF_24_NPPbSi: C, 48.53%; H, 3.56%; N, 0.90%; found C, 52.13%; H, 4.13%; N, 0.95%.

N.B. Repeated element analysis gave variable but consistently high values for C, potentially due to the presence of free BAr^F^_4_ anion, which is difficult to fully quantify.

#### [^Ph^L­(C_2_H_3_)­Ge:]·Ni­(PPh_3_)_2_, **8**

To a solid mixture of vinyl-germylene **4a** (300 mg, 0.51 mmol, 1.0 equiv) and (cod)­Ni­(PPh_3_)_2_ (353 mg, 0.51 mmol, 1.0 equiv) was added at toluene (30 mL) at −80 °C. While stirring at this temperature for 30 min, the color changed gradually to dark red. The mixture was then warmed to ambient temperature with stirring over the course of 1 h, leading to a red suspension. All volatiles were subsequently removed *in vacuo*, and the remaining red solid was washed with pentane (10 mL), yielding **8** as an analytically pure dark red powder (328 mg, 0.28 mmol, 55%). Crystals suitable for X-ray diffraction analysis were obtained from a concentrated hexane solution, which was stored at 7 °C for 7 days.

**^1^** **H NMR** (C_6_D_6_, 400 MHz, 298 K): δ = 0.67 (d, 6H, ^3^J_HH_ = 7.3 Hz, Si–Pr^i^–C*H*_3_), 0.78–0.90 (m, 6H, Si–Pr^i^–C*H*_3_), 1.05 (d, 6H, ^3^J_HH_ = 6.6 Hz, Dipp–Pr^i^–C*H*_3_), 1.41 (d, 6H, ^3^J_HH_ = 6.6 Hz, Dipp–Pr^i^–C*H*_3_), 2.43 (d, 2H, ^3^J_HH_ = 8.9 Hz, P–C*H*_2_), 3.92 (sept, ^3^J_HH_ = 7.3 Hz, Dipp–Pr^i^–C*H*), 5.86–5.94 (m, 2H, CH = C*H*_2_), 6.48–6.54 (m, 1H, C*H*=CH_2_), 6.78–6.86 (m, 3H, Ar–C*H*), 6.90–6.99 (m, 20H, Ar–C*H*), 7.12–7.20 (m, 7H, Ar–C*H*), 7.30–7.46 (m, 13H, Ar–C*H*), 7.70–7.76 (br, 1H, Ar–C*H*).

**^13^** **C­{** **^1^** **H} NMR** (C_6_D_6_, 101 MHz, 298 K) δ = 14.3 and 18.3 (Si–Pr^i^–*C*H_3_), 18.6, (Si–Pr^i^–*C*H), 19.8 (Ph_2_P–*C*H_2_), 24.7 and 26.5 (Dipp–Pr^i^–*C*H_3_), 28.1 (Dipp–Pr^i^–*C*H),124.5 and 125.0 (Ar-*C*), 127.5 (m, Ar-*C*), 128.8 (Ar-*C*), 132.4 (CH = *C*H_2_), 133.9 (Ar-*C*) 134.4 (d, J_PC_ = 14.3 Hz), 162.4 (*C*H = CH_2_).

**^31^** **P­{** **^1^** **H} NMR** (C_6_D_6_, 162 MHz, 298 K): δ = 9.3 (t, ^2^J_PP_ = 15.7 Hz, Ph_2_ *P*–CH_2_), 42.0 (d, ^2^J_PP_ = 13.7 Hz, Ph_3_ *P*-Ni).

**^29^** **Si­{** **^1^** **H} NMR** (C_6_D_6_, 99 MHz, 298 K): δ = 6.7 (s, CH_2_–*Si*-^i^Pr_2_).

**λ** **_max_**, nm (ε, L mol^–1^ cm^–1^): 362 (10140) and 440 (4520).

**MS/LIFDI-HRMS** found (calcd.) *m*/*z*: 909.2556 (909.2614) for [M-PPh_3_].

**Anal. Calcd** for C_69_H_76_GeNNiP_3_Si: C, 70.73%; H, 6.54%; N, 1.20%; found C, 72.53%; H, 6.72%; N, 1.30%.

N.B. Repeated element analysis gave variable but consistently high values for C and H, possibly due to incomplete removal of solvent of crystallization despite extended drying times for those samples.

#### [^Cy^L­(C_2_H_3_)­Ge]_2_Ni, **9**

To a solid mixture of **4b** (350 mg, 0.58 mmol, 2.0 equiv) and Ni­(cod)_2_ (80.2 mg, 0.29 mmol, 1.0 equiv) was added toluene (40 mL) at −80 °C with rapid stirring. After 30 min at this temperature, the mixture was allowed to warm to ambient temperature, leading to a gradual color change to purple. After stirring for a further 1 h, all volatiles were removed *in vacuo*, and the dark purple residue extracted with pentane (50 mL). The extract was filtered, concentrated to 20 mL, and stored at −35 °C for 5 days, leading to the formation of dark purple crystals of compound **9** (189 mg, 0.15 mmol, 51%), which were suitable for X-ray diffraction analysis.

**^1^** **H NMR** (C_6_D_6_, 400 MHz, 298 K): δ = 0.70 (d, 6H, ^3^J_HH_ = 7.2 Hz, Si–Pr^i^–C*H*_3_), 1.10–2.26 (m, 34H, Si–Pr^i^–C*H*_3_, Si–Pr^i^–C*H*, Dipp–Pr^i^–C*H*_3_, Cy–C*H*_2_), 1.28–1.52 (m, 26H, Si–Pr^i^–C*H*_3_, Si–Pr^i^–C*H*, Dipp–Pr^i^–C*H*_3_, Cy–C*H*_2_), 1.55–1.75 (m, 20H, Cy–C*H*_2_, P–C*H*_2_), 1.83–2.00 (m, 6H, Cy–C*H*_2_), 2.01–2.09 (m, 2H, Cy–C*H*_2_), 2.25–2.35 (m, 4H, Cy–C*H*), 3.66 (sept, 4H, Dipp–Pr^i^–C*H*), 3.74 (sept, 4H, Dipp–Pr^i^–C*H*), 6.04 (dd, 2H, ^3^J_HH_ = 12.5 Hz, ^3^J_HH_ = 3.4 Hz, CH = C*H*_2_), 6.18 (dd, 2H, ^3^J_HH_ = 20.3 Hz, ^2^J_HH_ = 3.4 Hz, CH = C*H*_2_), 6.43 (dd, 2H, ^3^J_HH_ = 20.3 Hz, ^3^J_HH_ = 12.5 Hz, C*H*=CH_2_), 7.10–7.24 (m, 6H, Ar–C*H*).

**^13^** **C­{** **^1^** **H} NMR** (C_6_D_6_, 101 MHz, 298 K) δ = 6.8 (s, Cy_2_P–*C*H_2_), 14.5 and 14.8 (Si–Pr^i^–*C*H), 18.6, 19.9, 20.2, 24.4, 24.5, and 26.0 (Si–Pr^i^–*C*H_3_/Dipp–Pr^i^–*C*H_3_), 26.4 (Dipp–Pr^i^–*C*H), 27.0 (Si–Pr^i^–*C*H_3_/Dipp–Pr^i^–*C*H_3_), 27.3 (Dipp–Pr^i^–*C*H), 27.4 (Si–Pr^i^–*C*H_3_/Dipp–Pr^i^–*C*H_3_), 27.5 (m, Cy-*C*H_2_), 27.9 (m, Cy-*C*H_2_), 27.9 (m, Cy-*C*H_2_), 28.4 (Si–Pr^i^–*C*H_3_/Dipp–Pr^i^–*C*H_3_), 28.6 (m, Cy-*C*H_2_), 28.8 (m, Cy-*C*H_2_), 29.6, 29.9, and 30.8 (Cy-*C*H), 41.4 (Cy-*C*H_2_), 42.4 (m, Cy-*C*H_2_), 124.2, 124.6, and 124.9 (Ar-*C*), 134.1 (CH = *C*H_2_), 156.5 (m, *C*H = CH_2_).

**^31^** **P­{** **^1^** **H} NMR** (C_6_D_6_, 162 MHz, 298 K): δ = 35.4 (s, *P*-Cy_2_).

^29^ **Si­{** ^1^ **H} NMR** (C_6_D_6_, 99 MHz, 298 K): δ = 5.5 (s, CH_2_–*Si*-^i^Pr_2_).

**MS/LIFDI-HRMS** found (calcd.) *m*/*z*: 1258.5885 (1258.5938) for [M].

**λ** **_max_**, nm (ε, L·mol^–1^·cm^–1^): 406 (6500) and 519 (7720).

**Anal. Calcd** for C_66_H_116_Ge_2_N_2_NiP_2_Si_2_: C, 62.93%; H, 9.28%; N, 2.22%; found C, 62.38%; H, 9.30%; N, 2.22%.

### X-ray Crystallographic Details

Single crystals of **4a**, **5b**, **6b**, **7**, **8**, and **9** suitable for X-ray structural analysis were mounted in perfluoroalkyl ether oil on a nylon loop and positioned in a 150 K cold N_2_ gas stream. Data collection was performed with a STOE StadiVari diffractometer (MoKα radiation) equipped with a DECTRIS PILATUS 300 K detector. Structures were solved using SHELXT-16,^81^ and refined by full-matrix least-squares calculations against F2 (SHELXL-2018).^82^ The positions of the hydrogen atoms were calculated and refined using a riding model. All non-hydrogen atoms were treated with anisotropic displacement parameters. Crystal data, details of data collections, and refinements for all structures can be found in their CIF files, which are available free of charge via www.ccdc.cam.ac.uk/data_request/cif. Details of crystallographic details are summarized in Tables S1 and S2.

## Computational Methods and Details

Computational experiments were performed using the ORCA 6.0.1 program.^83^ Geometry optimization was carried out at the ωB97XD level with the def2-TZVPP basis set for Ge, Ni, and P, and the def2-SVP basis set for all other atoms.^84–87^ Stationary points were confirmed as true minima by vibrational frequency analysis (no negative eigenvalues). Bond indices (Wiberg Bond Index, Mayer Bond Order) and NPA charges were determined using the NBO 7.0 program implemented in ORCA 6.0.1, using optimized geometries from above.^88^ Dative interactions were determined through analysis of the NBO output, and visualized in ChemCraft through combination of the associated NLMOs.^89^

## Supplementary Material



## Footnotes

* The crystallographic data for **4a** suggest disorder in the [C_2_H_3_] unit of this species, potentially affecting the accuracy of the given C=C bond length. Due to only weak residual electron density, this disorder could not be modeled.

## Notes

Experimental procedures for all new compounds not described above; printed spectra for all new compounds; details regarding X-ray crystallography; further computational details (PDF)
