# Supplementary material for: Crystalline Half-Parent Vinyl-tetrylenes: Synthesis and Coordination Chemistry
Source: Inorg Chem. 2025 Dec 5;64(50):24898–909. doi: 10.1021/acs.inorgchem.5c04745 (PMC12728937; doi:10.1021/acs.inorgchem.5c04745)
Supplement: Supplementary file 1 [file ic5c04745_si_001.pdf]

Supporting Information

Crystalline half-parent vinyl-tetrylenes: synthesis and coordination chemistry

Annika Schulz,[a] and Terrance. J. Hadlington*,[a]

[a] Fakultät für Chemie, School of Natural Sciences, TU München, Lichtenberg Strasse 4, 85749 Garching, Germany

Email: terrance.hadlington@tum.de

1. Experimental methods and data……………………..S2

General Considerations……………………………………S2

Synthetic details and data……………………………..…S2

NMR, MS, UV/vis, and IR spectra…………………………....S6

2. X-ray crystallographic details…………………….….S43

3. Computational methods and details………………..…S47

4. References…………………………………………....S55

General considerations.

All experiments and manipulations were carried out under dry oxygen-free argon atmosphere using standard Schlenk techniques or in a MBraun inert atmosphere glovebox containing an atmosphere of high-purity argon. C_6D_6 was dried, degassed and stored over a potassium mirror. THF and Et_2O were dried over Na/Benzephenone, distilled and stored over 4Å molecular sieves. All other solvents were dried over activated 4 Å molecular sieves and thoroughly degassed before use. ^{Ph}LK,[1] ^{Cy}LK,[2] **1a** ^{Ph}LGeCl,[1] **2a** ^{Ph}LSnCl,[3] Ni(cod)$_2$,[4] and (cod)Ni(PPh$_3$)$_2$ [5] were synthesised using reported procedures. All other reagents were used as received. NMR spectra were recorded on a Bruker AV 400 Spectrometer. 1H and ^{13}C{1H} NMR spectra were referenced to the residual solvent signals as internal standards. ^{29}Si{^{1}H} NMR spectra were externally calibrated with $SiMe_4$. ^{31}P{^{1}H} NMR spectra were externally calibrated with H_3PO_4. Liquid Injection Field Desorption Ionization Mass Spectrometry (LIFDI-MS) was measured directly from an inert atmosphere glovebox with a Thermo Fisher Scientific Exactive Plus Orbitrap equipped with an ion-source from Linden CMS.8 Commercial hydrogen gas with a purity of ≥99.999% was used in all hydrogenation experiments. Elemental analyses (C, H, N) were performed with a combustion analyzer (elementar vario EL, Bruker). Absorption spectra (UV/vis) were recorded on an Agilent Cary 60 UV/vis spectrophotometer.

^{Cy}L(Cl)Ge:, 1b.

To a solution of $GeCl_2$·dioxane (2.0 g, 8.63 mmol, 1.0 eq.) in THF (40 mL), a solution of ^{Cy}LK (4.66 g, 8.63 mmol, 1.0 eq.) in THF (100 mL) was added dropwise at -80°C. The reaction was stirred at this temperature for 1 h, and subsequently allowed to warm to room temperature, and stirred for an additional 2 h, resulting in a pale-yellow solution above a colourless precipitate. All volatiles were removed *in vacuo*, and the residue was extracted with toluene (100 mL). The extract was filtered, all volatiles removed *in vacuo*, and the remaining solid was washed with pentane (2 x 10 mL). The off-white powder was dried *in vacuo*, yielding **2b** as a colourless powder (4.25 g, 6.98 mmol, 81%).

^{1}H NMR (C_6D_6, 400 MHz, 298 K): δ = 0.67 (d, 3H, $^3J_{HH}$ = 7.1 Hz, Si-Pr^i-C*H$_3$*), 1.01 (d, 3H, $^3J_{HH}$ = 7.1 Hz, Si-Pr^i-C*H$_3$*), 1.00-1.09 (m, 4H, Cy-C*H*, Si-Pr^i-C*H*), 1.11 (d, 3H, $^3J_{HH}$ = 7.2 Hz, Si-Pr^i-C*H$_3$*), 1.15-1.23 (m, 4H, Cy-C*H*), 1.28 (d, 3H, $^3J_{HH}$ = 6.8 Hz, Dipp-Pr^i-C*H$_3$*), 1.35 (d, 3H, $^3J_{HH}$ = 6.8 Hz, Dipp-Pr^i-C*H$_3$*), 1.43 (d, 3H, $^3J_{HH}$ = 7.0 Hz, Si-Pr^i-C*H$_3$*),), 1.46 (d, 3H, $^3J_{HH}$ = 6.6 Hz, Dipp-Pr^i-C*H$_3$*), 1.50-1.56 (m, 5H, Cy-C*H*, Ph$_2$P-C*H$_2$*, Si-Pr^i-C*H*), 1.57 (d, 3H, $^3J_{HH}$ = 6.6 Hz, Dipp-Pr^i-C*H$_3$*), 1.61-1.68 (m, 8H, Cy-C*H*), 1.88-1.92 (m, 2H, Cy-C*H*), 1.99-2.03 (m, 1H, Cy-C*H*), 3.41 (sept, 1H, $^3J_{HH}$ = 6.6 Hz, Dipp-Pr^i-C*H*), 4.21 (sept, 1H, $^3J_{HH}$ = 6.6 Hz, Dipp-Pr^i-C*H*), 7.12-7.16 (m, 2H, Ar-C*H*), 7.21-7.23 (m, 1H, Ar-C*H*).

$^{13}C\{^1H\}$ NMR (C_6D_6, 101 MHz, 298 K) δ = -2.0 (d, $^1J_{CP}$ = 4.0 Hz, Cy_2P-CH_2), 14.6 (d, J_{PC} = 2.9 Hz, Cy-CH_2), 17.3 (d, J_{PC} = 4.0 Hz, Cy-CH_2), 17.8, 19.9, 20.0 and 20.4 (Si-Pr^i-CH_3), 23.0 and 23.6 (Dipp-Pr^i-CH_3), 26.1 and 26.2 (Si-Pr^i-*C*H), 27.5 (Cy-CH_2), 27.6 (Dipp-Pr^i-*C*H), 27.7 (Dipp-Pr^i-CH_3), 27.9 and 28.0 (Cy-CH_2), 28.7 (Dipp-Pr^i-*C*H), 28.7 (d, J_{PC} = 6.2 Hz, Cy-CH_2), 28.9 (d, J_{PC} = 7.3 Hz, Cy-CH_2), 30.0 (Dipp-Pr^i-CH_3), 30.0 (d, J_{PC} = 2.6 Hz, Cy-*C*H), 30.6 (d, J_{PC} = 2.6 Hz, Cy-*C*H), 34.2 (d, J_{PC} = 9.2 Hz, Cy-CH_2), 35.7 and 35.8 (Cy-CH_2), 123.8, 124.3 and 125.0 (Ar-*C*), 143.0 (d, J_{PC} = 11.7 Hz, Ar-*C*), 146.6 (Ar-*C*), 149.7 (d, J_{PC} = 1.8 Hz, Ar-*C*).

$^{31}P\{^1H\}$ NMR (C_6D_6, 162 MHz, 298 K): δ = 17.1 (s, *P*-Cy_2).

$^{29}Si\{^1H\}$ NMR (C_6D_6, 99 MHz, 298 K): δ = 18.2 (d, $^2J_{SiP}$ = 9.3 Hz, CH_2-*Si*-$^iPr_2$).

MS/LIFDI-HRMS found (calcd.) m/z: 609.2751 (609.2742) for [M].

Anal. calcd. for C31H55ClGeNPSi: C, 61.15%; H, 9.10%; N, 2.30%. **found**: C, 60.98%; H, 8.94%; N, 2.28%.

^{Cy}L(Cl)Sn:, 2b.

To a suspension of $SnCl_2$ (1.40 g, 7.41 mmol, 1.0 eq.) in THF (10 mL), a solution of ^{Cy}LK (4.00 g, 7.41 mmol, 1.0 eq.) in THF (100 mL) was added dropwise at -80°C. After stirring for 1 h at this temperature, the mixture was allowed to warm to room temperature and was stirred for an additional 2 h, resulting in a yellow solution over a white-grey precipitate. All volatiles were removed *in vacuo*, and the residue was extracted with toluene (80 mL), filtered and all volatiles removed *in vacuo*. The remaining solid was washed with pentane (10 mL) and dried *in vacuo*, yielding **2b** as an off-white powder (3.30 g, 5.04 mmol, 68%).

^1H NMR (C_6D_6, 400 MHz, 298 K): δ = 0.72 (d, 3H, $^3J_{HH}$ = 6.8 Hz, Si-Pr^i-CH_3),0.88-1.19 (m, 14H, Cy-C*H* / Si-Pr^i-C*H*), 1.19-1.24 (m, 6H, Dipp-Pr^i-CH_3/ Si-Pr^i-CH_3), 1.36 (d, 3H, $^3J_{HH}$ = 6.9 Hz, Dipp-Pr^i-CH_3), 1.44 (dd, 6H, $^3J_{HH}$ = 6.8 Hz, J = 2.4 Hz, Dipp-Pr^i-CH_3), 1.47-1.58 (m, 7H, Si-Pr^i-CH_3 / Si-Pr^i-C*H*), 1.58-1.67 (m, 6H, Cy-C*H* / Cy_2P-CH_2), 1.67-1.93 (m, 5H Cy-C*H*), 2.31 (m, 1H, Cy-C*H*), 3.48 (hept, 1H, $^3J_{HH}$ = 6.9 Hz, Dipp-Pr^i-C*H*), 4.31 (hept, 1H, $^3J_{HH}$ = 6.9 Hz, Dipp-Pr^i-C*H*), 7.09-7.13 (m, 2H, Ar-C*H*), 7.21-7.23 (m, 1H, Ar-C*H*).

$^{13}C\{^1H\}$ NMR (C_6D_6, 101 MHz, 298 K): δ = 0.1 (d, $^1J_{PC}$ = 6.3 Hz, Cy_2P-CH_2), 15.8 (s, J_{PC} = 2.2 Hz, Si-Pr^i-*C*H), 17.9 (d, J_{PC} = 3.7 Hz, Si-Pr^i-*C*H), 18.1 Si-Pr^i-CH_3), 20.0 (s, Si-Pr^i-CH_3), 20.1 (s, Si-Pr^i-CH_3), 20.6 (s, Si-Pr^i-CH_3), 23.1 (Dipp-Pr^i-CH_3), 23.7 (s, Dipp-Pr^i-CH_3), 26.2 (d, J_{PC} = 6.6 Hz, Cy-*C*), 27.3 (Dipp-Pr^i-*C*H), 27.4, 27.5, 27.5, 27.5, 27.6, 27.8, 27.9 (Cy-*C*), 28.3 (Dipp-Pr^i-CH_3), 28.7 (Dipp-Pr^i-CH_3), 28.8 (Dipp-Pr^i-CH_3), 29.3 (d, J_{PC} = 4.0 Hz, Cy-*C*H), 30.1 (s, Si-Pr^i-CH_3), 30.6 (s, Cy-*C*), 31.2 (d, J_{PC} = 3.4 Hz, Cy-*C*), 34.2 (d, J_{PC} = 6.3 Hz, Cy-*C*), 35.2 (d, J_{PC} = 12.6 Hz, Cy-*C*), 123.7 (Ar-*C*), 124.0 (Ar-*C*), 124.2 (Ar-*C*), 144.9 (d, J_{PC} = 7.2 Hz, Ar-*C*), 147.6 (Ar-*C*), 148.7 (Ar-*C*).

^{31}P{^{1}H} NMR (C_6D_6, 162 MHz, 298 K): δ = 18.8 (s, $^{1}J_{117SnP}$ = 1480 Hz, $^{1}J_{119SnP}$ = 1550 Hz, *P*-Cy_2).

^{29}Si{^{1}H} NMR (C_6D_6, 99 MHz, 298 K): δ = 13.8 (d, $^{2}J_{SiP}$ = 6.3 Hz, CH_2-*Si*-$^{i}Pr_2$).

MS/LIFDI-HRMS found (calcd.) m/z: 655.2596 (655.2550) for $[M]^+$.

Anal. calcd. for $C_{31}H_{55}ClNPSiSn$: C, 56.85%; H, 8.46%; N, 2.14%. **found**: C, 56.52 %; H, 7.93 %; N, 2.12 %.

^{Ph}L(Br)Pb:, 3a.

To a suspension of $PbBr_2$ (2.29 g, 6.25 mmol, 1.1 eq.) in THF (40 mL) a solution of ^{Ph}LK (3.00 g, 5.68 mmol, 1.0 eq.) in THF (80 mL) was added dropwise at -80°C. The mixture was allowed to slowly warm to room temperature under stirring overnight, resulting in an orange solution over a grey suspension. All volatiles were subsequently removed *in vacuo*, and the residue was extracted with toluene (100 mL) and filtered. All volatiles were removed *in vacuo* from the filtrate, and the solid was washed with *n*-hexane (15 mL) and dried, yielding **3a** as an off-white powder (2.65 g, 3.42 mmol, 60%). Crystals suitable for X-ray diffraction analysis were obtained from a concentrated *n*-hexane solution at -35°C after 2 days.

^{1}H NMR (THF-*d*^{8}, 400 MHz, 298 K): δ = 0.80 (d, 6H, $^{3}J_{HH}$ = 7.2 Hz, Si-Pr^{i}-C*H*$_3$), 1.00 (d, 14H, $^{3}J_{HH}$ = 6.8 Hz, Dipp-Pr^{i}-C*H*$_3$, Si-Pr^{i}-C*H*), 1.27 (d, 6H, $^{3}J_{HH}$ = 7.5 Hz, Si-Pr^{i}-C*H*$_3$), 2.77 (d, 2H, $^{2}J_{HP}$ = 9.0 Hz, Ph_2P-C*H*$_2$), 3.50-3.54 (br sept, 2H, Dipp-Pr^{i}-C*H*), 6.81 (t, 1H, $^{3}J_{HH}$ = 7.6 Hz, Ar-C*H*), 6.96 (d, 2H, $^{3}J_{HH}$ = 7.6 Hz, Ar-C*H*), 7.45-7.55 (m, 6H, Ar-C*H*), 7.80-7.86 (m, 4H, Ar-C*H*).

^{13}C{^{1}H} NMR (THF-*d*^{8}, 101 MHz, 298 K) δ = 13.6 (d, $^{1}J_{CP}$ = 5.1 Hz, Ph_2P-*C*H_2), 18.6 and 19.7 (Si-Pr^{i}-*C*H), 21.3 (Si-Pr^{i}-*C*H_3), 23.6 (Dipp-Pr^{i}-*C*H_3), 27.5 (Dipp-Pr^{i}-*C*H), 27.7 (Dipp-Pr^{i}-*C*H_3), 123.5 and 124.0 (Ar-*C*), 129.8 (d, J_{CP} = 9.5 Hz, Ar-*C*), 131.8 (Ar-*C*), 134.3 (d, J_{CP} = 12.1 Hz, Ar-*C*).

^{31}P{^{1}H} NMR (THF-*d*^{8}, 162 MHz, 298 K): δ = 43.9 (s, *P*-Ph_2, $^{1}J_{207PbP}$ = 1087 Hz).

^{29}Si{^{1}H} NMR (THF-*d*^{8}, 99 MHz, 298 K): δ = 13.3 (d, $^{2}J_{SiP}$ = 5.4 Hz, CH_2-*Si*-$^{i}Pr_2$).

MS/LIFDI-HRMS found (calcd.) m/z: 696.2687 (696.2669) for [M-Br].

Anal. calcd. for $C_{31}H_{43}BrNPPbSi$: C, 47.99%; H, 5.59%; N, 1.81%; found C, 46.47%; H, 5.40%; N, 1.79%.

^{Cy}L(Br)Pb:, 3b.

To a suspension of $PbBr_2$ (1.87g, 5.09 mmol, 1.1 eq.) in THF (15 mL), a solution of ^{Cy}LK (2.50 g, 4.63 mmol, 1.0 eq.) in THF (80 mL) was added dropwise at -80°C. The mixture was allowed to slowly warm to room temperature under stirring overnight,

resulting in an orange solution over a greyish precipitate. All volatiles were removed *in vacuo*, the residue was extracted with toluene (120 mL), and filtered. After the solvent was removed *in vacuo* from the filtrate, the solid was washed with *n*-hexane (20 mL) and dried *in vacuo*, yielding **3b** as an off-white powder (1.98 g, 2.51 mmol, 54%).

^{1}H NMR (THF-*d*^{8}, 400 MHz, 298 K): δ = 1.06 (d, 3H, $^{3}J_{HH}$ = 6.7 Hz, Si-Pr^{i}-C*H₃*), 1.12 (d, 7H, $^{3}J_{HH}$ = 6.8 Hz, Si-Pr^{i}-CH_3, Si-Pr^{i}-C*H*), 1.17 (d, 4H, $^{3}J_{HH}$ = 6.8 Hz, Si-Pr^{i}-CH_3, Si-Pr^{i}-C*H*), 1.25 (d, 12H, $^{3}J_{HH}$ = 7.0 Hz, Dipp-Pr^{i}-*C*H_3), 1.36-1.44 (m, 8H, Cy−C*H*), 1.55-1.65 (m, 4H, Cy−C*H*), 1.77-1.79 (m, 2H, Cy−C*H*), 1.90-1.96 (m, 6H, Cy−C*H*, Ph_2P-CH_2), 2.04-2-10 (m, 4H, Cy−C*H*), 3.53 (sept, 2H, $^{3}J_{HH}$ = 6.9 Hz, Dipp-Pr^{i}-C*H*), 6.85 (t, 1H, $^{3}J_{HH}$ = 7.6 Hz, Ar-C*H*), 7.03 (d, 1H, $^{3}J_{HH}$ = 7.6 Hz, Ar-C*H*).

^{13}C{^{1}H} NMR (THF-*d*^{8}, 101 MHz, 298 K) δ = 7.84 (d, $^{1}J_{CP}$ = 8.4 Hz, Cy_2P-*C*H_2), 15.4 (d, J_{CP} = 2.2 Hz, Cy−*C*), 15.4 (d, J_{CP} = 2.2 Hz, Cy−*C*), 18.9 (d, J_{CP} = 2.2 Hz, Cy−*C*), 19.0 (d, J_{CP} = 2.6 Hz, Cy−*C*), 21.3 (Si-Pr^{i}-*C*H), 23.9 and 24.3 (Si-Pr^{i}-*C*H_3), 27.1 (Dipp-Pr^{i}-*C*H), 27.7 (Cy−*C*), 28.4 (Dipp-Pr^{i}-*C*H_3), 28.5 (Dipp-Pr^{i}-*C*H), 28.6 (Cy−*C*), 28.7 (Cy−*C*), 29.0 (d, J_{CP} = 1.8 Hz, Cy−*C*) 30.5 and 30.6 (Cy−*C*), 31.6 (Dipp-Pr^{i}-*C*H_3), 36.7 and 36.9 (Ar-*C*), 123.4, 123.7, 124.1 and 124.2 (Ar-*C*).

^{31}P{^{1}H} NMR (THF-*d*^{8}, 162 MHz, 298 K): δ = 70.2 (s, *P*-Cy_2, $^{1}J_{207PbP}$ = 1199 Hz).

^{29}Si{^{1}H} NMR (THF-*d*^{8}, 99 MHz, 298 K): δ = 3.8 (d, $^{2}J_{SiP}$ = 16.1 Hz, CH_2-*Si*-$^{i}Pr_2$).

MS/LIFDI-HRMS found (calcd.) m/z: 708.3617 (708.3608) for [M-Br].

Anal. calcd. for $C_{31}H_{55}$BrNPPbSi: C, 47.25%; H, 7.04%; N, 1.78%; found C, 47.92%; H, 7.14%; N, 1.92%.

Experimental Spectra etc.

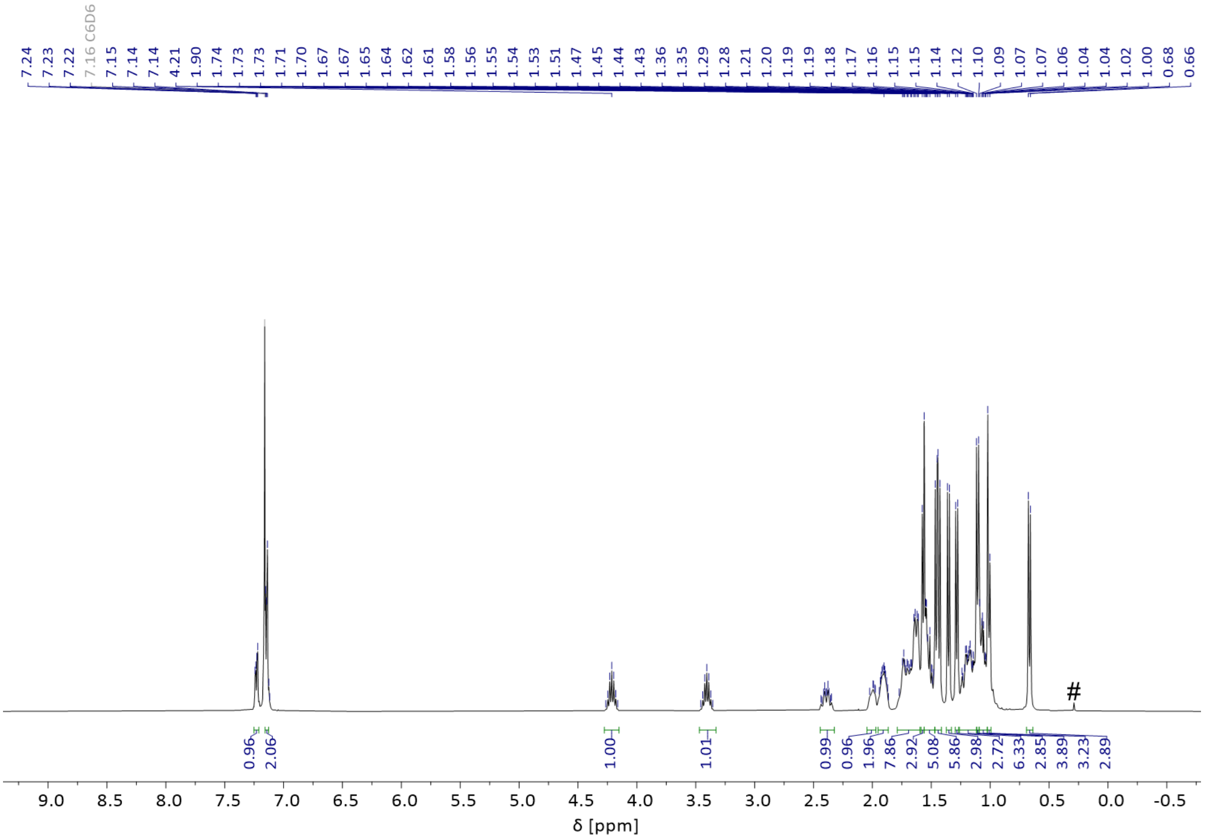

Figure S1. ^{1}H NMR spectrum of **1b** as a solution in C_6D_6 at ambient temperature; # marks an unknown impurity.

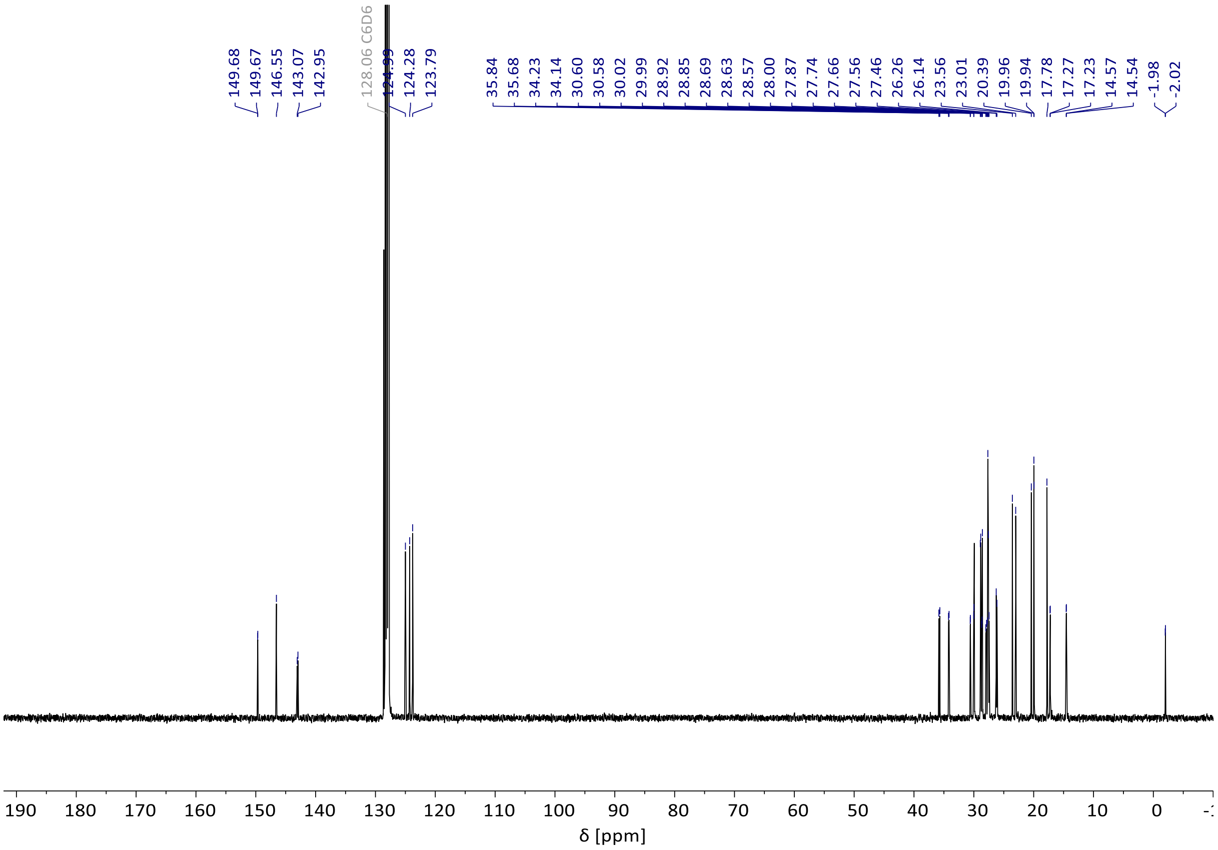

Figure S2. $^{13}C\{^{1}H\}$ NMR spectrum of **1b** as a solution in C_6D_6 at ambient temperature..

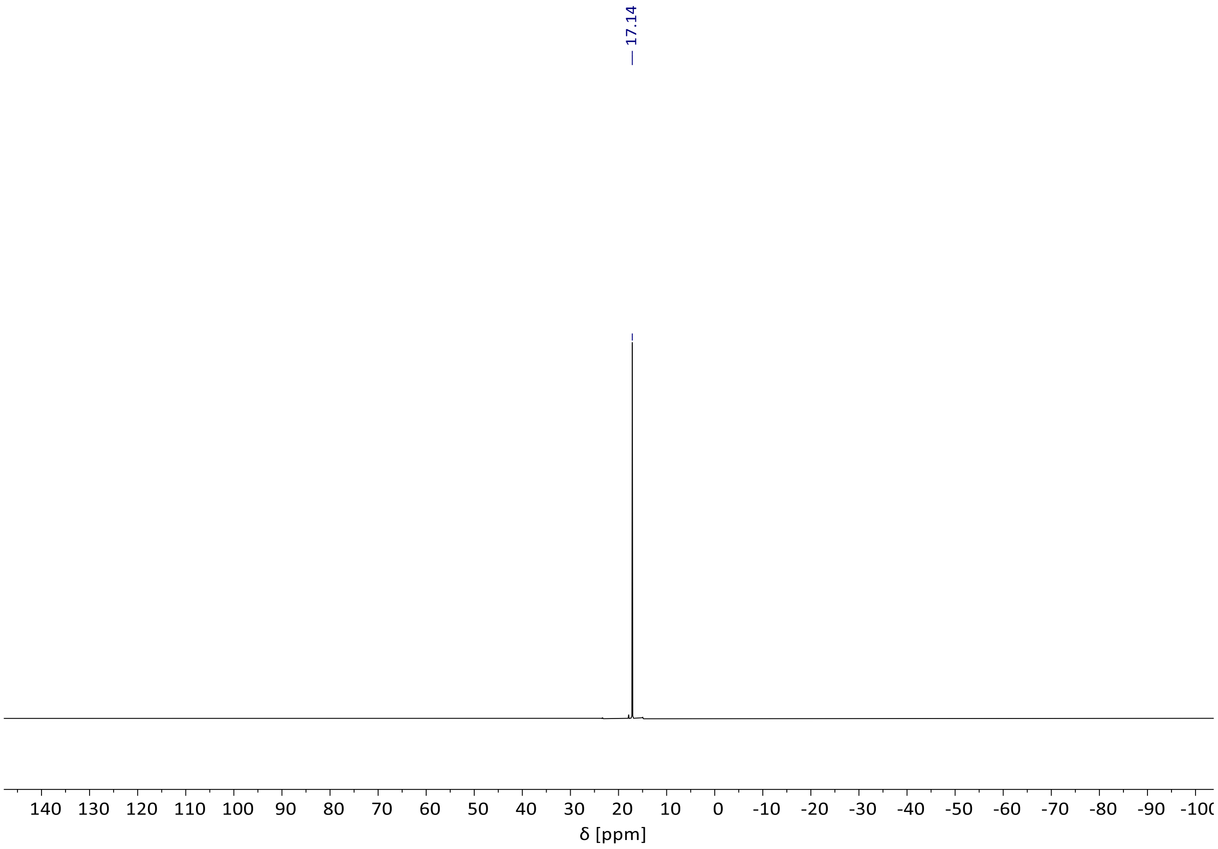

Figure S3. $^{31}P\{^{1}H\}$ NMR spectrum of **1b** as a solution in C_6D_6 at ambient temperature.

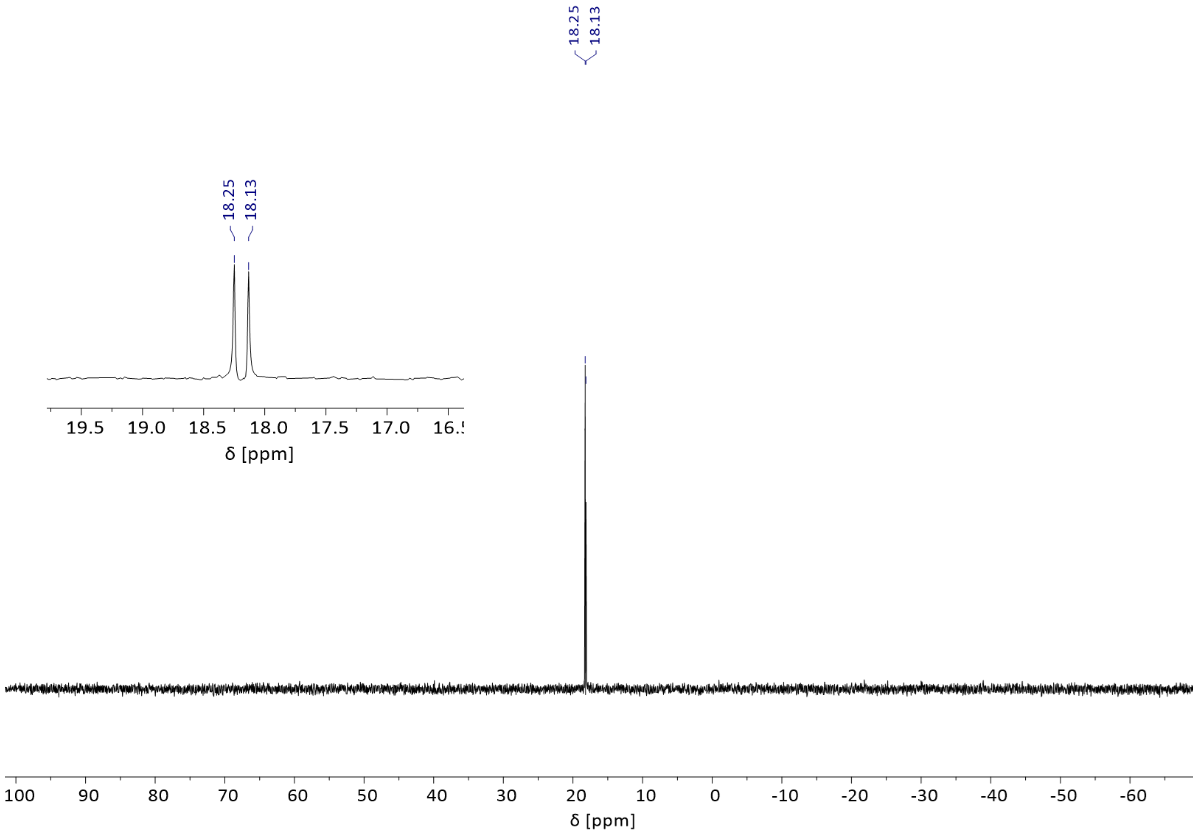

Figure S4. $^{29}Si\{^{1}H\}$ NMR spectrum of **1b** as a solution in C_6D_6 at ambient temperature.

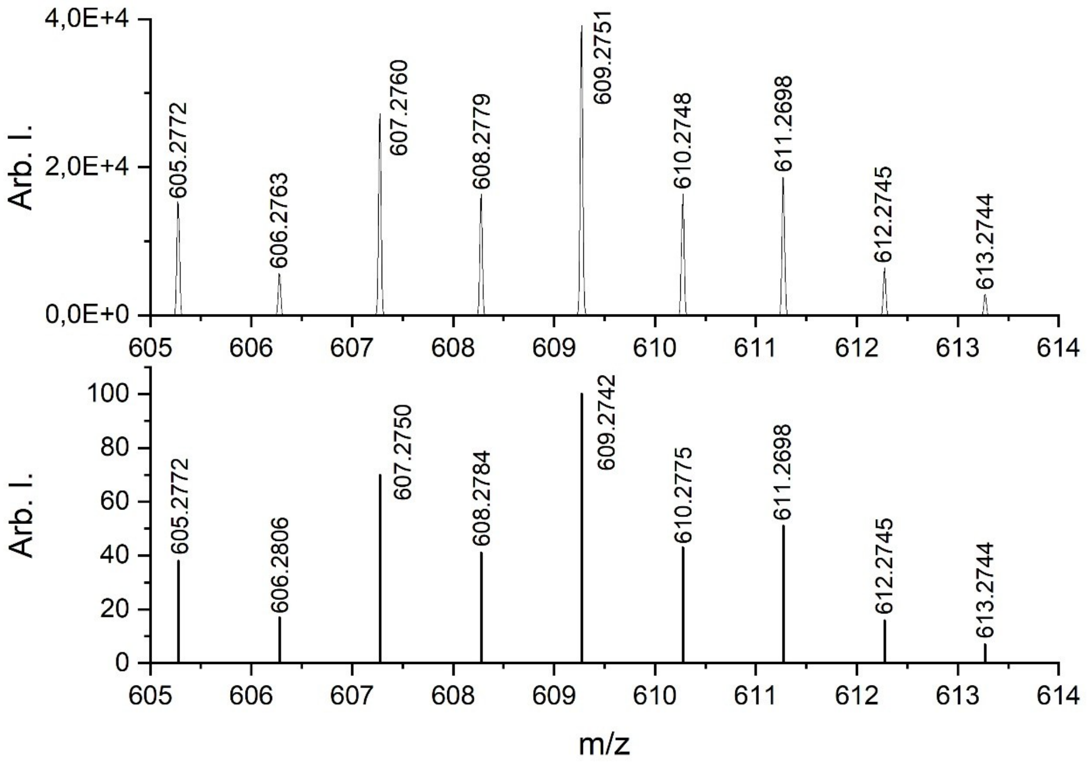

Figure S5. Cutout from LIFDI/MS of **1b**; **Top**: found MS for [M+H]; **Bottom**: Calculated MS spectrum of [M+H].

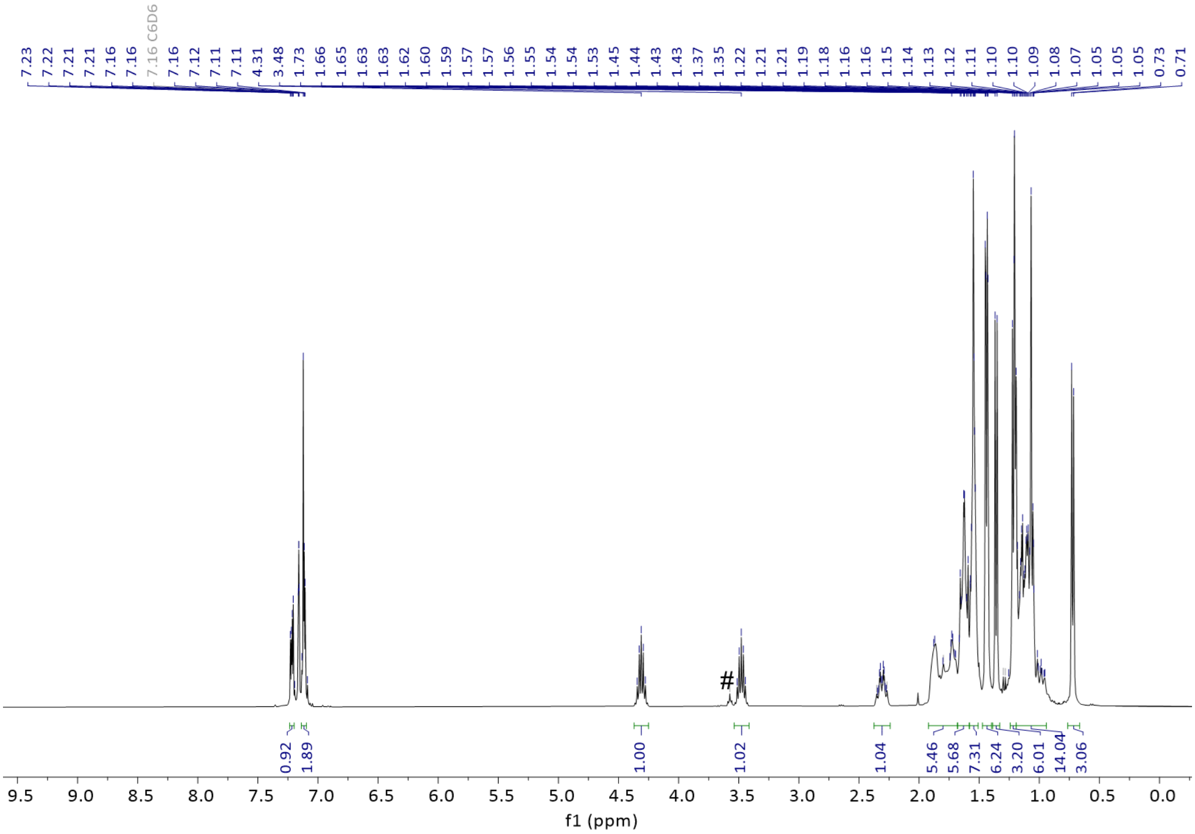

Figure S6. ^{1}H NMR spectrum of **2b** as a solution in C_6D_6 at ambient temperature; # marks an unknown impurity.

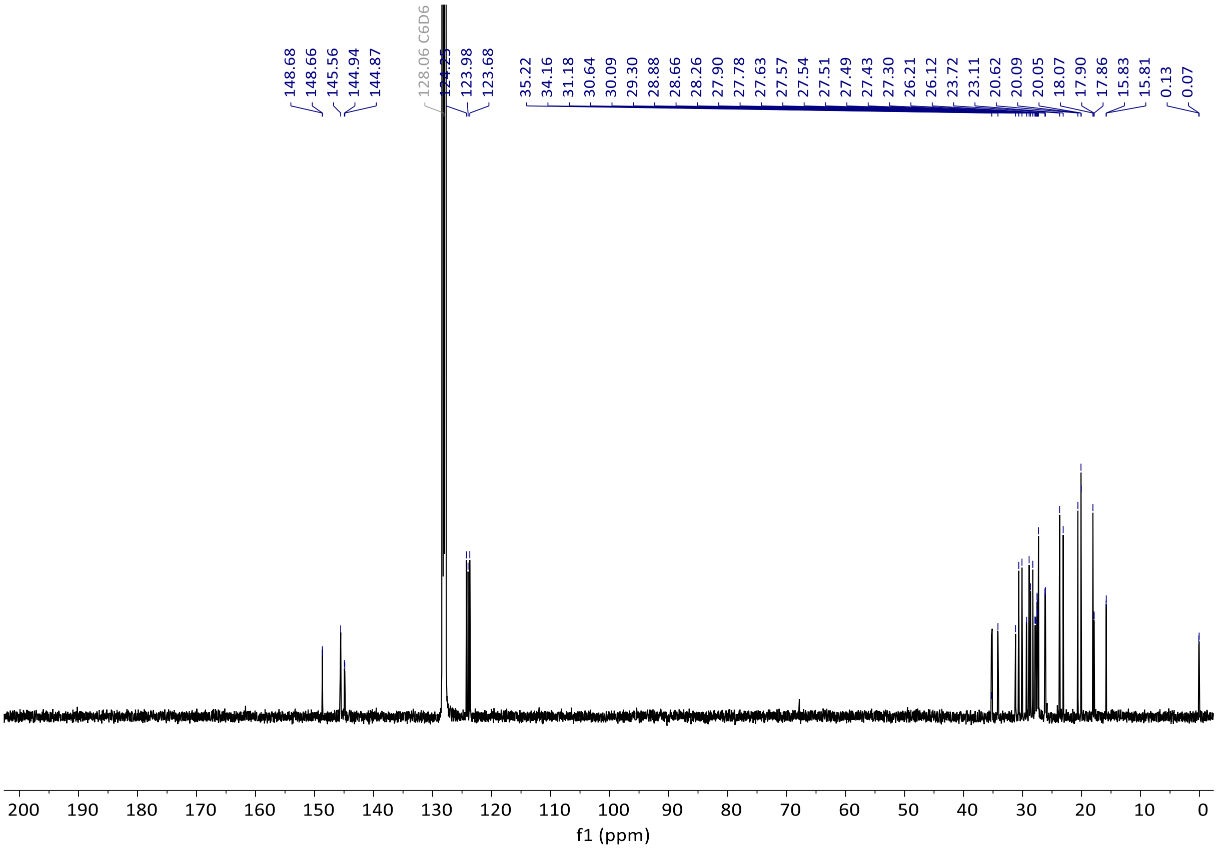

Figure S7. $^{13}C\{^1H\}$ NMR spectrum of **2b** as a solution in C_6D_6 at ambient temperature.

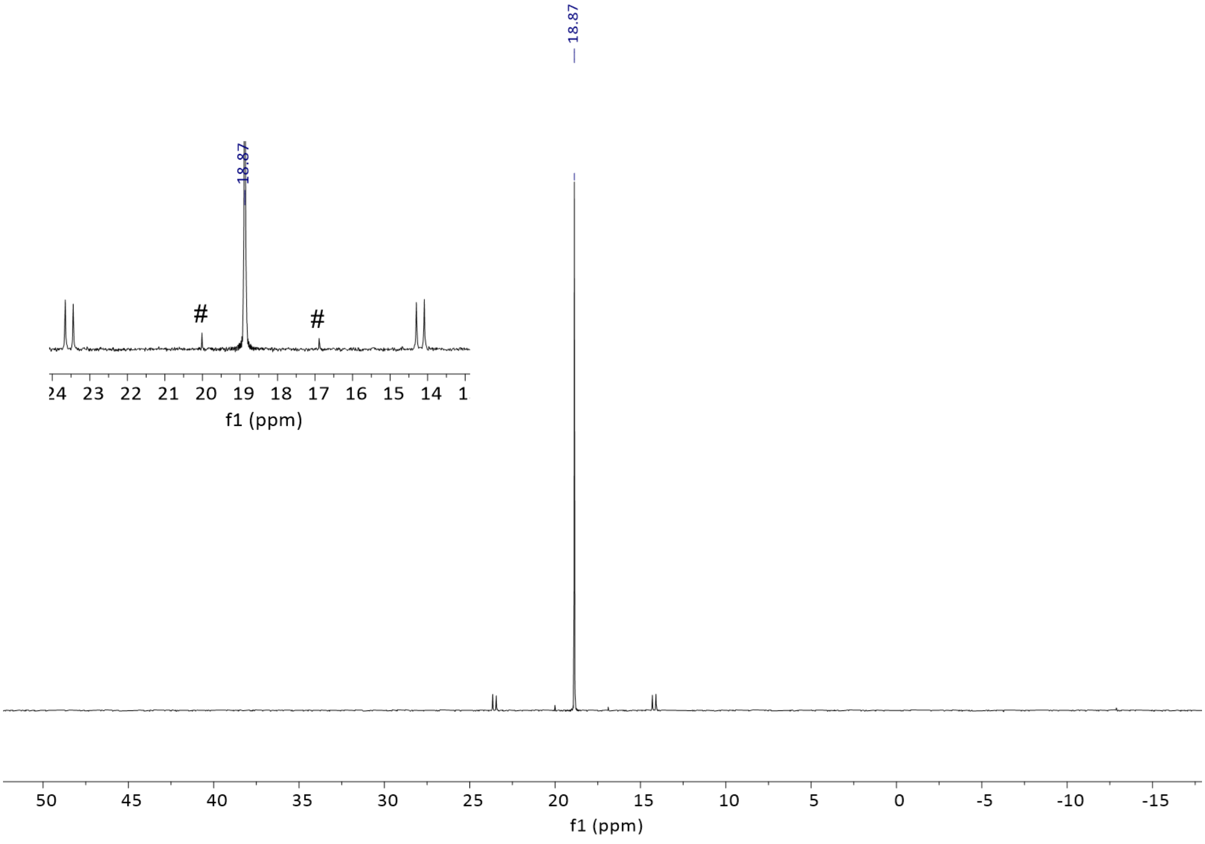

Figure S8. $^{31}P\{^1H\}$ NMR spectrum of **2b** as a solution in C_6D_6 at ambient temperature. # marks an unknown impurity.

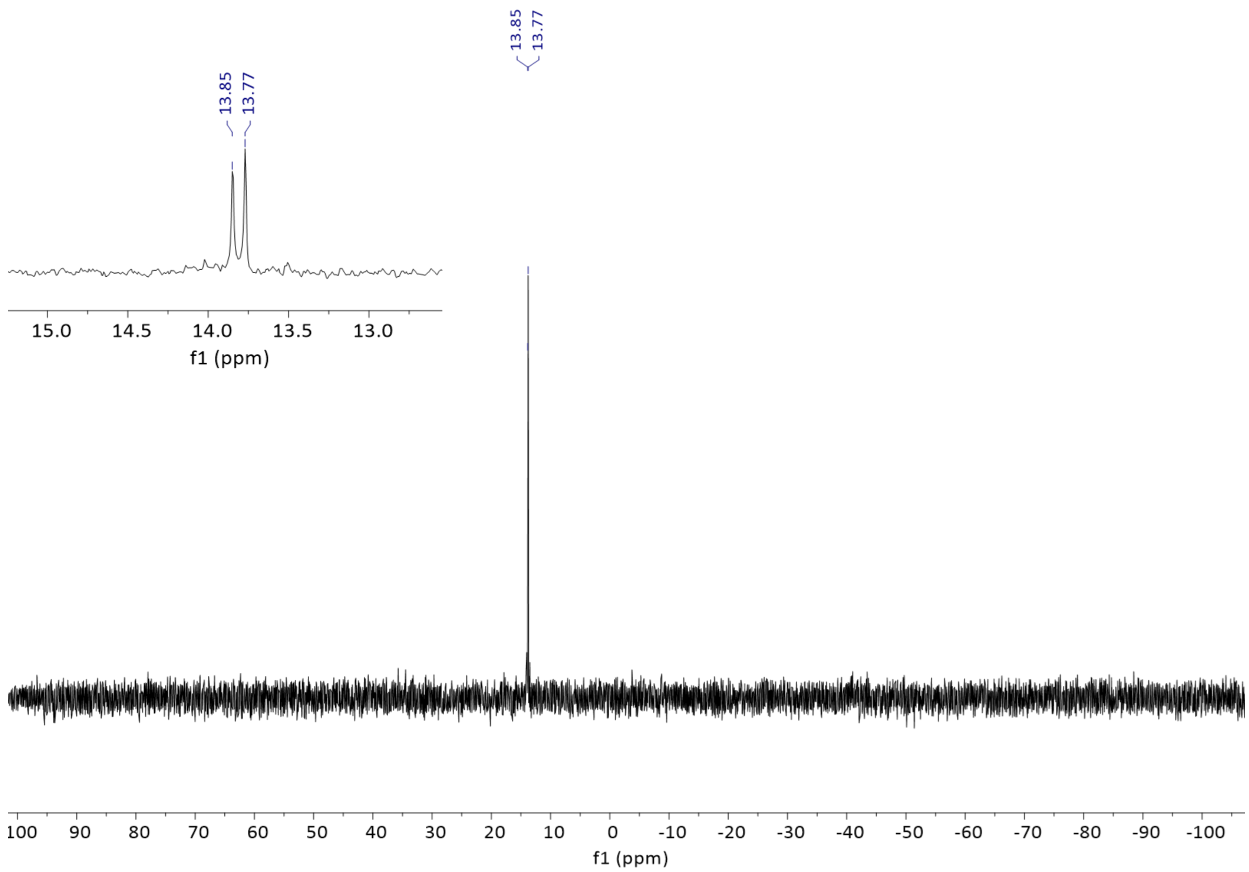

Figure S9. $^{29}Si\{^{1}H\}$ NMR spectrum of **2b** as a solution in C_6D_6 at ambient temperature.

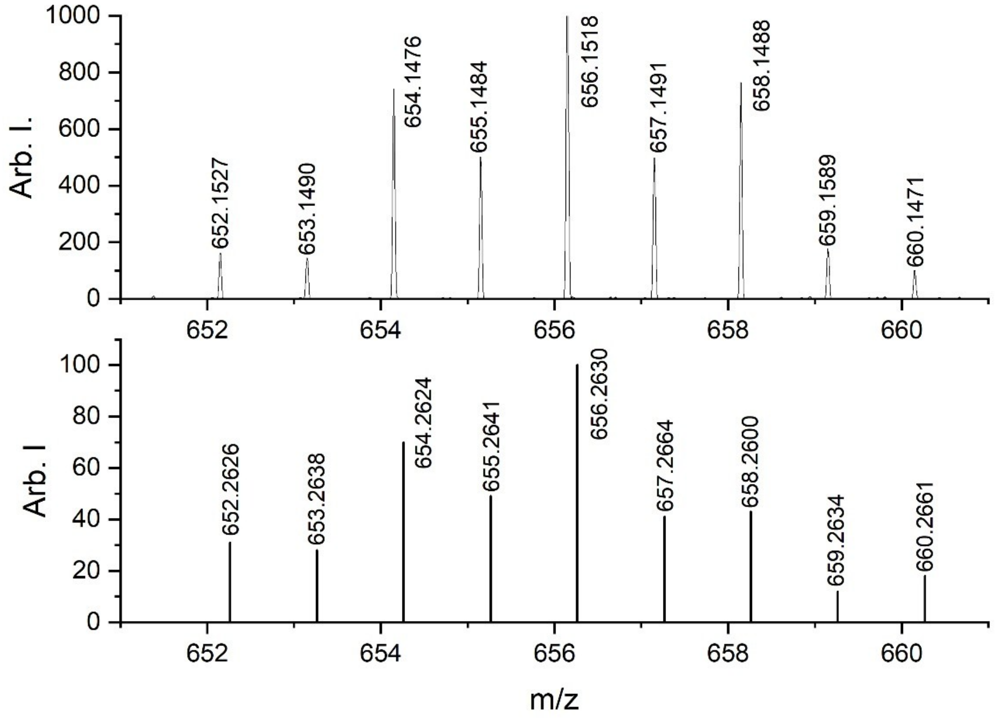

Figure S10. Cutout from LIFDI/MS of **2b**; **Top**: found MS for [M+H]; **Bottom**: Calculated MS spectrum of [M+H].

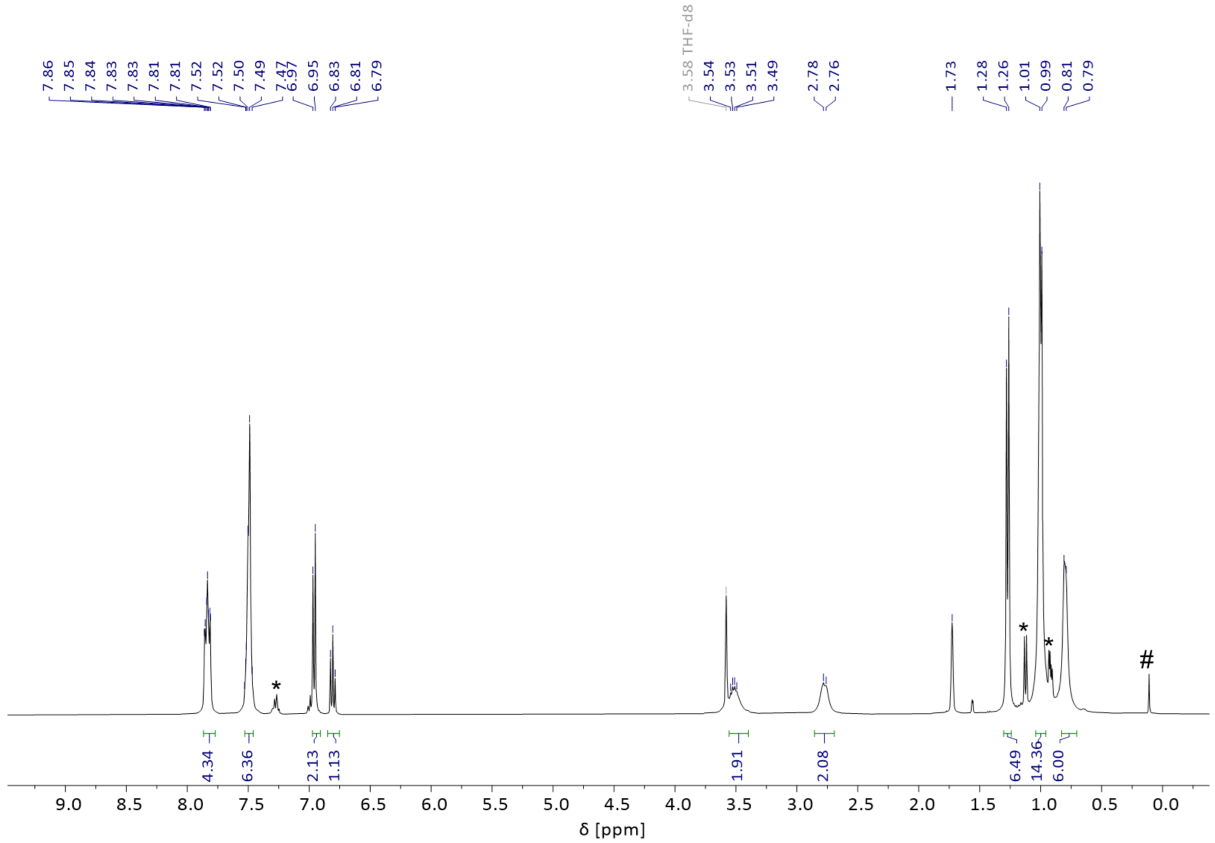

Figure S11. ^{1}H NMR spectrum of **3a** as a solution in THF-d^8 at ambient temperature; * indicates small amounts of free ligand ^{Ph}LH, while # marks an unknown impurity.

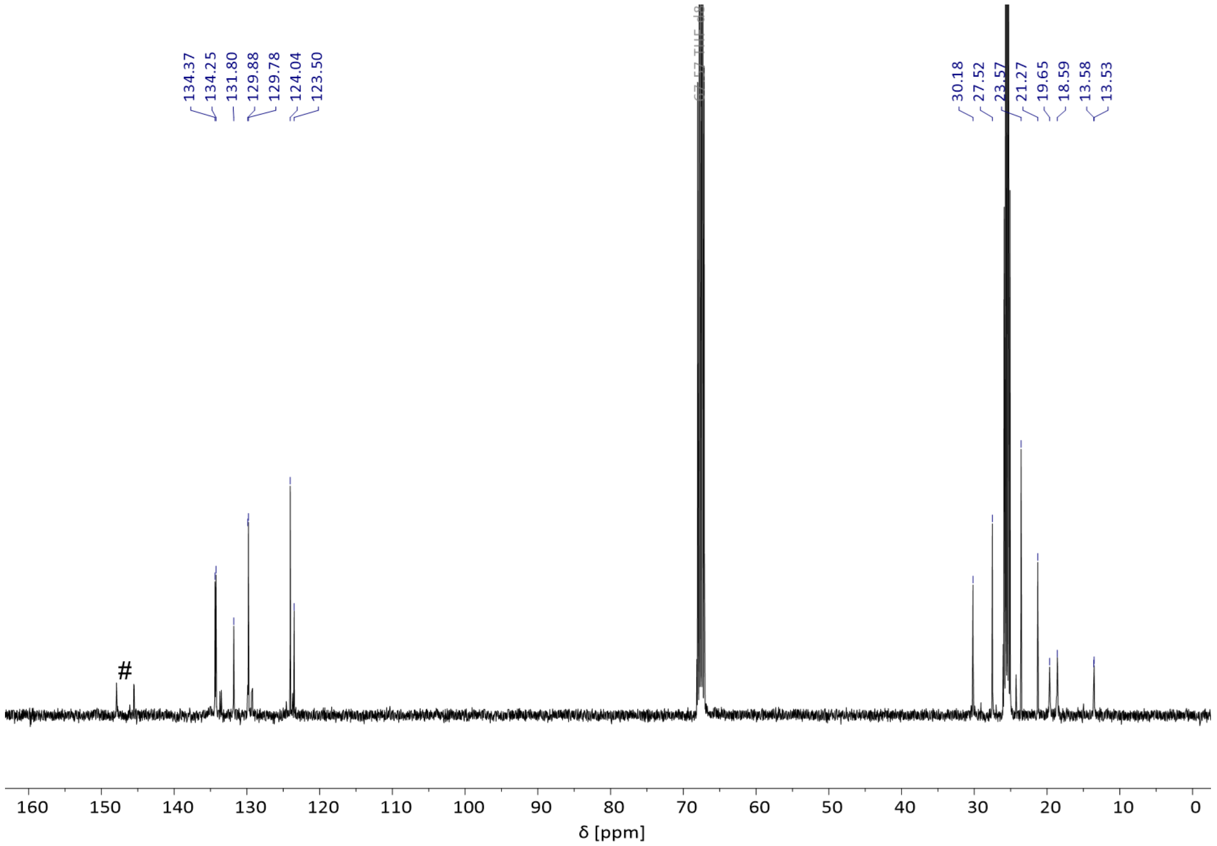

Figure S12. $^{13}C\{^{1}H\}$ NMR spectrum of **3a** as a solution in THF-d^8 at ambient temperature. # marks an unknown impurity.

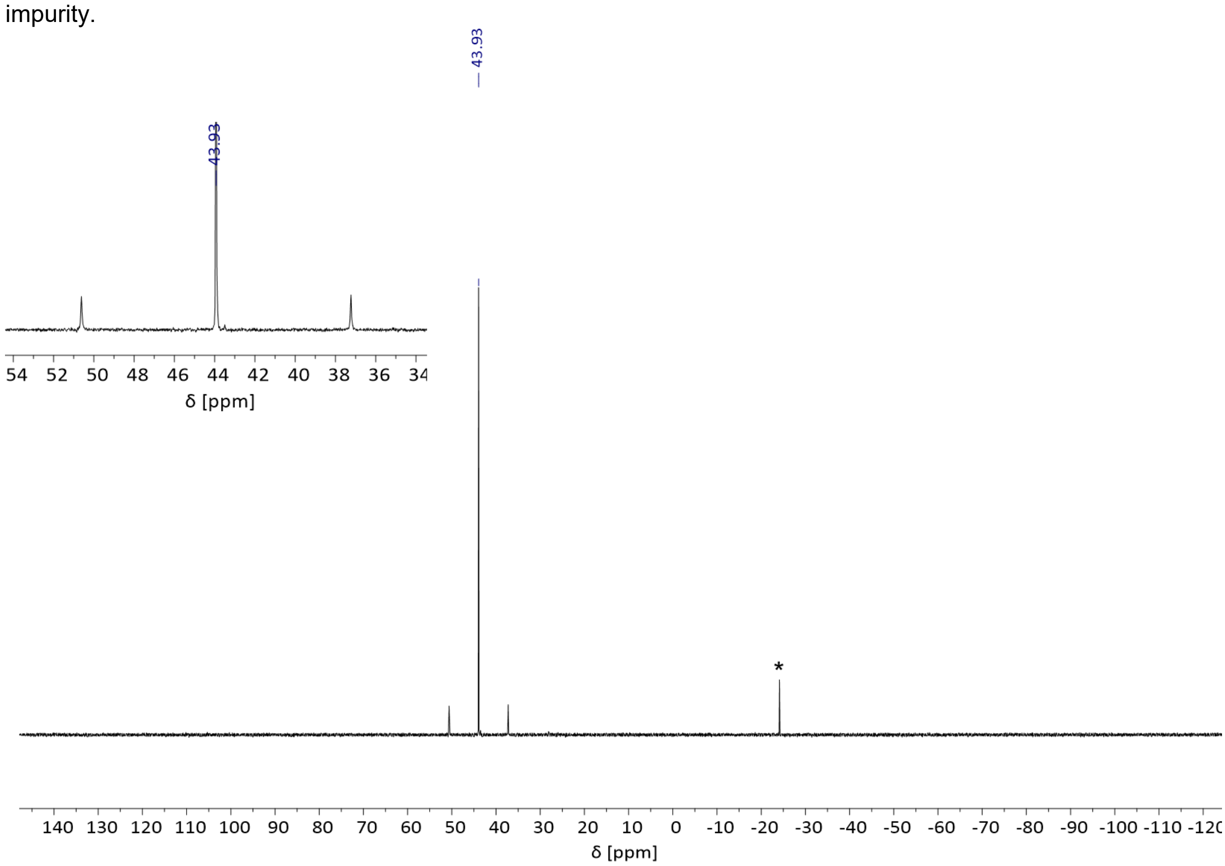

Figure S13. $^{31}P\{^{1}H\}$ NMR spectrum of **3a** as a solution in THF-d^8 at ambient temperature; * indicates small amounts of free ligand ^{Ph}LH.

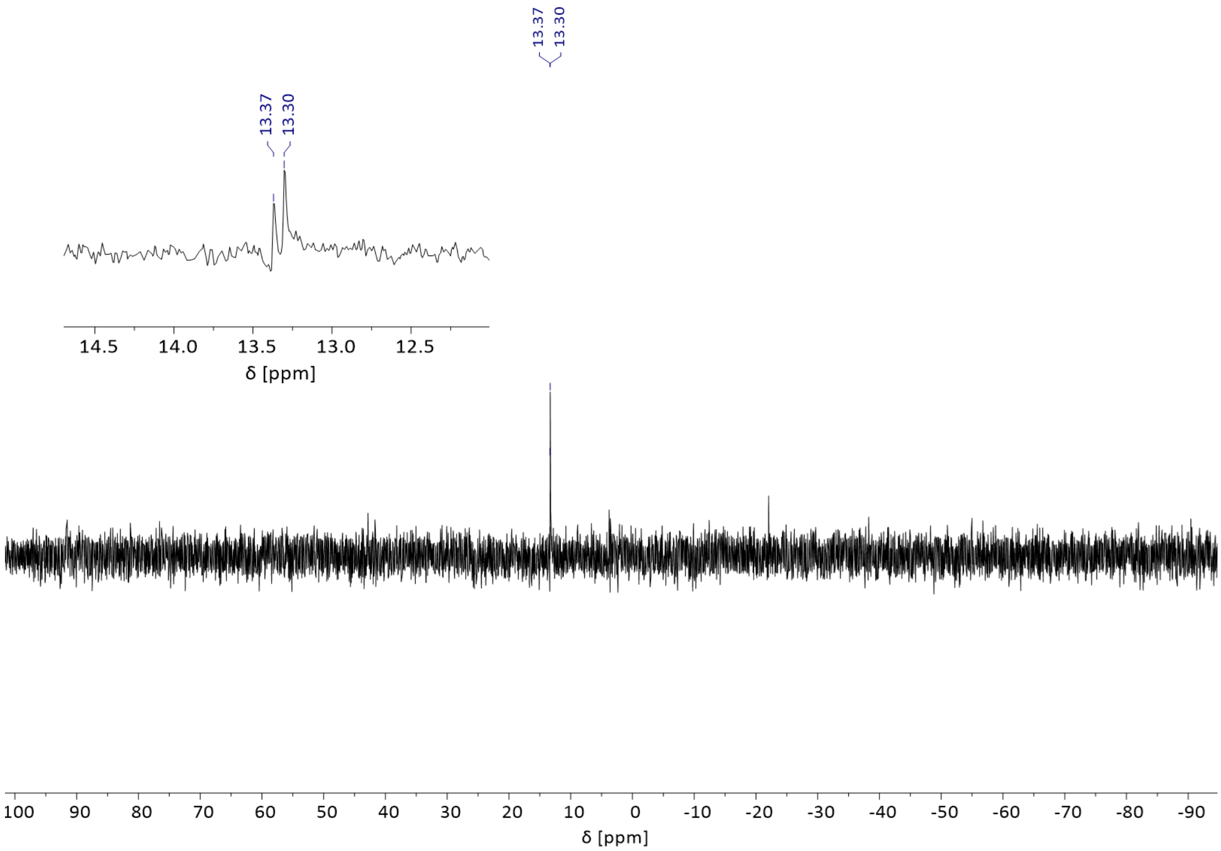

Figure S14. $^{29}Si\{^{1}H\}$ NMR spectrum of **3a** as a solution in THF-d^8 at ambient temperature.

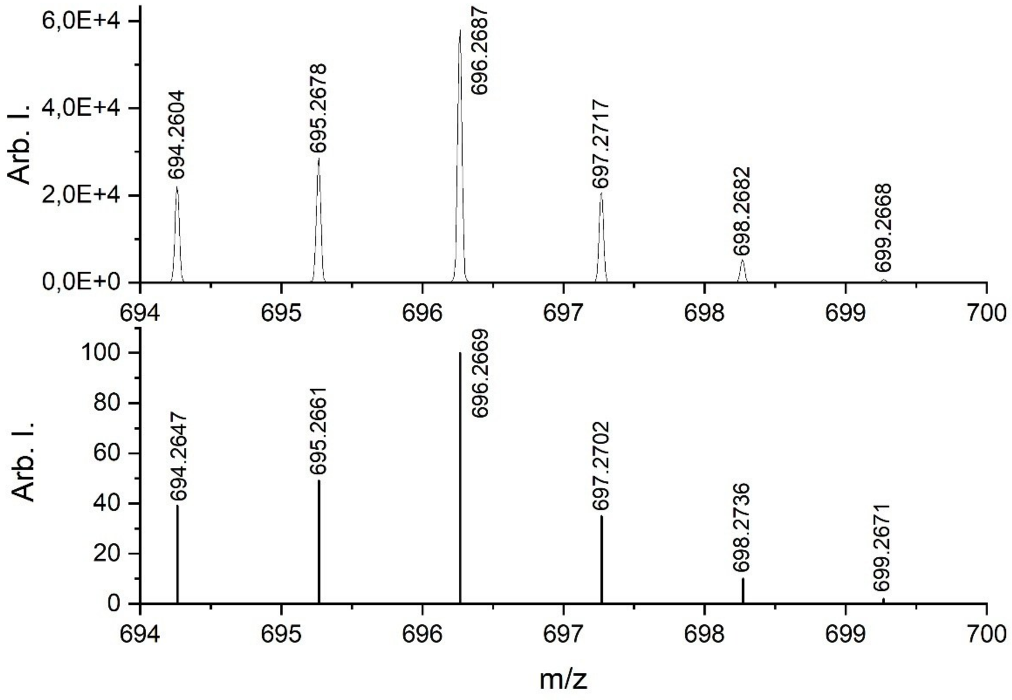

Figure S15. Cutout from LIFDI/MS of **3a**; **Top**: found MS for [M-Br]; **Bottom**: Calculated MS spectrum of [M-Br].

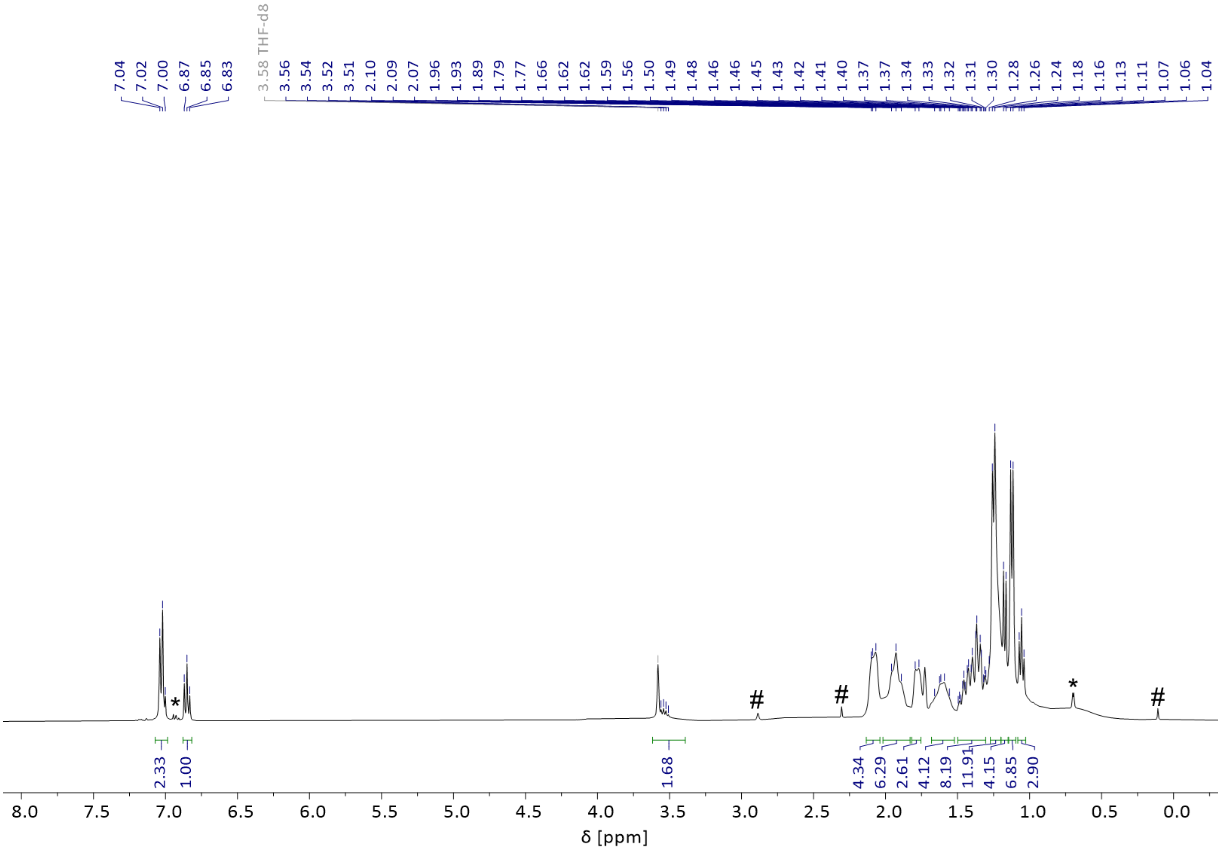

Figure S16. ^{1}H NMR spectrum of **3b** as a solution in THF-d^8 at ambient temperature; * indicates small amounts of free ligand ^{Cy}LH, while # marks an unknown impurity.

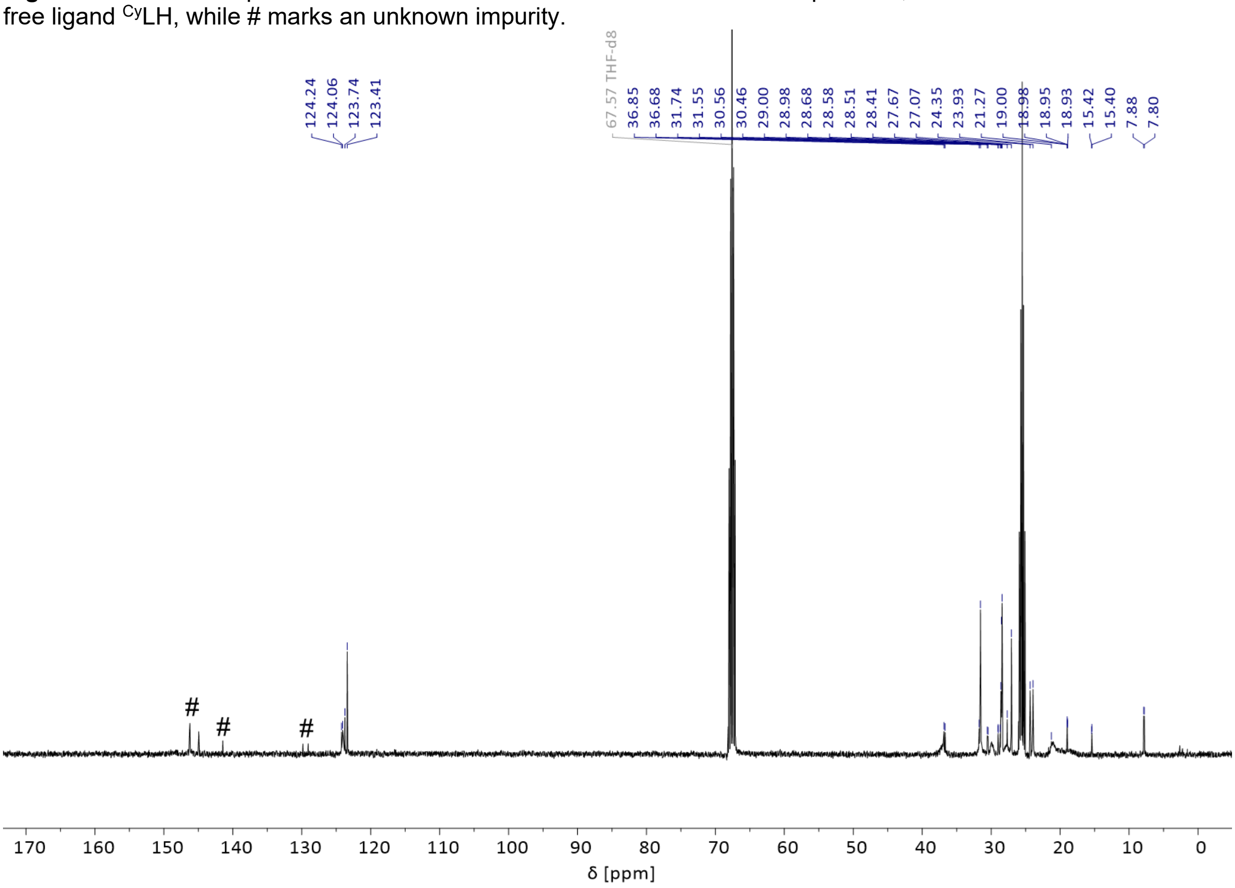

Figure S17. $^{13}C\{^{1}H\}$ NMR spectrum of **3b** as a solution in THF-d^8 at ambient temperature; # marks an unknown impurity.

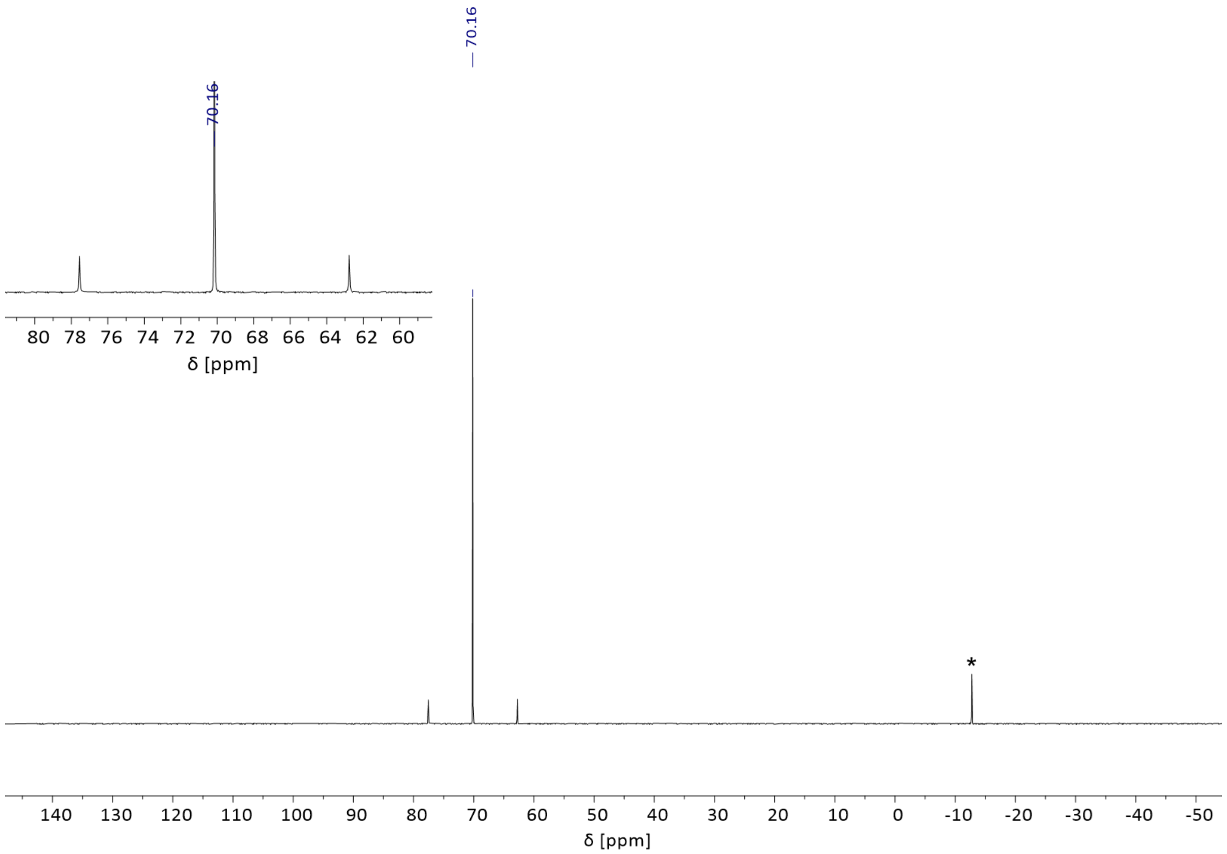

Figure S18. $^{31}P\{^1H\}$ NMR spectrum of **3b** as a solution in THF-d^8 at ambient temperature; * indicates small amounts of free ligand ^{Cy}LH.

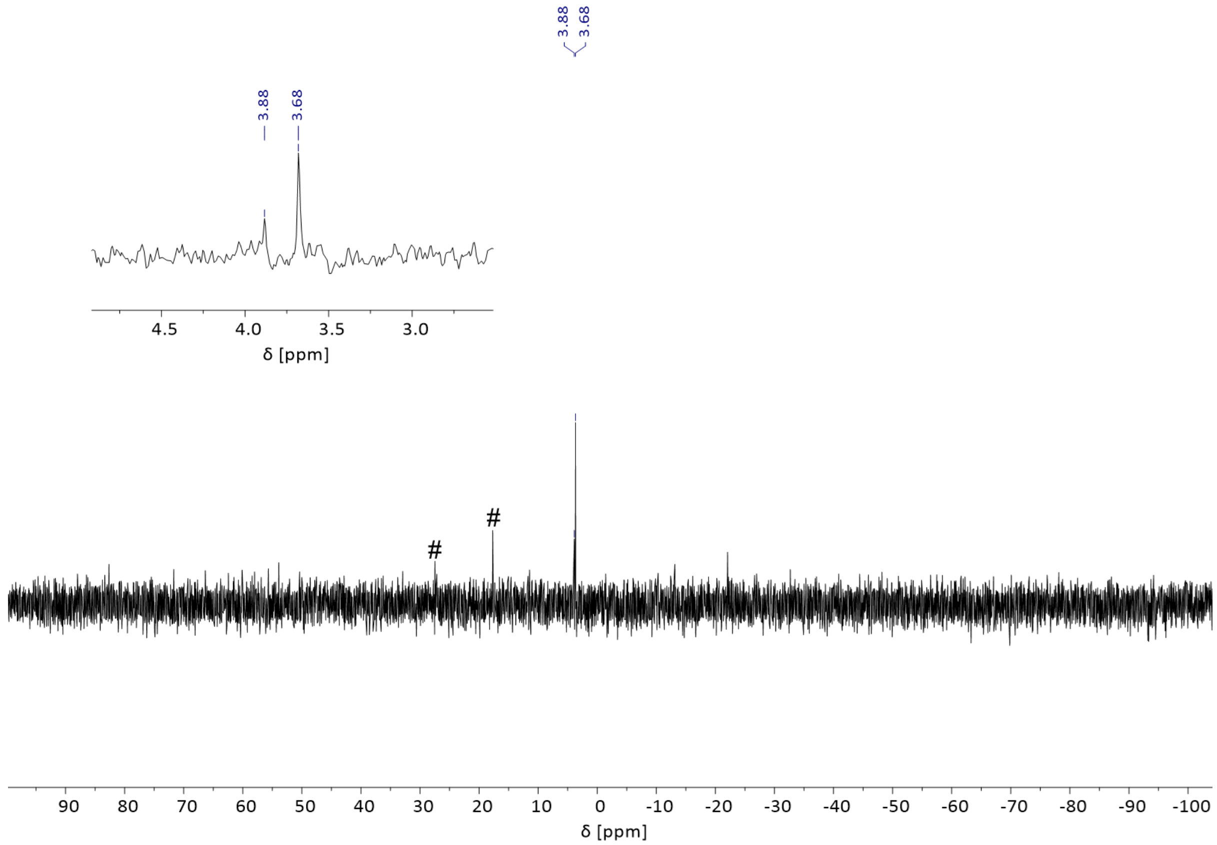

Figure S19. $^{29}Si\{^1H\}$ NMR spectrum of **3b** as a solution in THF-d^8 at ambient temperature. # marks an unknown impurity.

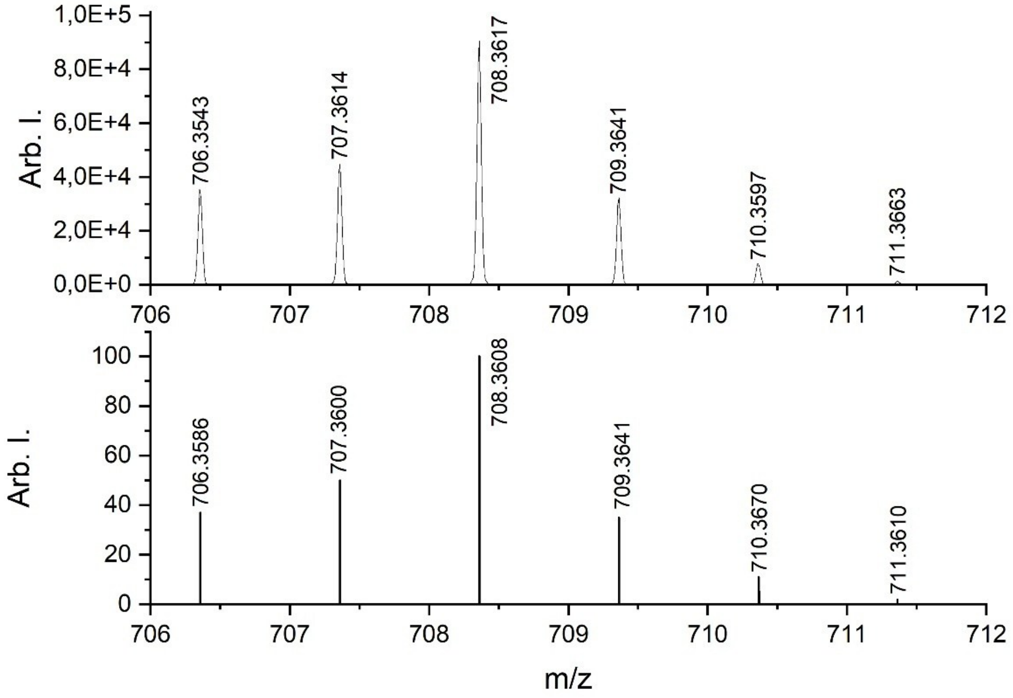

Figure S20. Cutout from LIFDI/MS of **3b**; **Top**: found MS for [M-Br]; **Bottom**: Calculated MS spectrum of [M-Br].

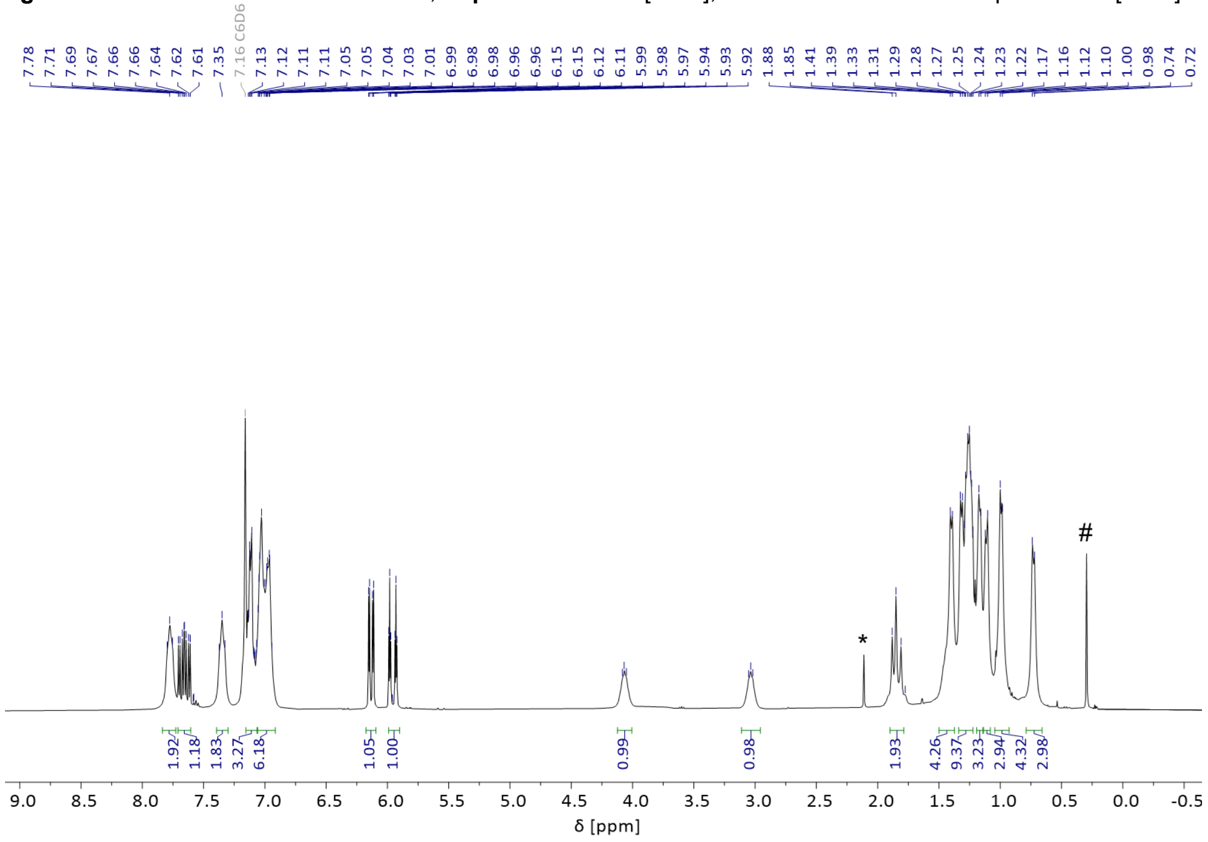

Figure S21. ^{1}H NMR spectrum of **4a** as a solution in C_6D_6 at ambient temperature; * indicates small amounts of toluene, while # marks an unknown impurity.

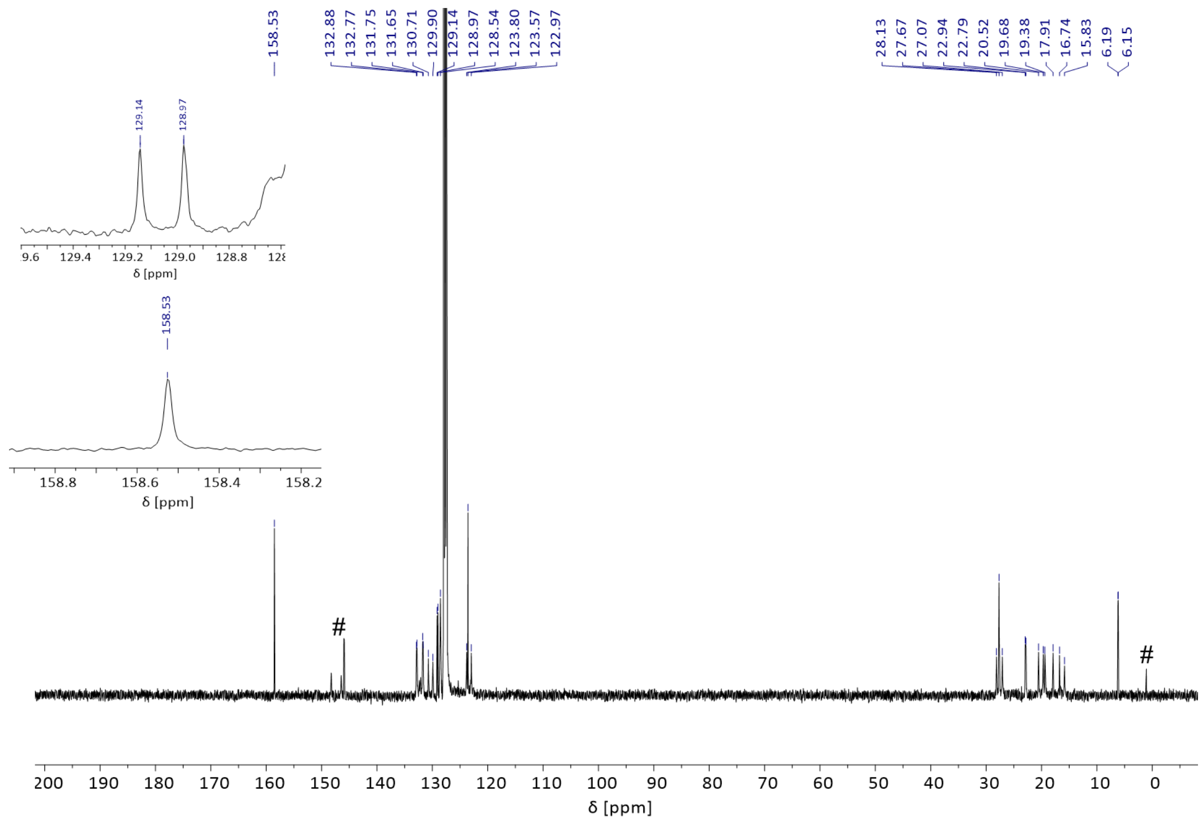

Figure S22. $^{13}C\{^{1}H\}$ NMR spectrum of **4a** as a solution in C_6D_6 at ambient temperature; # marks an unknown impurity.

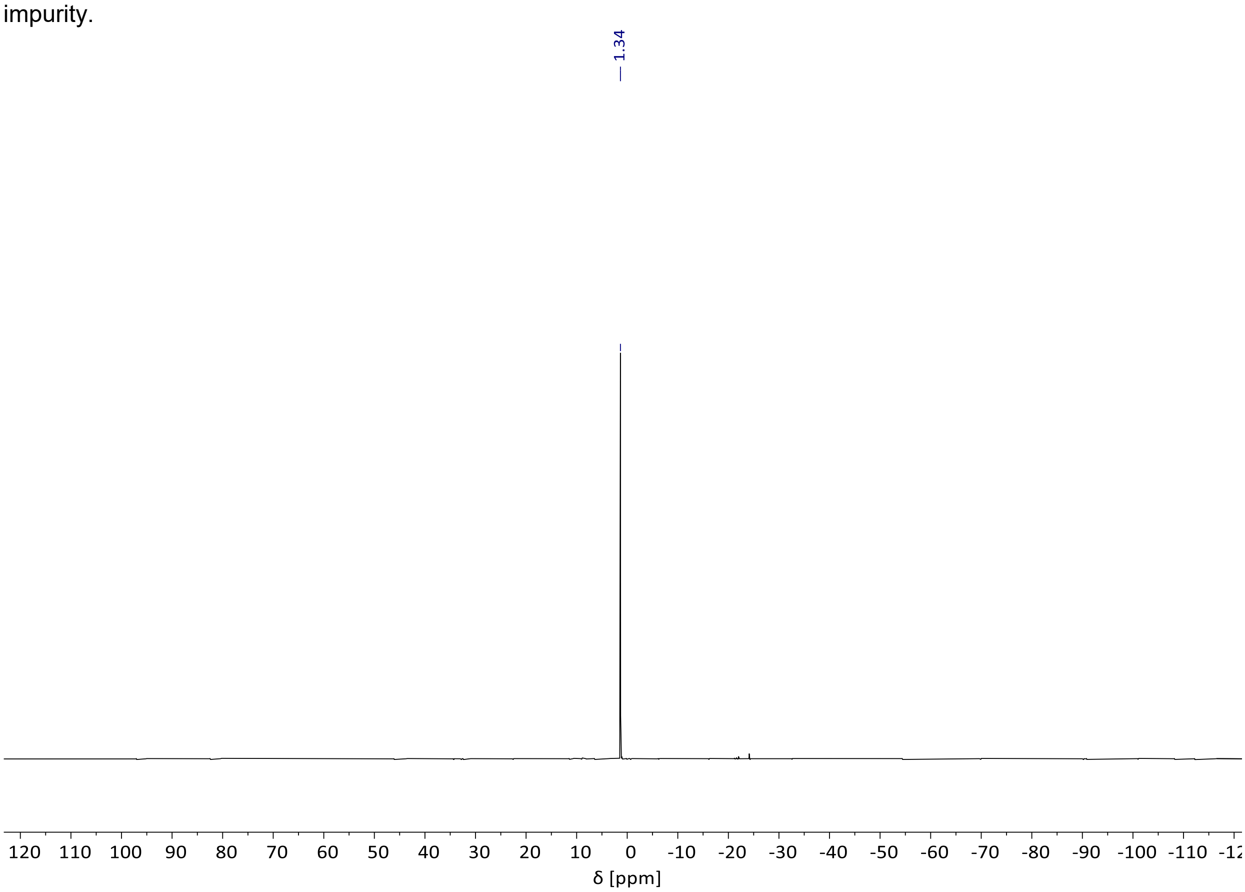

Figure S23. $^{31}P\{^{1}H\}$ NMR spectrum of **4a** as a solution in C_6D_6 at ambient temperature.

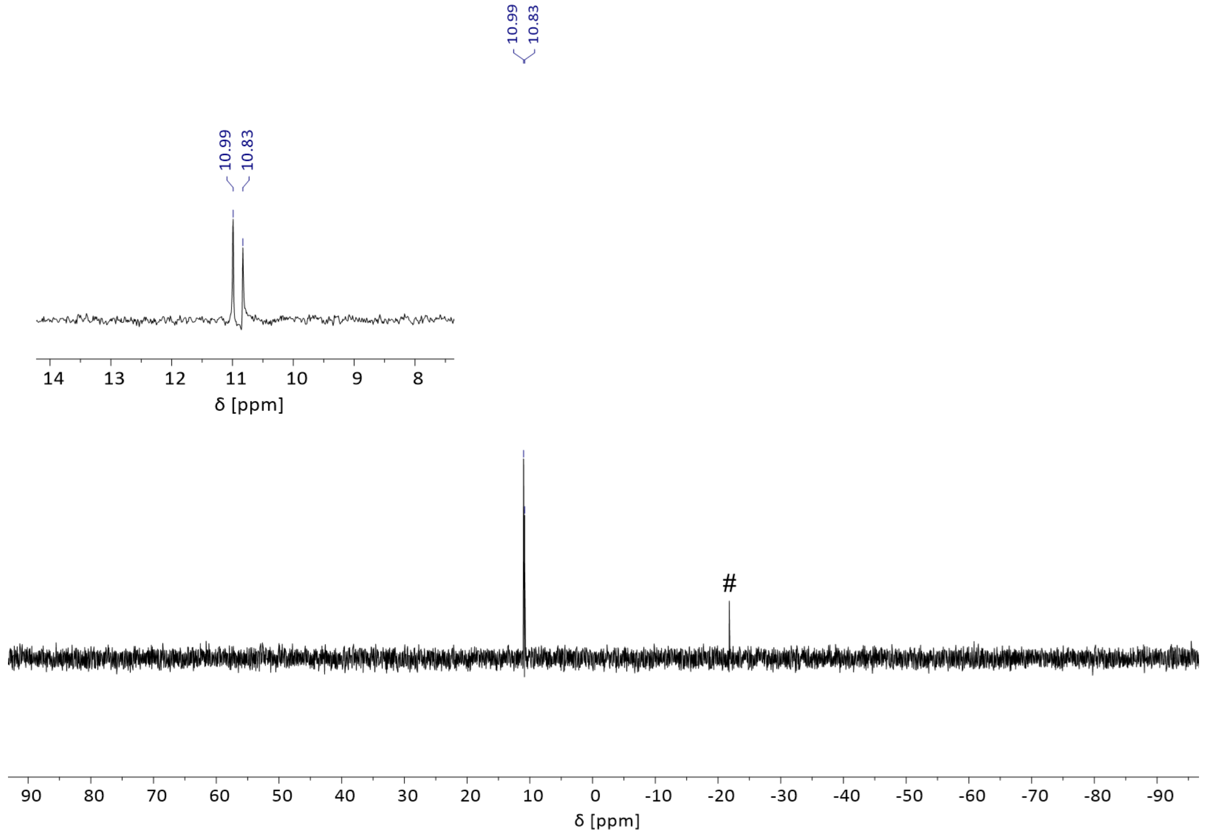

Figure S24. $^{29}Si\{^1H\}$ NMR spectrum of **4a** as a solution in C_6D_6 at ambient temperature; # marks an unknown impurity.

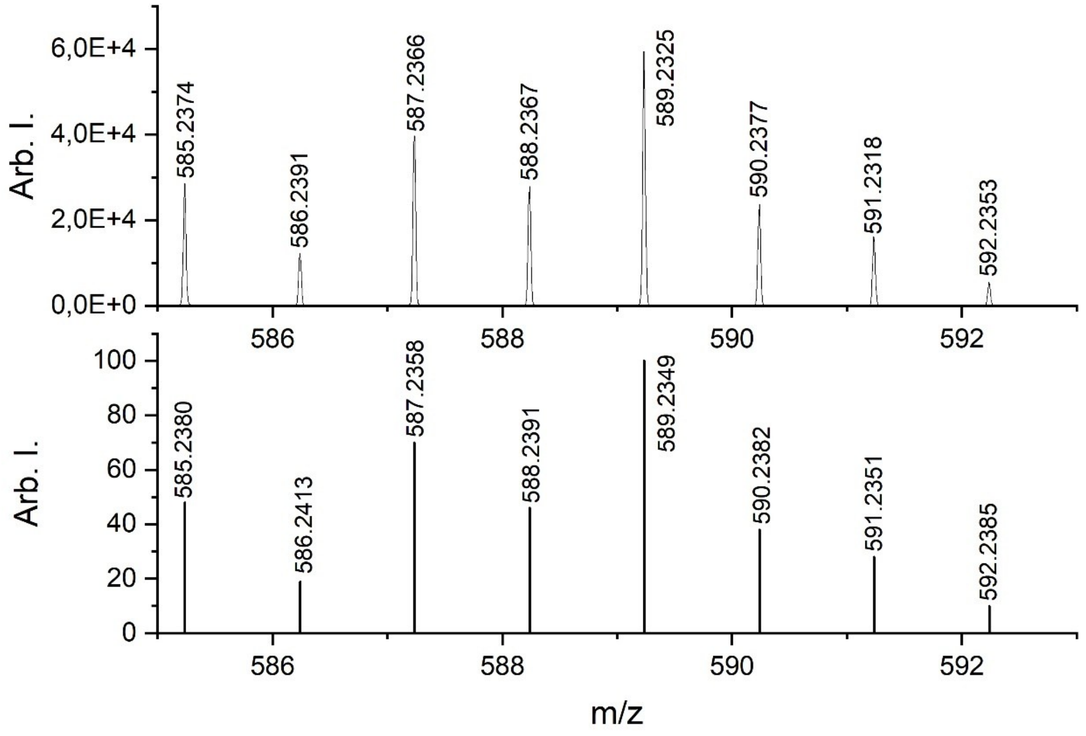

Figure S25.Top: Cutout from LIFDI/MS of **4a**; **Top**: found MS for [M]; **Bottom**: Calculated MS spectrum of [M].

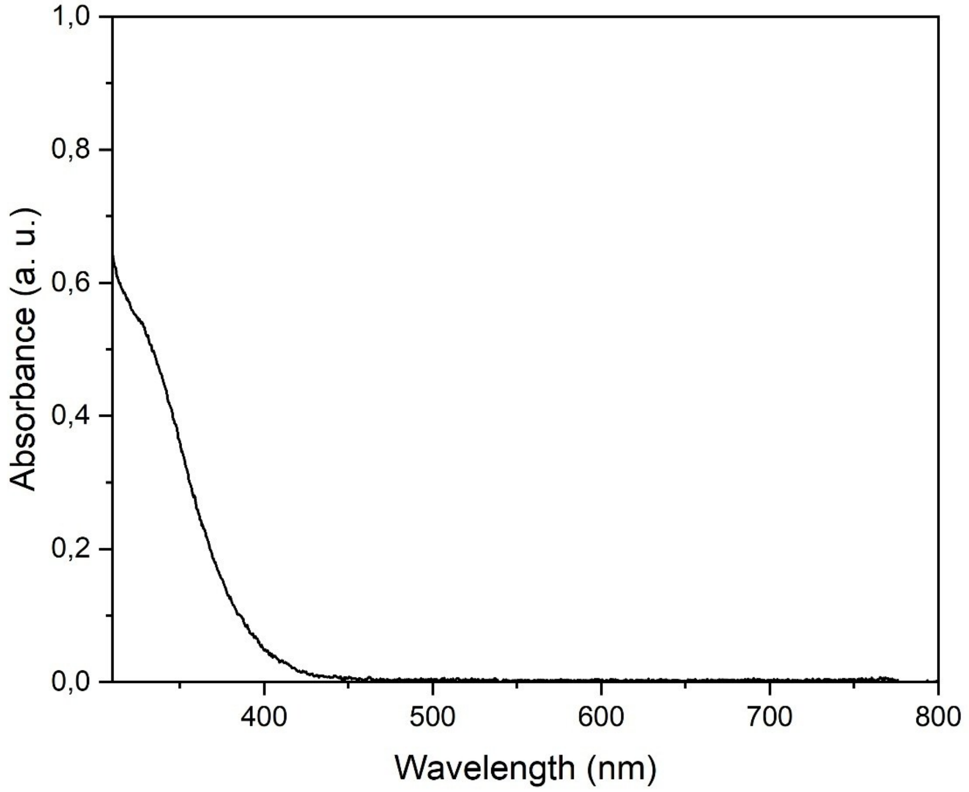

Figure S26. UV/Vis spectrum of a 1x10^{-3} M solution of **4a** in toluene at ambient temperature.

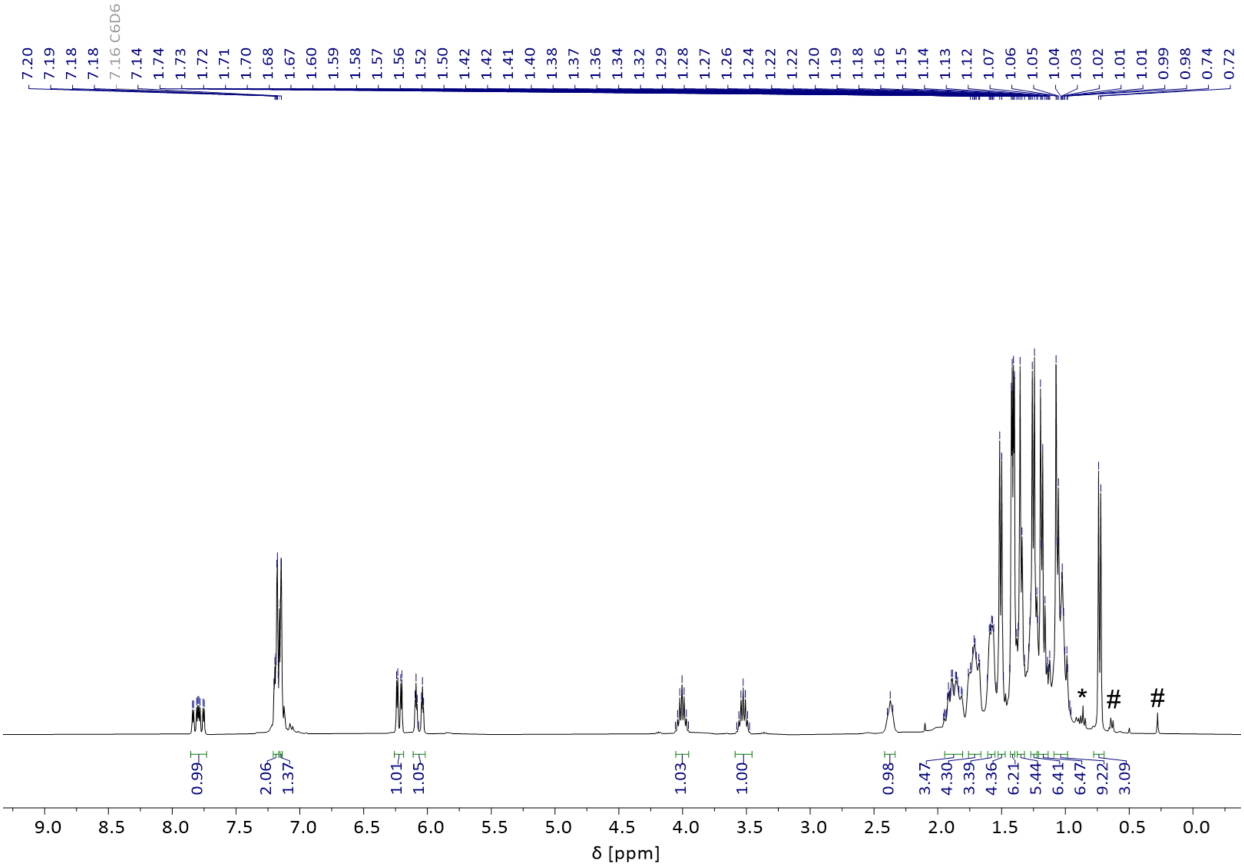

Figure S27. ^{1}H NMR spectrum of **4b** as a solution in C_6D_6 at ambient temperature; * indicates small amounts of pentane, while # marks an unknown impurity.

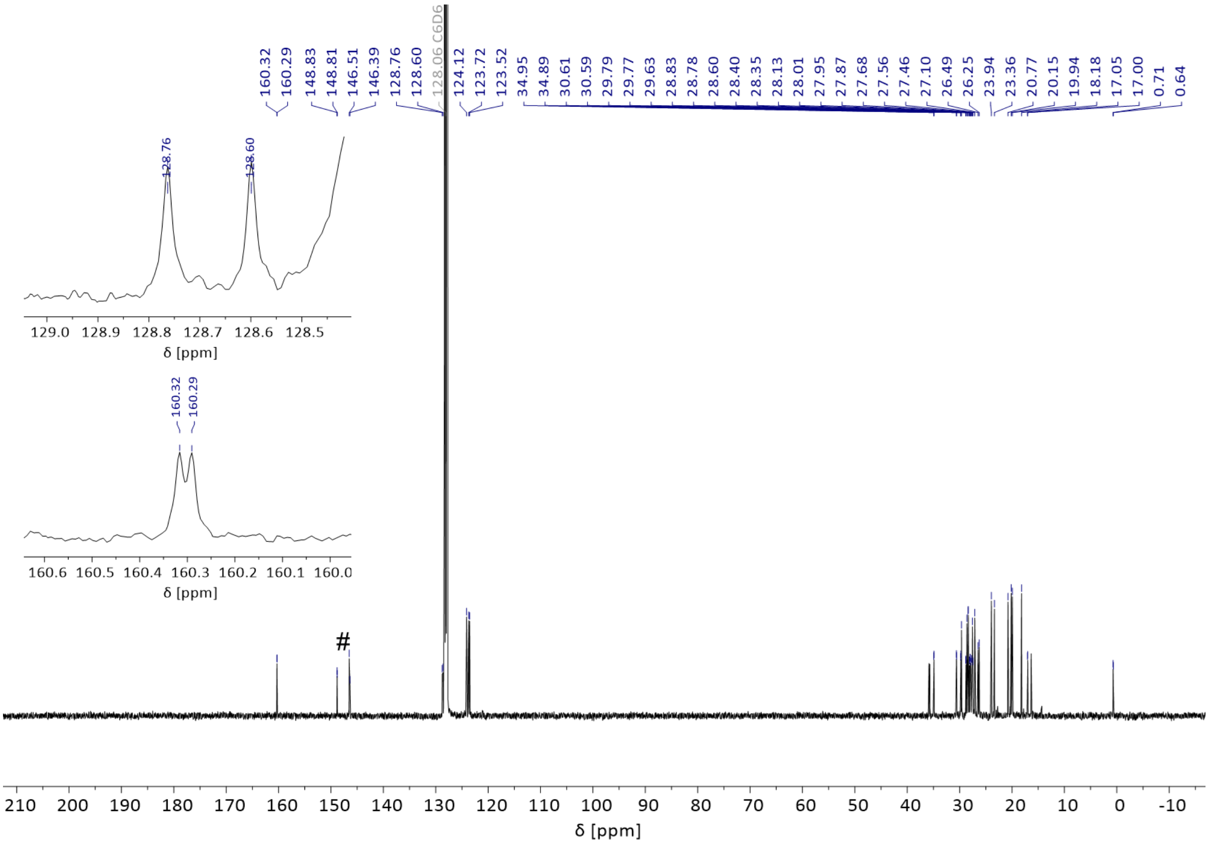

Figure S28. $^{13}C\{^{1}H\}$ NMR spectrum of **4b** as a solution in C_6D_6 at ambient temperature. # marks an unknown impurity.

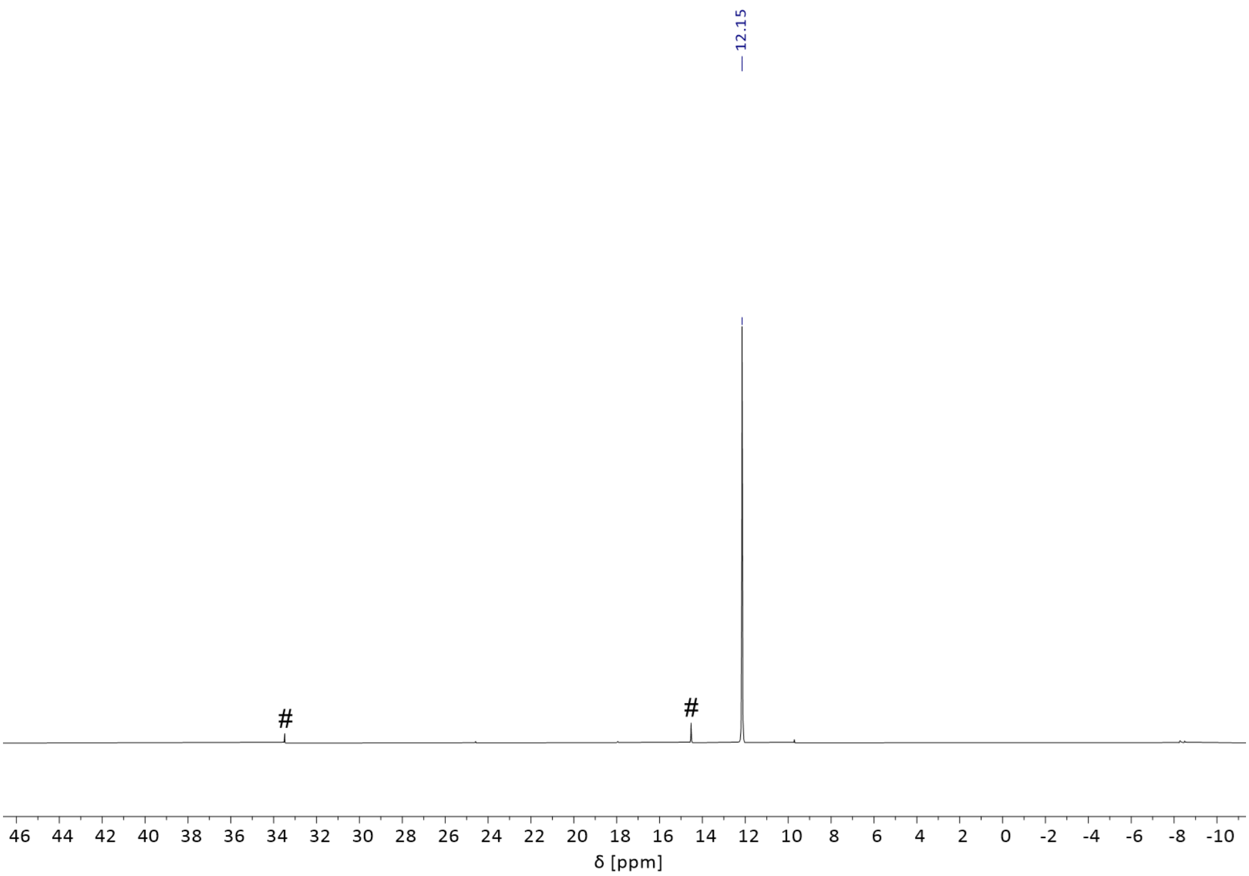

Figure S29. $^{31}P\{^1H\}$ NMR spectrum of **4b** as a solution in C_6D_6 at ambient temperature; # marks an unknown impurity.

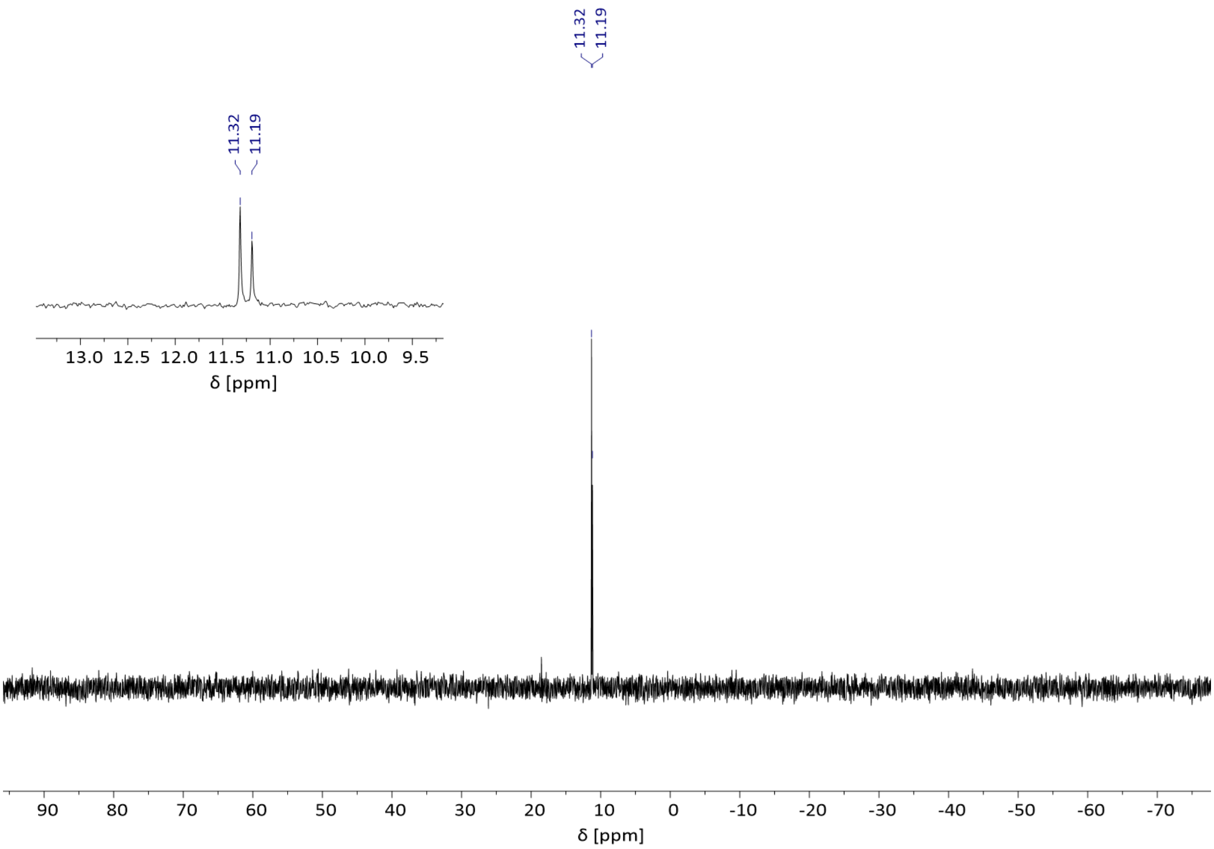

Figure S30. $^{29}Si\{^1H\}$ NMR spectrum of **4b** as a solution in C_6D_6 at ambient temperature.

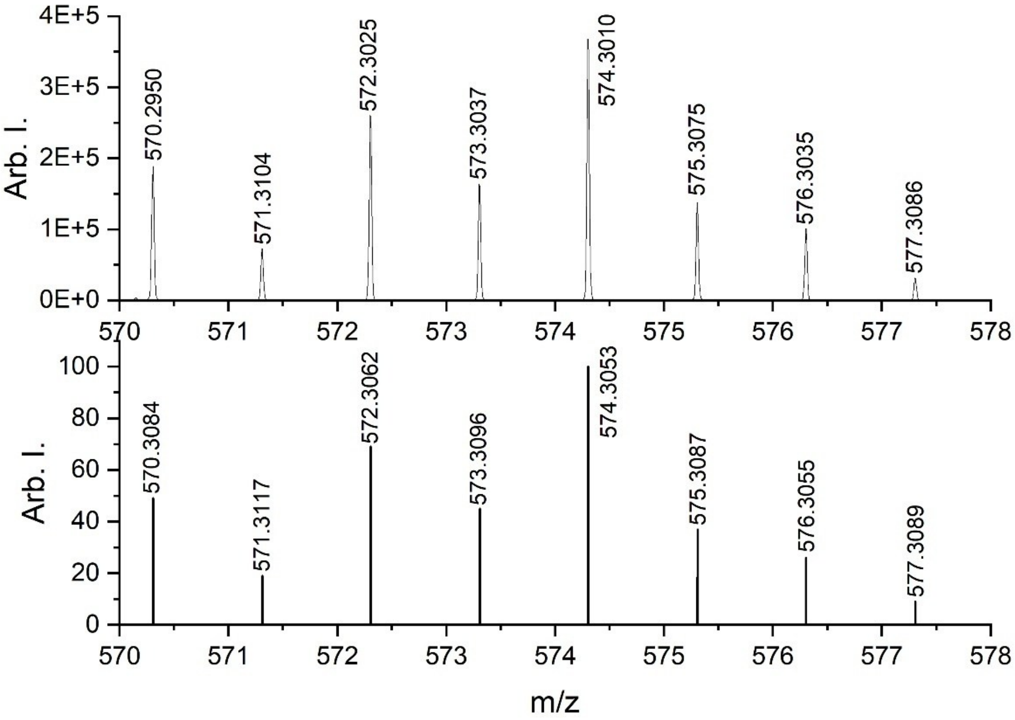

Figure S31. Cutout from LIFDI/MS of **4b**; **Top**: found MS for [M-C_2H_3]; **Bottom**: Calculated MS spectrum of [M-C_2H_3].

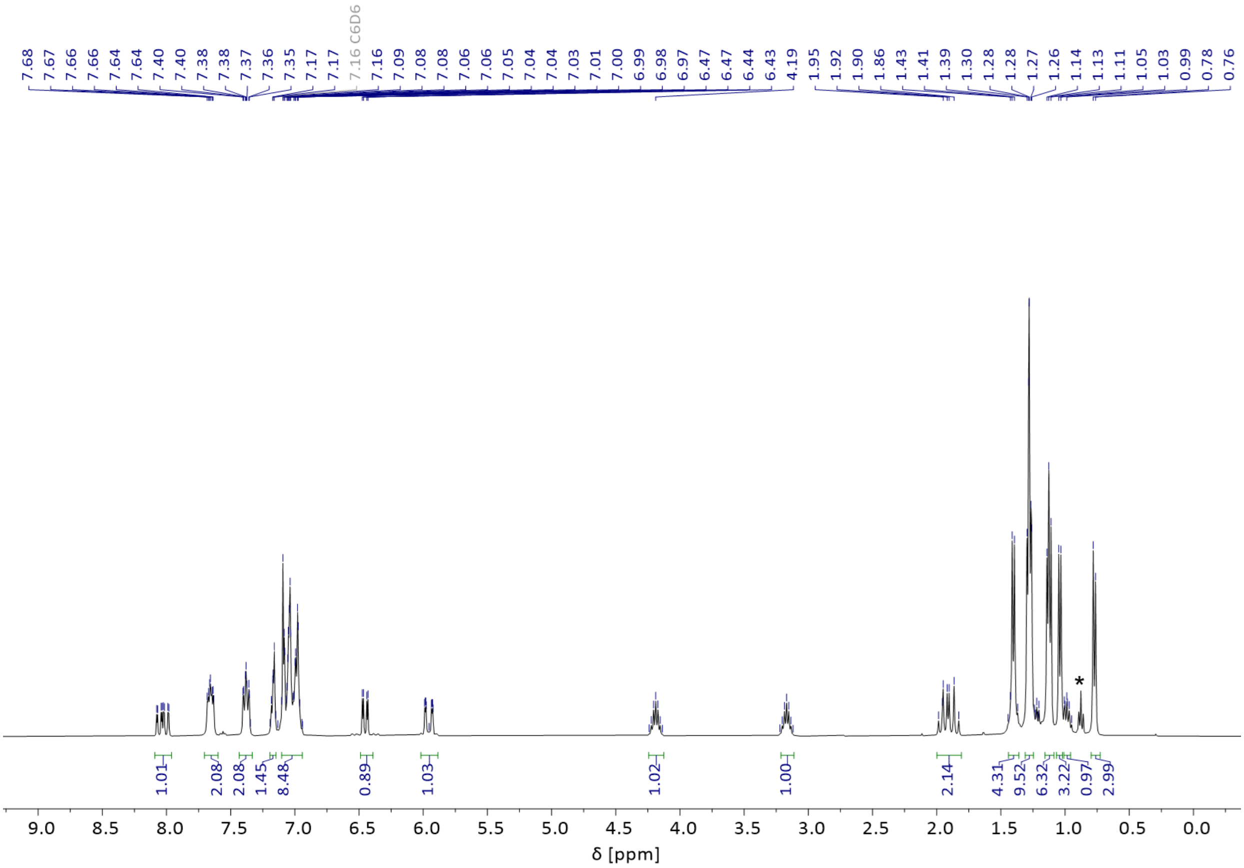

Figure S32. ^{1}H NMR spectrum of **5a** as a solution in C_6D_6 at ambient temperature; * indicates small amounts of pentane.

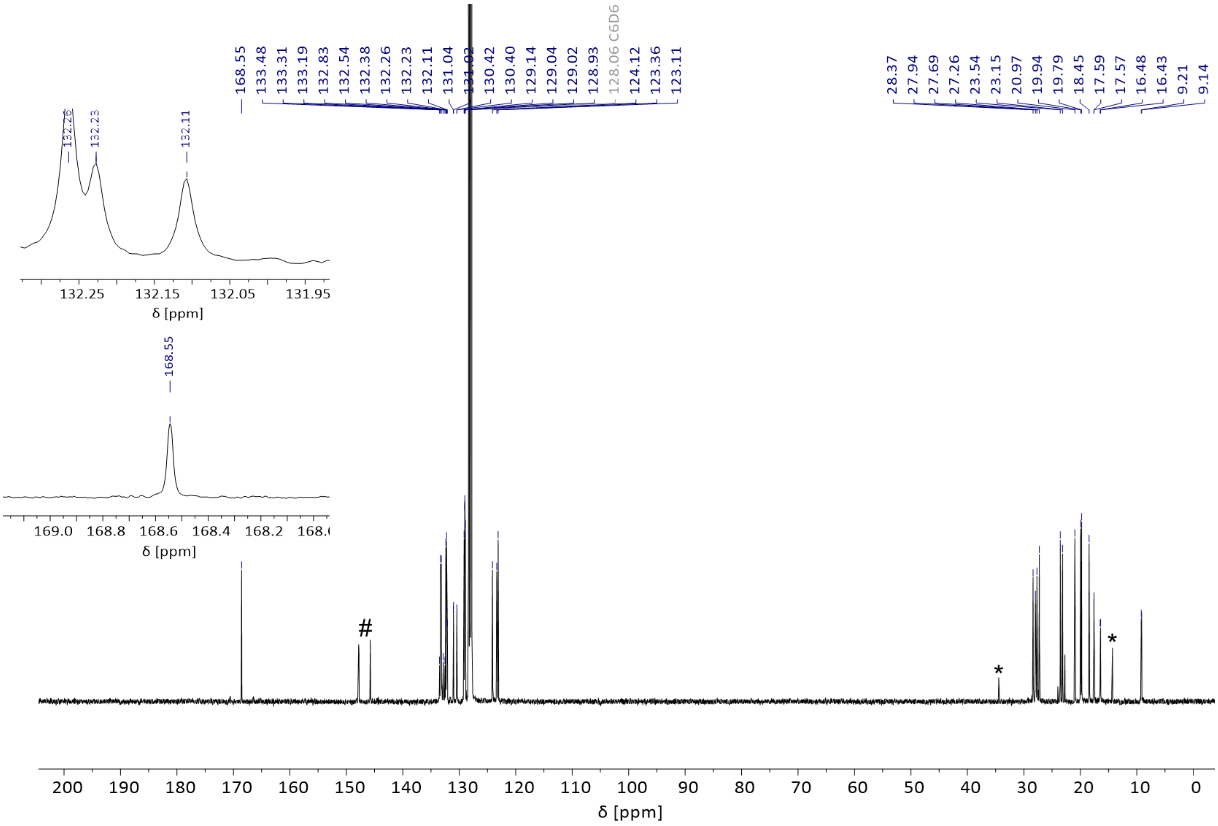

Figure S33. $^{13}C\{^1H\}$ NMR spectrum of **5a** as a solution in C_6D_6 at ambient temperature; * indicates small amounts of pentane, while # marks an unknown impurity.

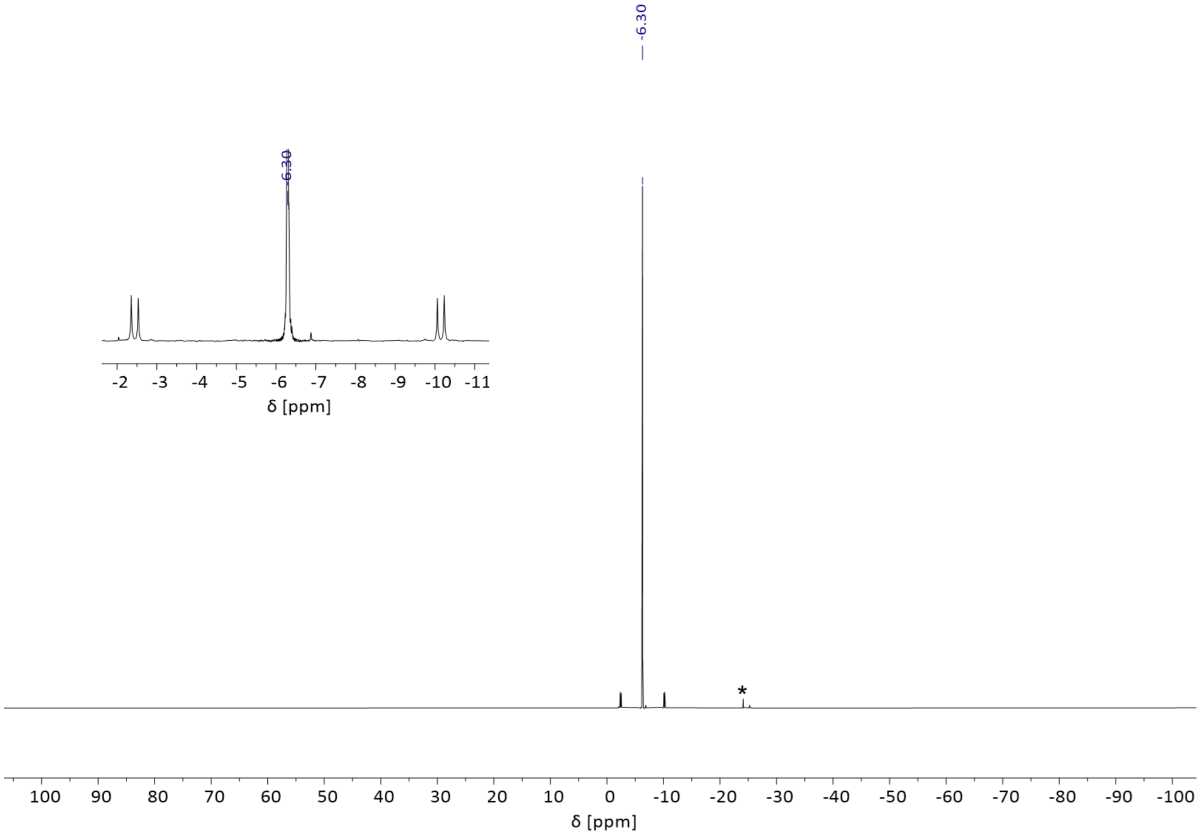

Figure S34. $^{31}P\{^1H\}$ NMR spectrum of **5a** as a solution in C_6D_6 at ambient temperature; * indicates small amounts of free ligand ^{Ph}LH.

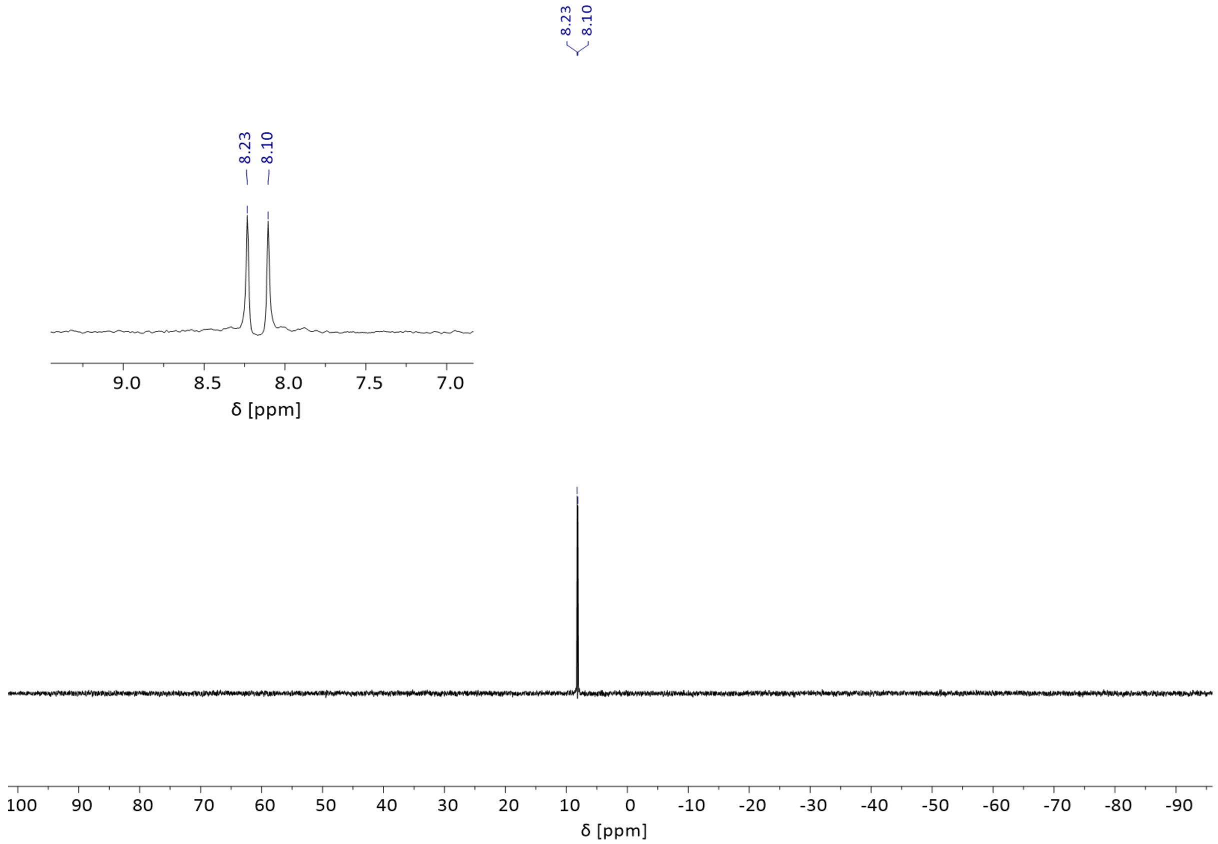

Figure S35. $^{29}Si\{^{1}H\}$ NMR spectrum of **5a** as a solution in C_6D_6 at ambient temperature.

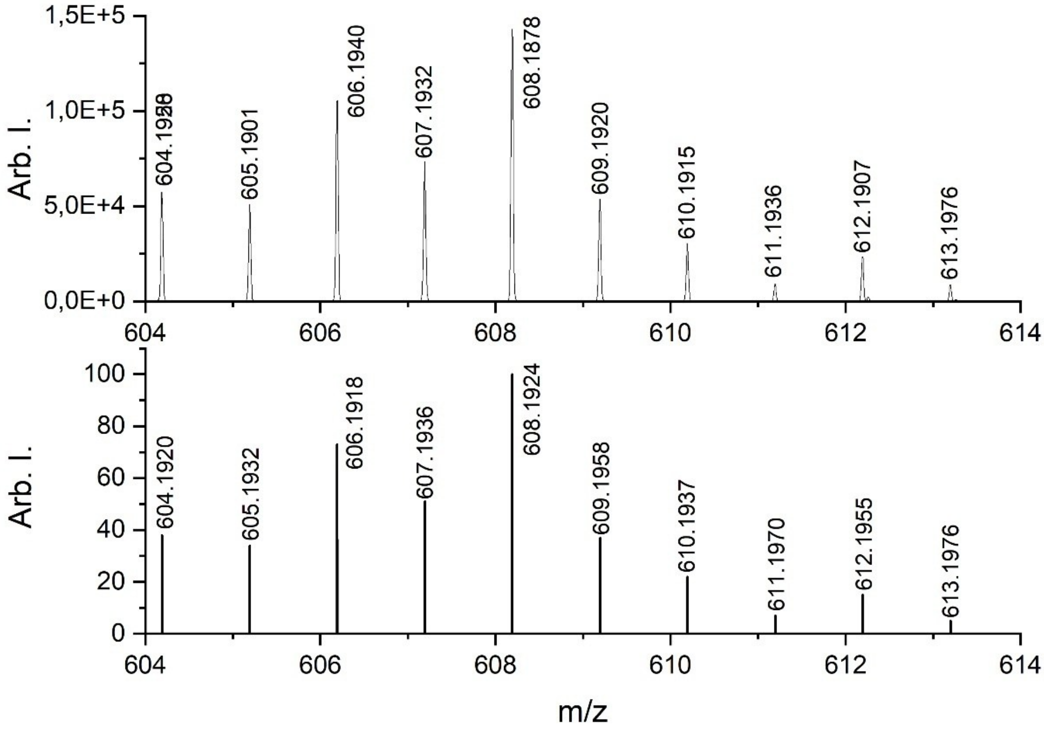

Figure S36. Cutout from LIFDI/MS of **5a**; **Top**: found MS for [M-C_2H_3]; **Bottom**: Calculated MS spectrum of [M-C_2H_3].

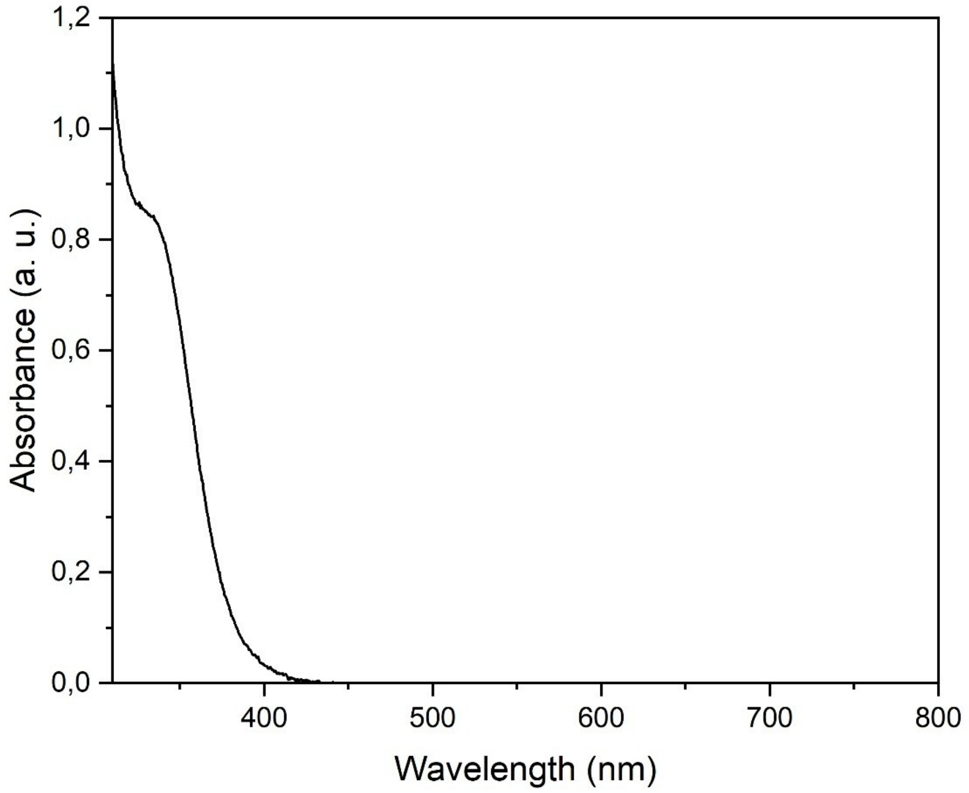

Figure S37. UV/Vis spectrum of a 2.5x10^{-4} M solution of **5a** in toluene at ambient temperature.

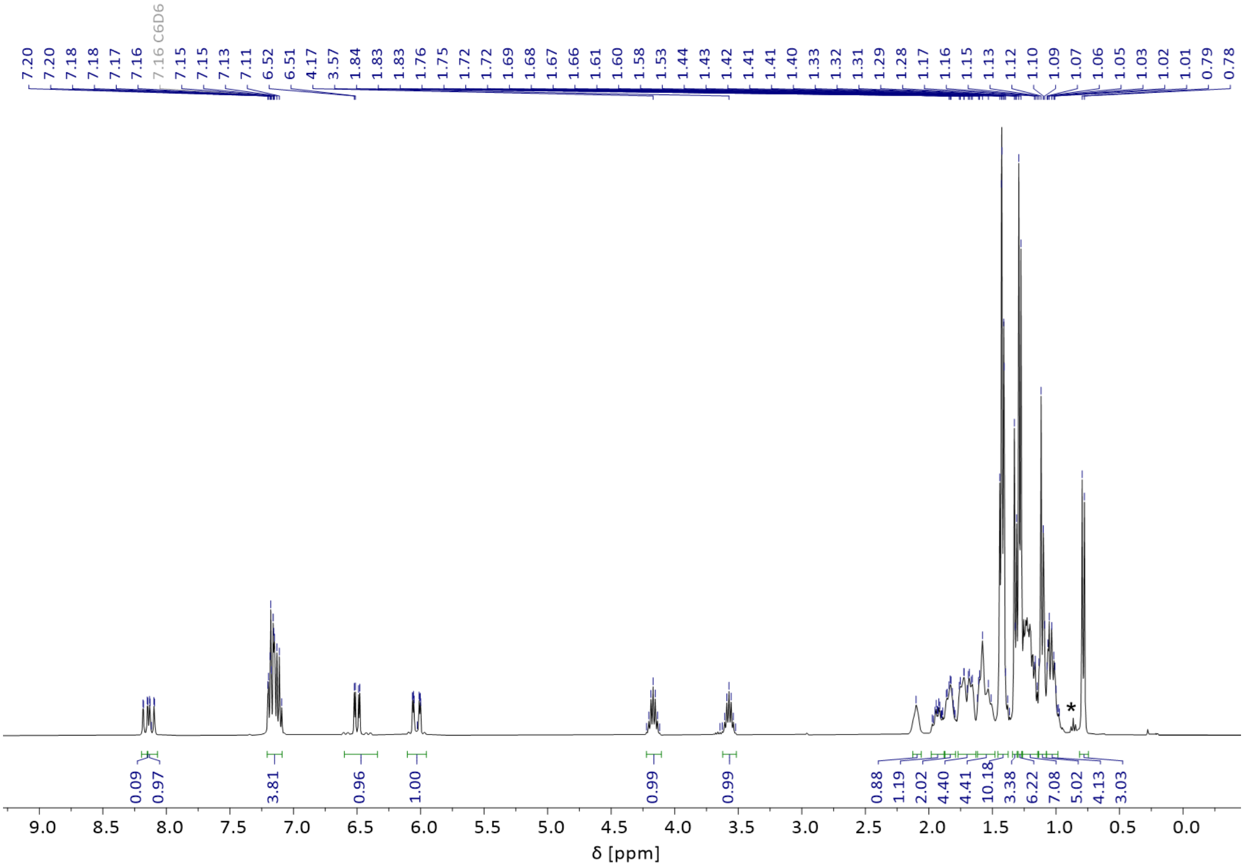

Figure S38. ^{1}H NMR spectrum of **5b** as a solution in C_6D_6 at ambient temperature; * indicates small amounts of pentane.

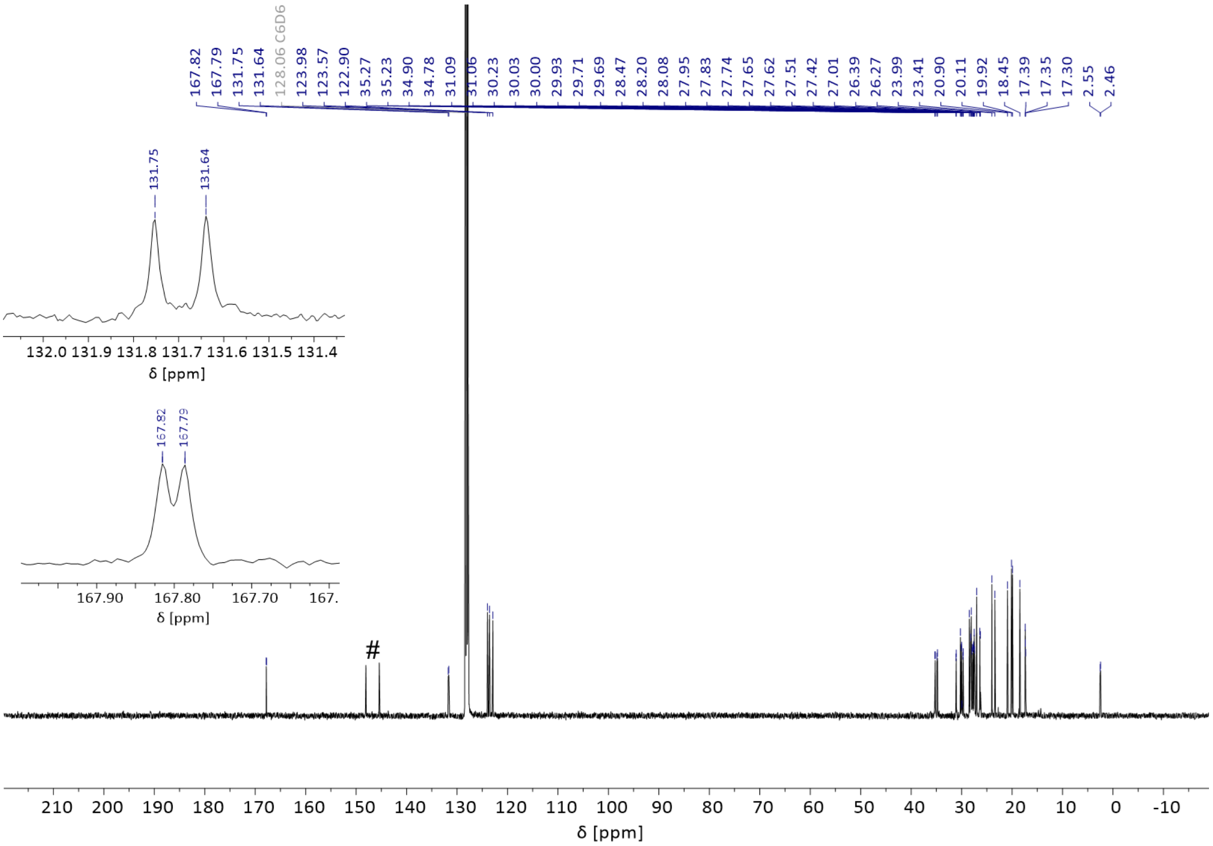

Figure S39. $^{13}C\{^{1}H\}$ NMR spectrum of **5b** as a solution in C_6D_6 at ambient temperature; * indicates small amounts of pentane, while # marks an unknown impurity.

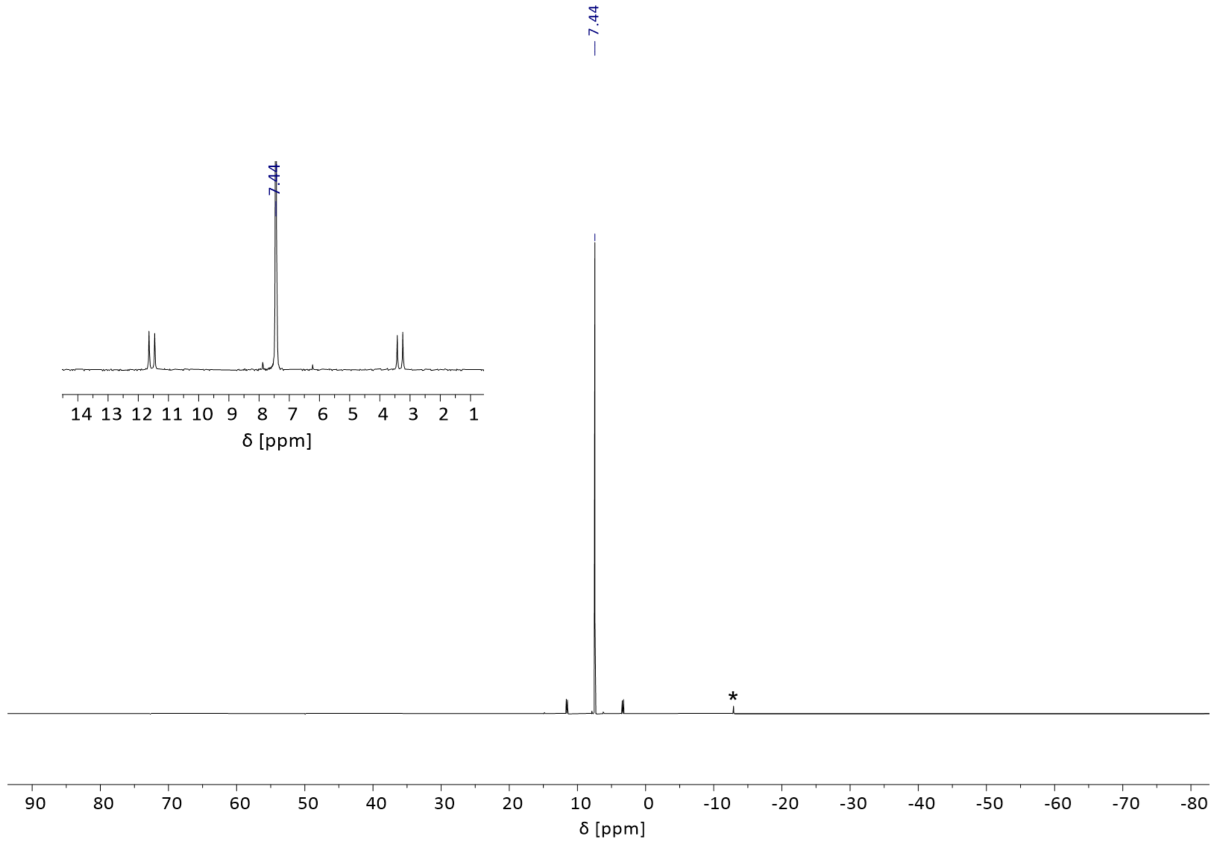

Figure S40. $^{31}P\{^{1}H\}$ NMR spectrum of **5b** as a solution in C_6D_6 at ambient temperature; * indicates small amounts of free ligand ^{Cy}LH.

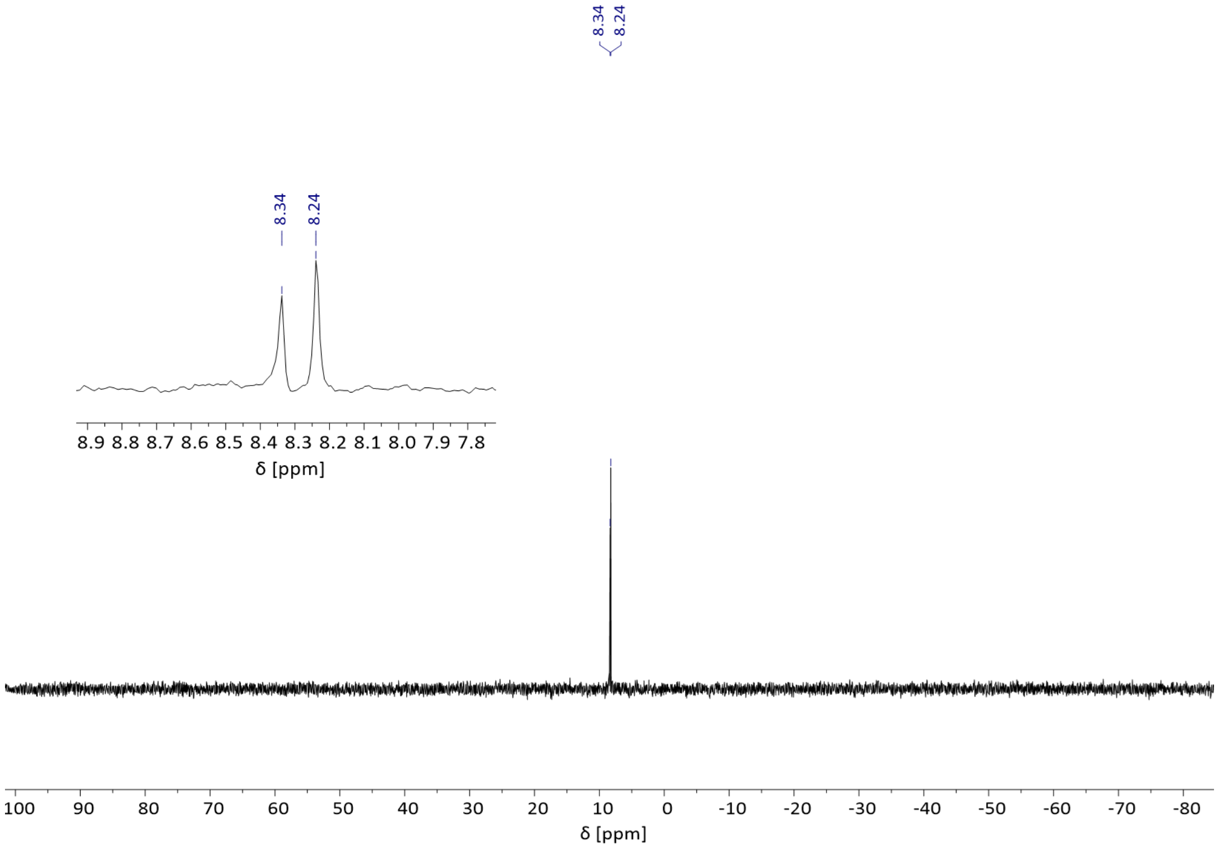

Figure S41. $^{29}Si\{^1H\}$ NMR spectrum of **5b** as a solution in C_6D_6 at ambient temperature.

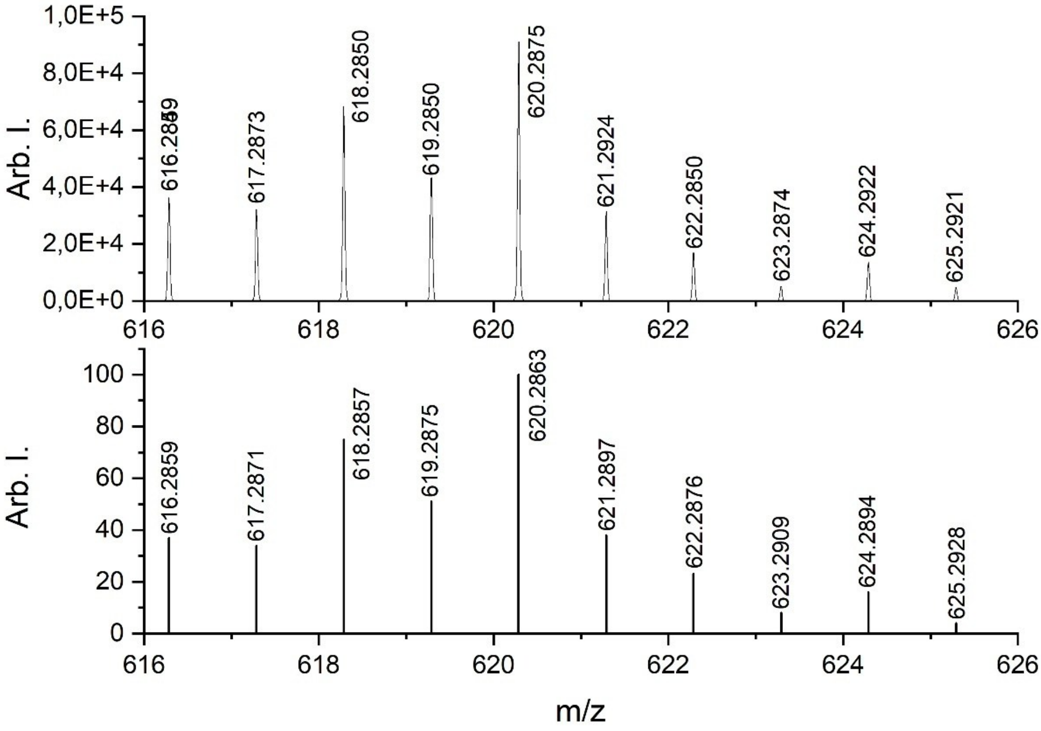

Figure S42. Cutout from LIFDI/MS of **5b**; **Top**: found MS for [M-C_2H_3]; **Bottom**: Calculated MS spectrum of [M-C_2H_3].

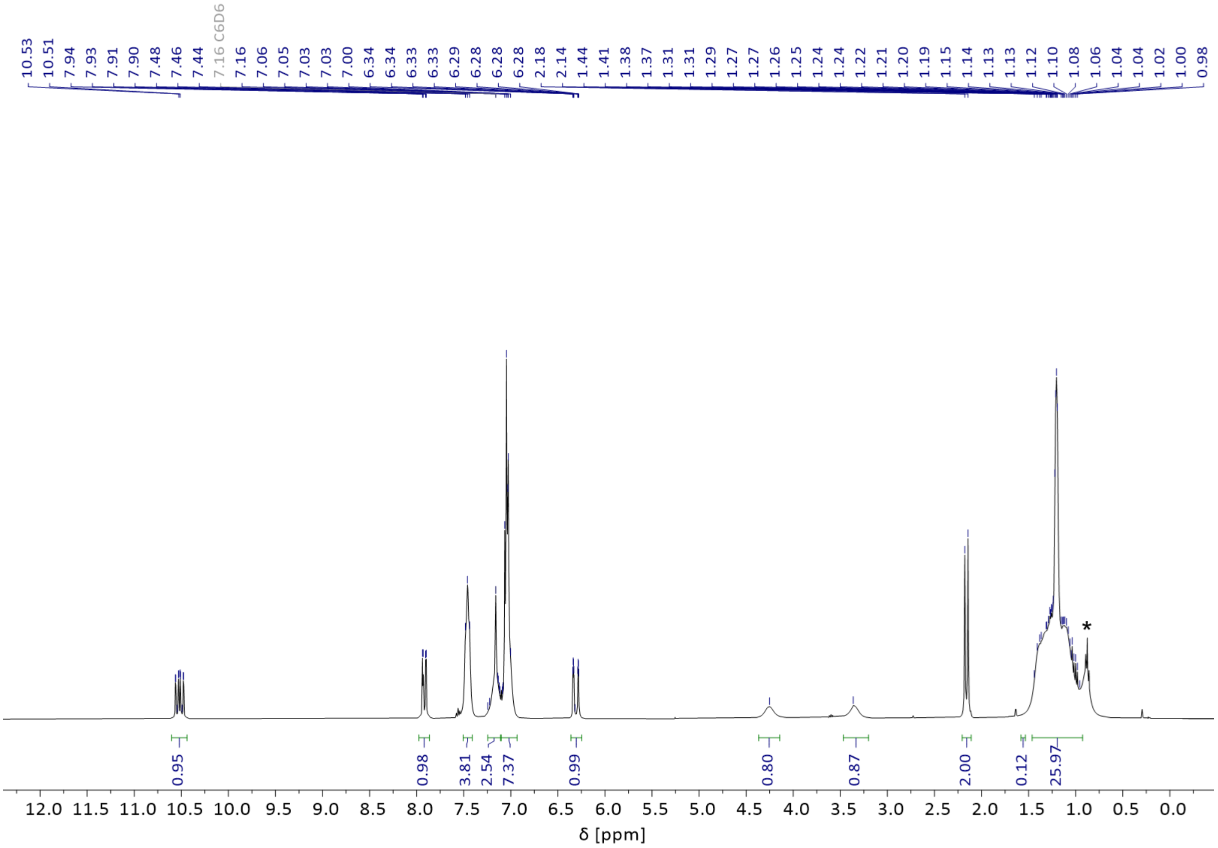

Figure S43. ^{1}H NMR spectrum of **6a** as a solution in C_6D_6 at ambient temperature; * indicates small amounts of pentane.

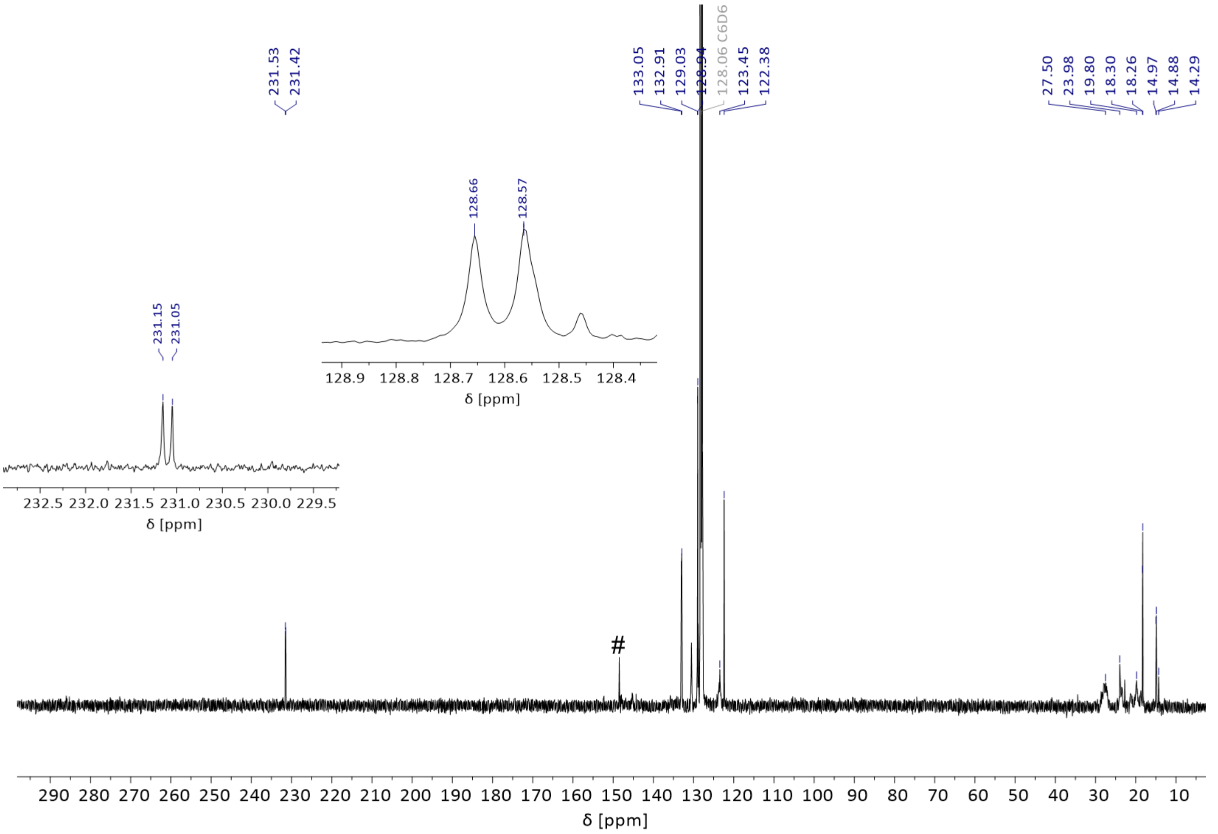

Figure S44. $^{13}C\{^1H\}$ NMR spectrum of **6a** as a solution in C_6D_6 at ambient temperature; # marks an unknown impurity.

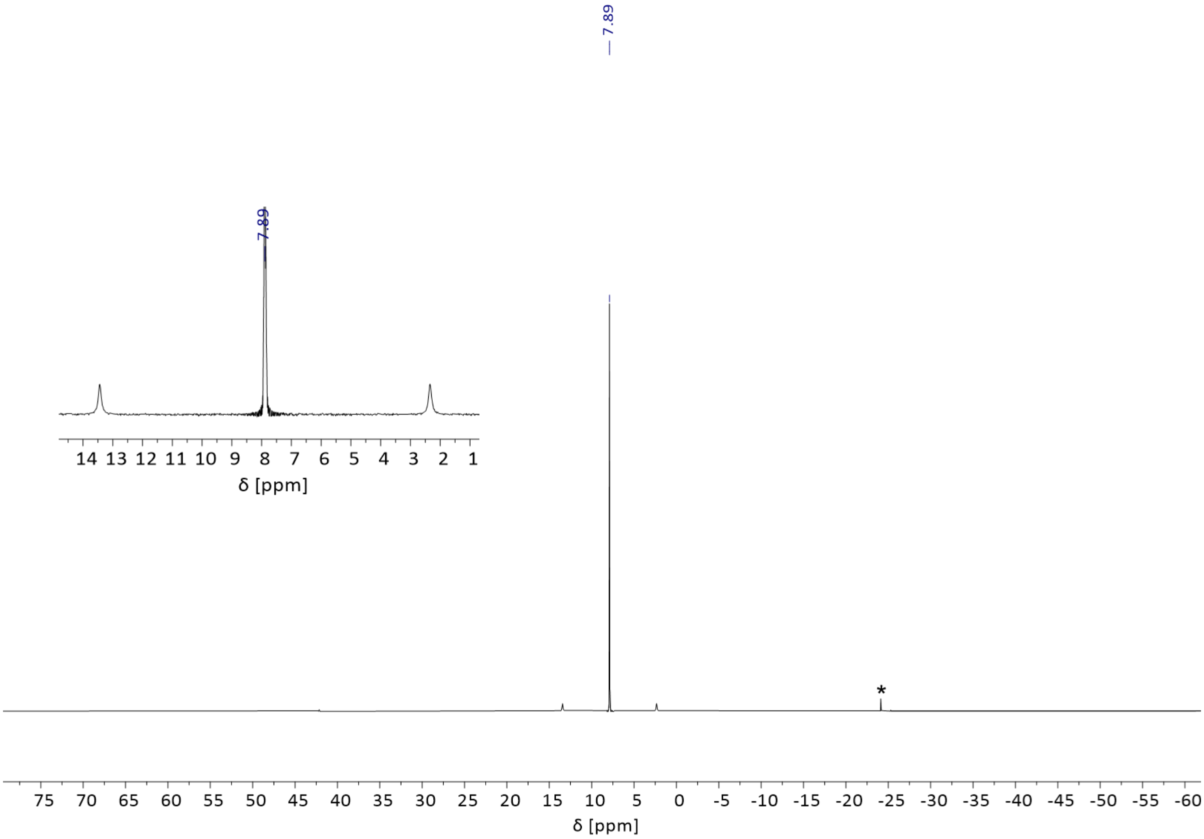

Figure S45. $^{31}P\{^1H\}$ NMR spectrum of **6a** as a solution in C_6D_6 at ambient temperature; * indicates small amounts of free ligand ^{Ph}LH.

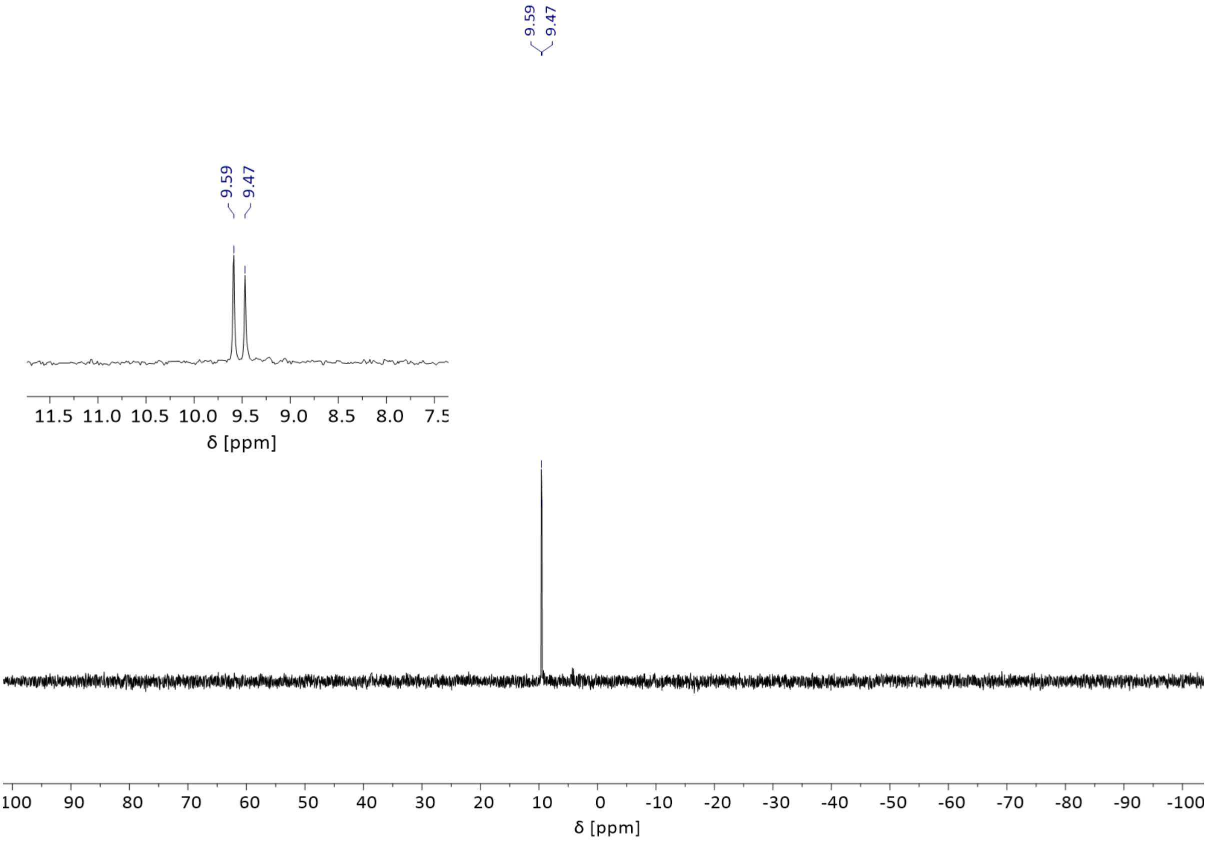

Figure S46. $^{29}Si\{^1H\}$ NMR spectrum of **6a** as a solution in C_6D_6 at ambient temperature.

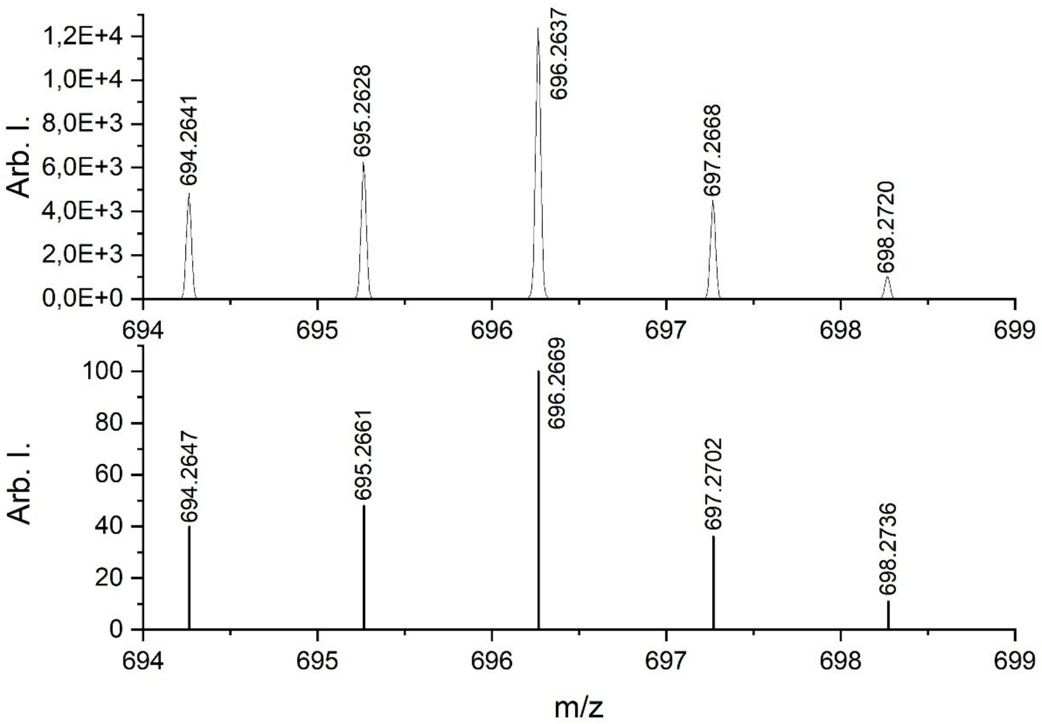

Figure S47. Cutout from LIFDI/MS of **6a**; **Top**: found MS for [M-C_2H_3]; **Bottom**: Calculated MS spectrum of [M-C_2H_3].

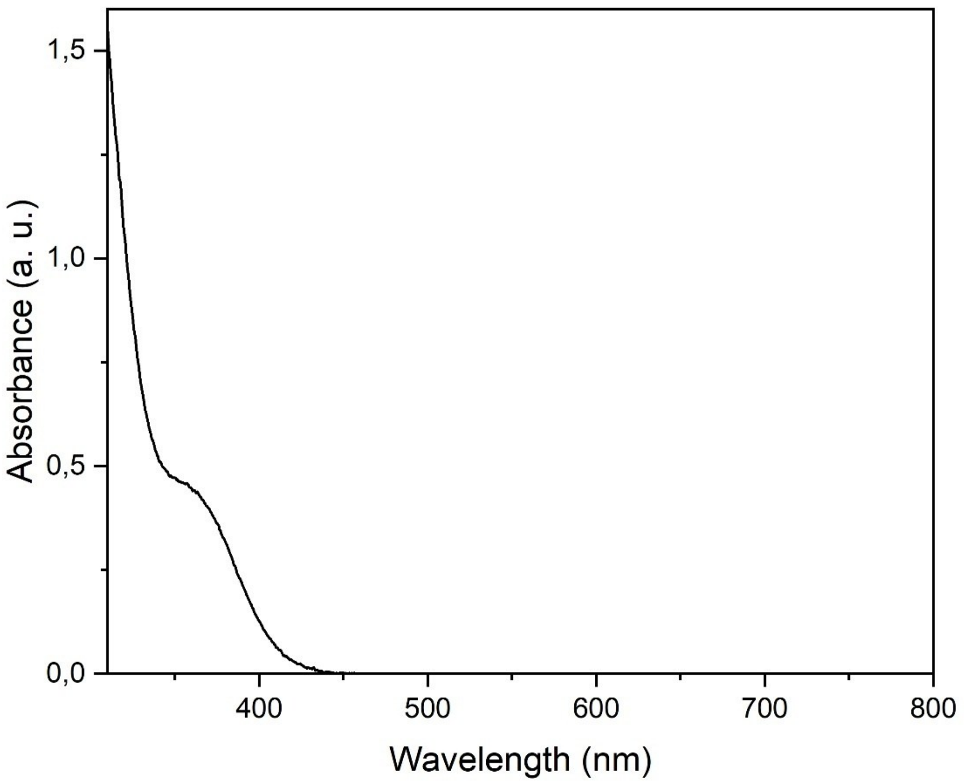

Figure S48. UV/Vis spectrum of a 5x10^{-4} M solution of **6a** in toluene at ambient temperature.

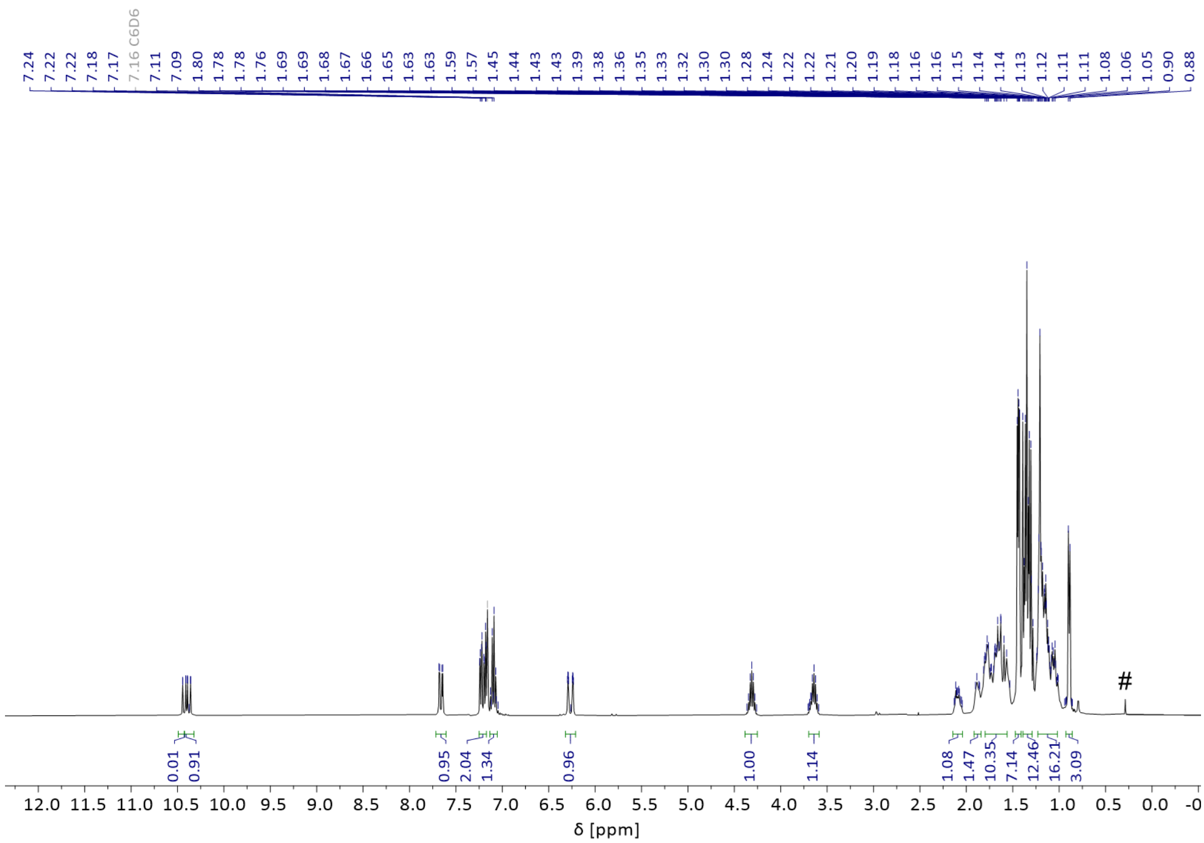

Figure S49. ^{1}H NMR spectrum of **6b** as a solution in C_6D_6 at ambient temperature; # marks an unknown impurity.

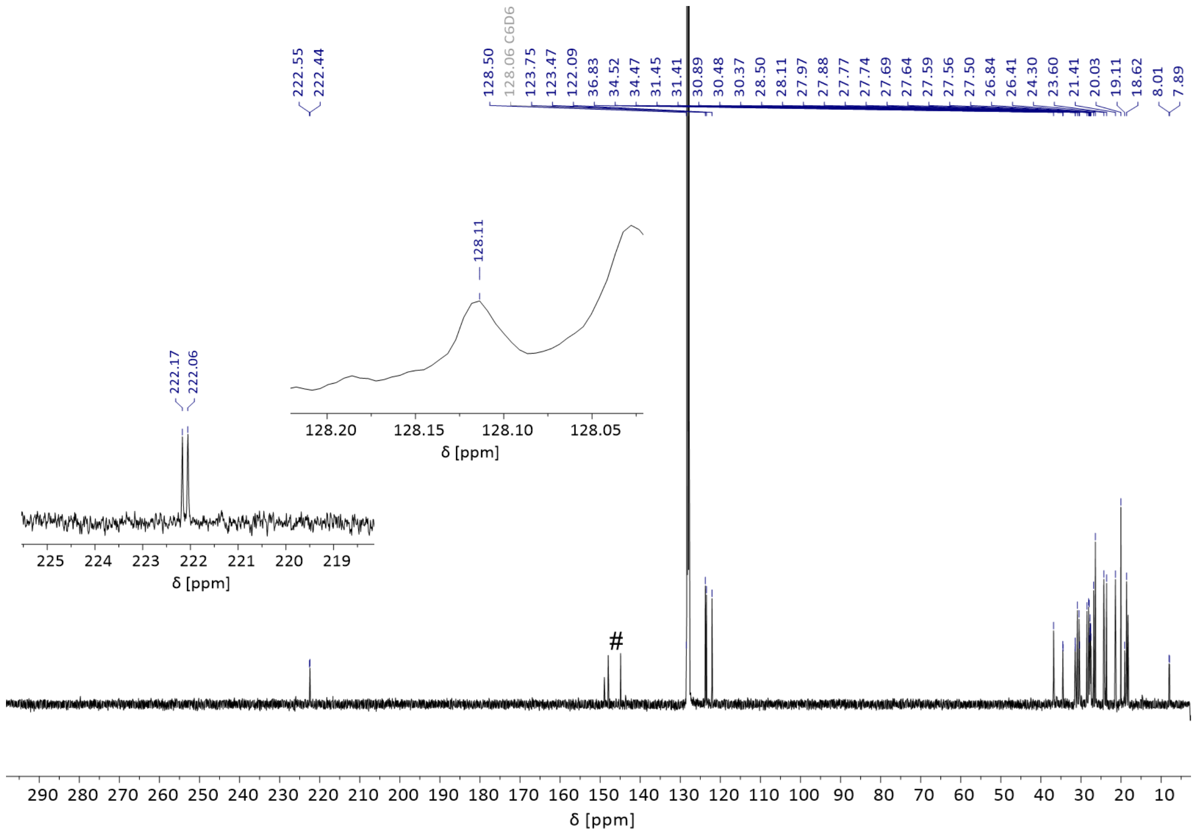

Figure S50. $^{13}C\{^{1}H\}$ NMR spectrum of **6b** as a solution in C_6D_6 at ambient temperature; # marks an unknown impurity.

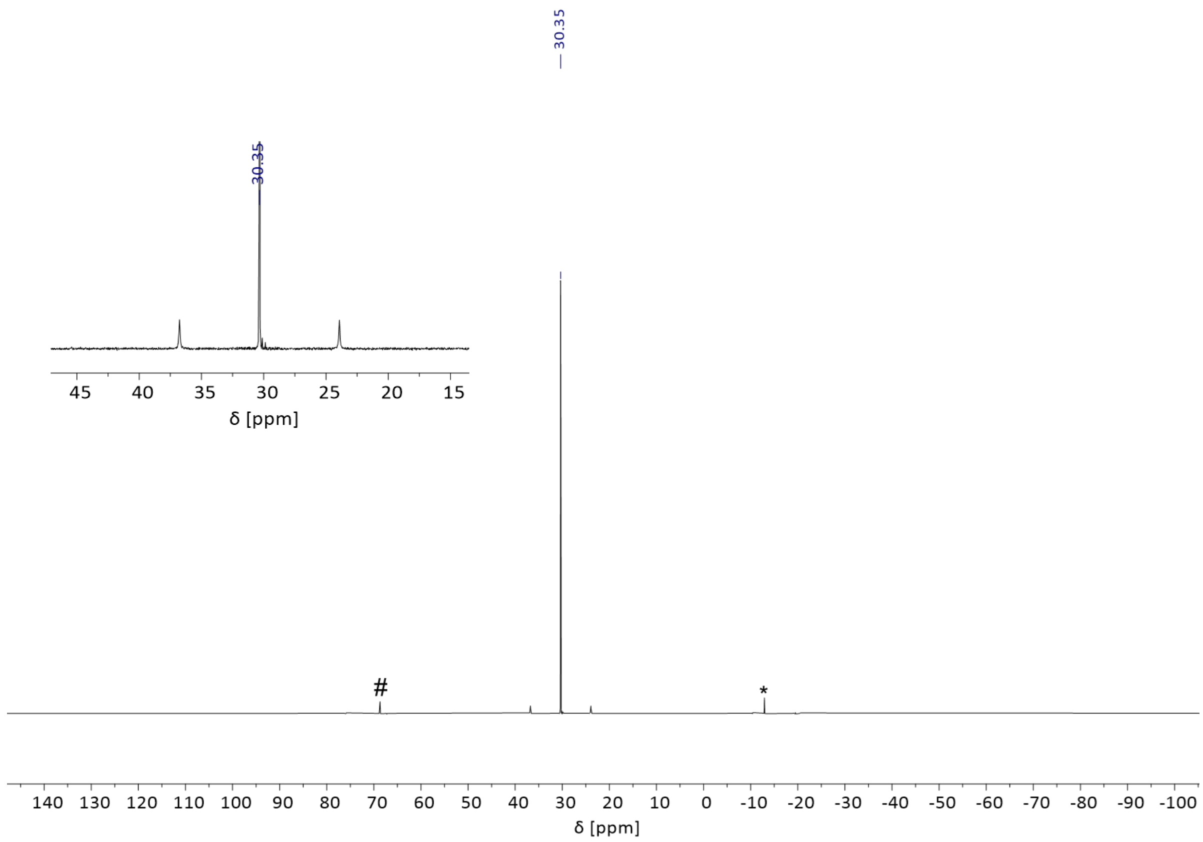

Figure S51. $^{31}P\{^{1}H\}$ NMR spectrum of **6b** as a solution in C_6D_6 at ambient temperature; * indicates small amounts of free ligand ^{Cy}LH, while # marks an unknown impurity.

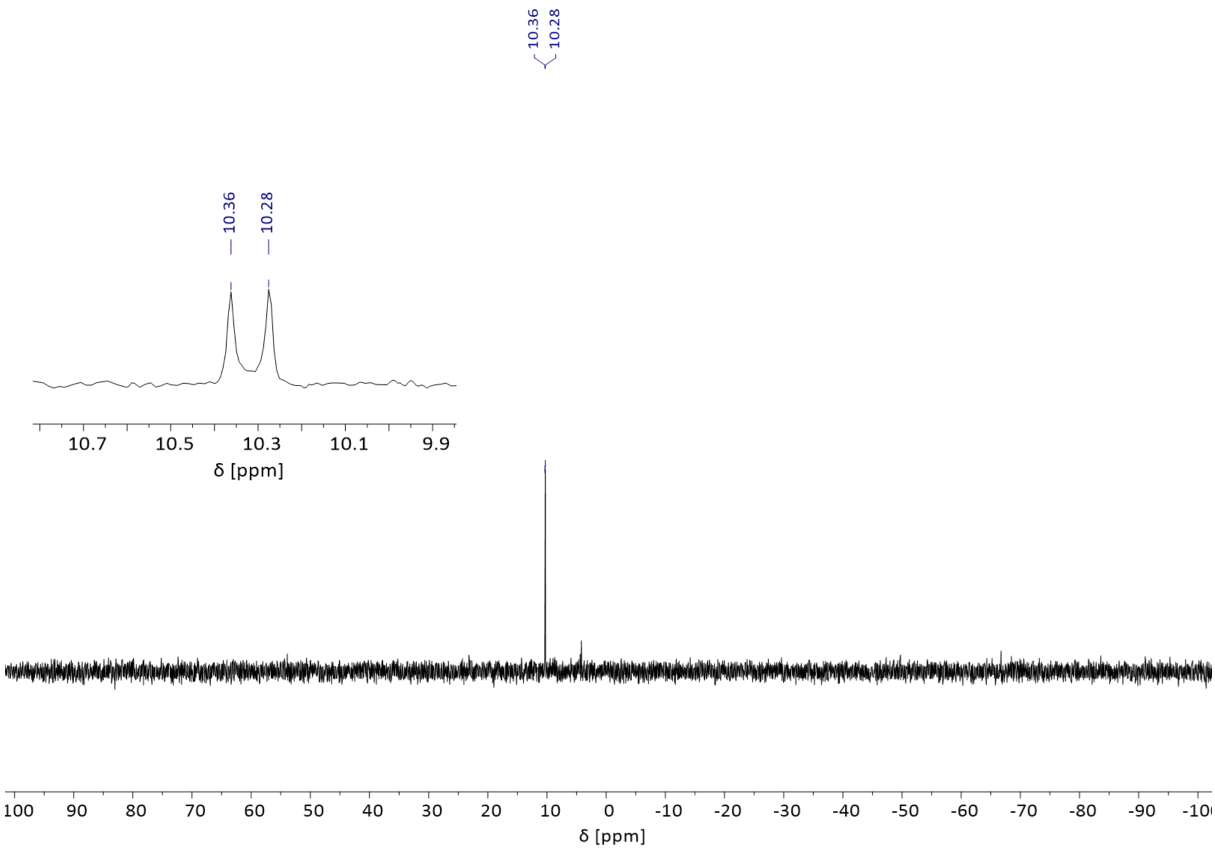

Figure S52. $^{29}Si\{^{1}H\}$ NMR spectrum of **6b** as a solution in C_6D_6 at ambient temperature.

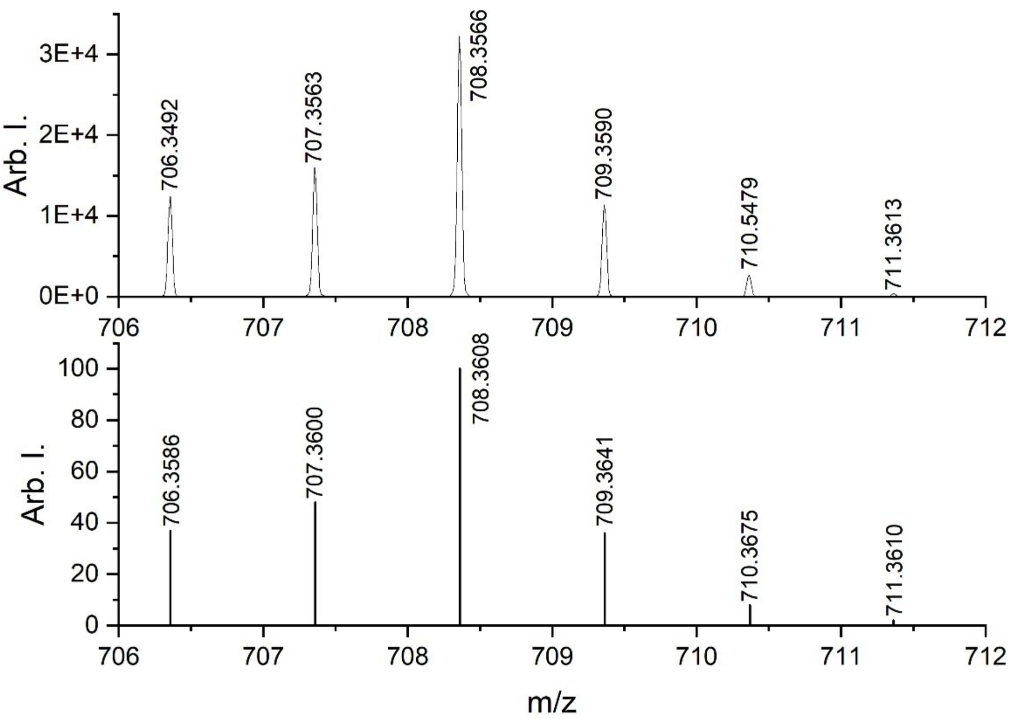

Figure S53. Cutout from LIFDI/MS of **6b**; **Top**: found MS for [M-C_2H_3]; **Bottom**: Calculated MS spectrum of [M-C_2H_3].

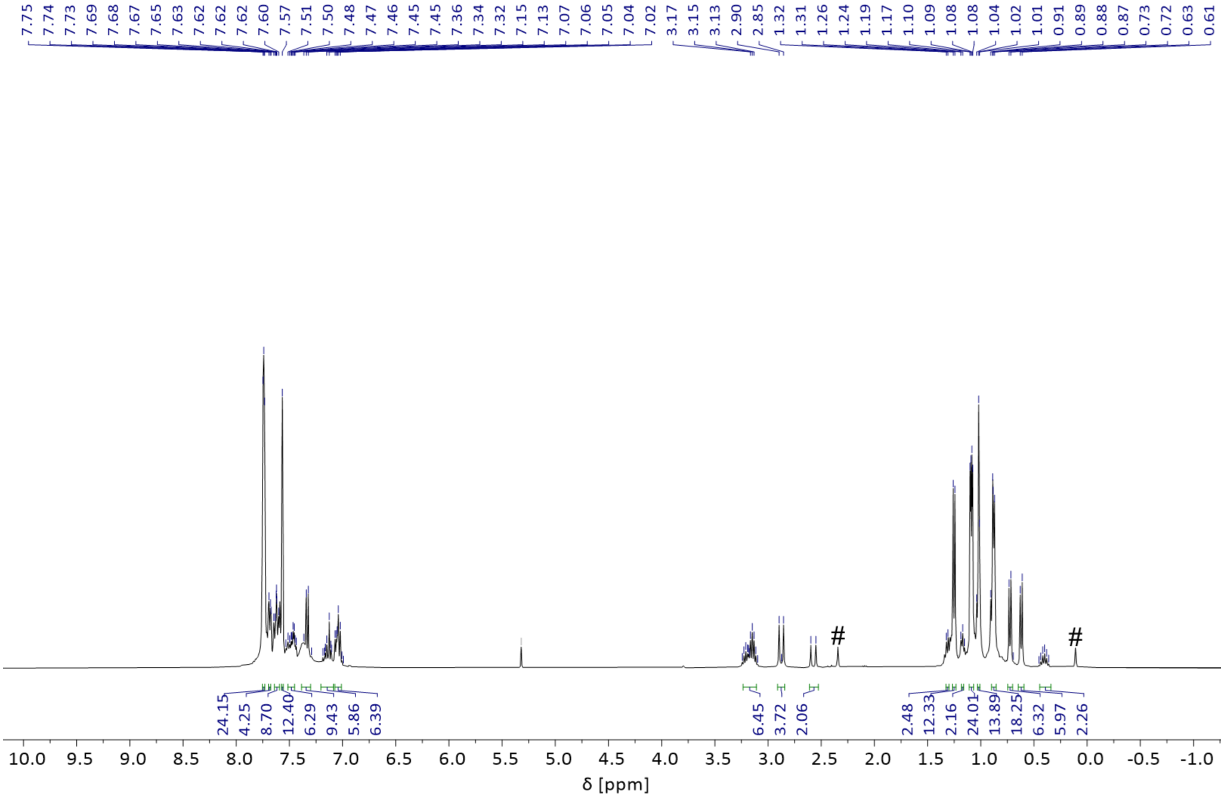

Figure S54. ^{1}H NMR spectrum of **7** as a solution in CD_2Cl_2 at ambient temperature; # marks an unknown impurity.

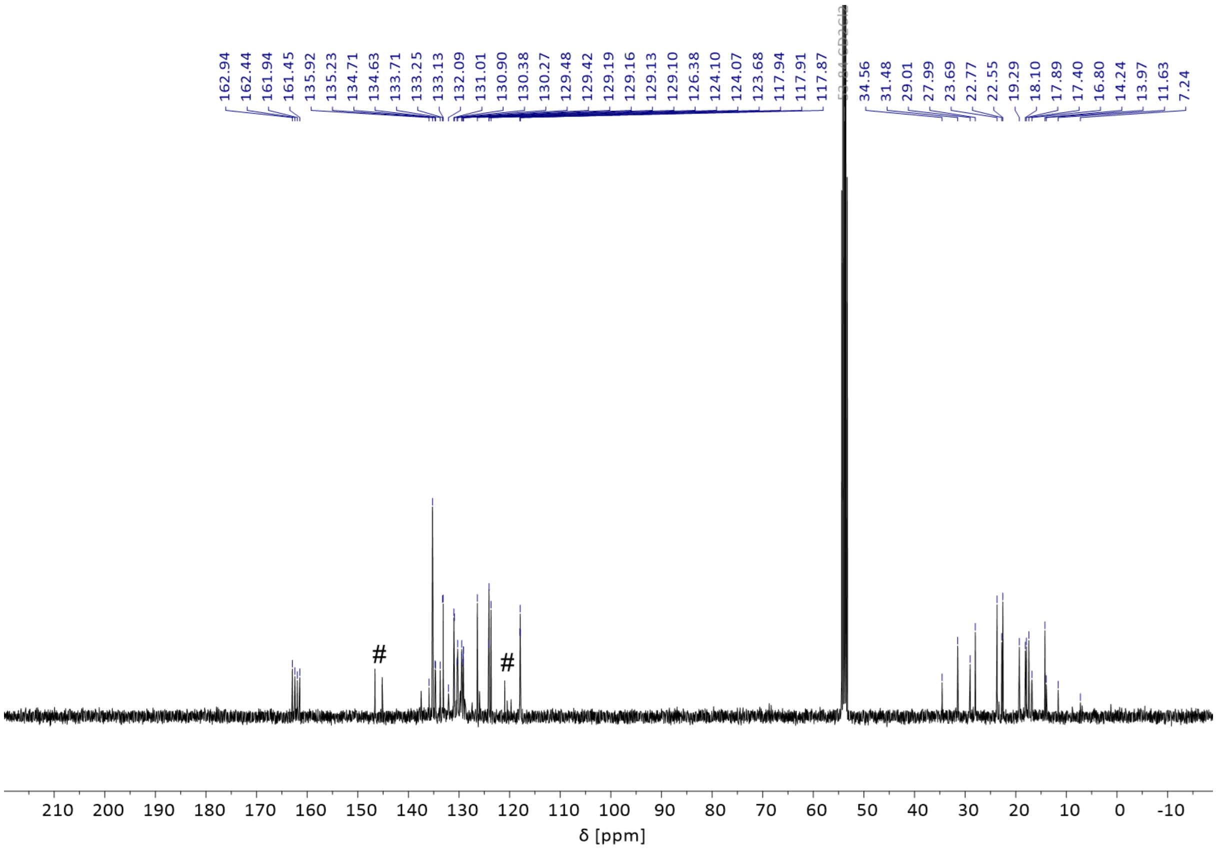

Figure S55. $^{13}C\{^1H\}$ NMR spectrum of **7** as a solution in CD_2Cl_2 at ambient temperature; # marks an unknown impurity.

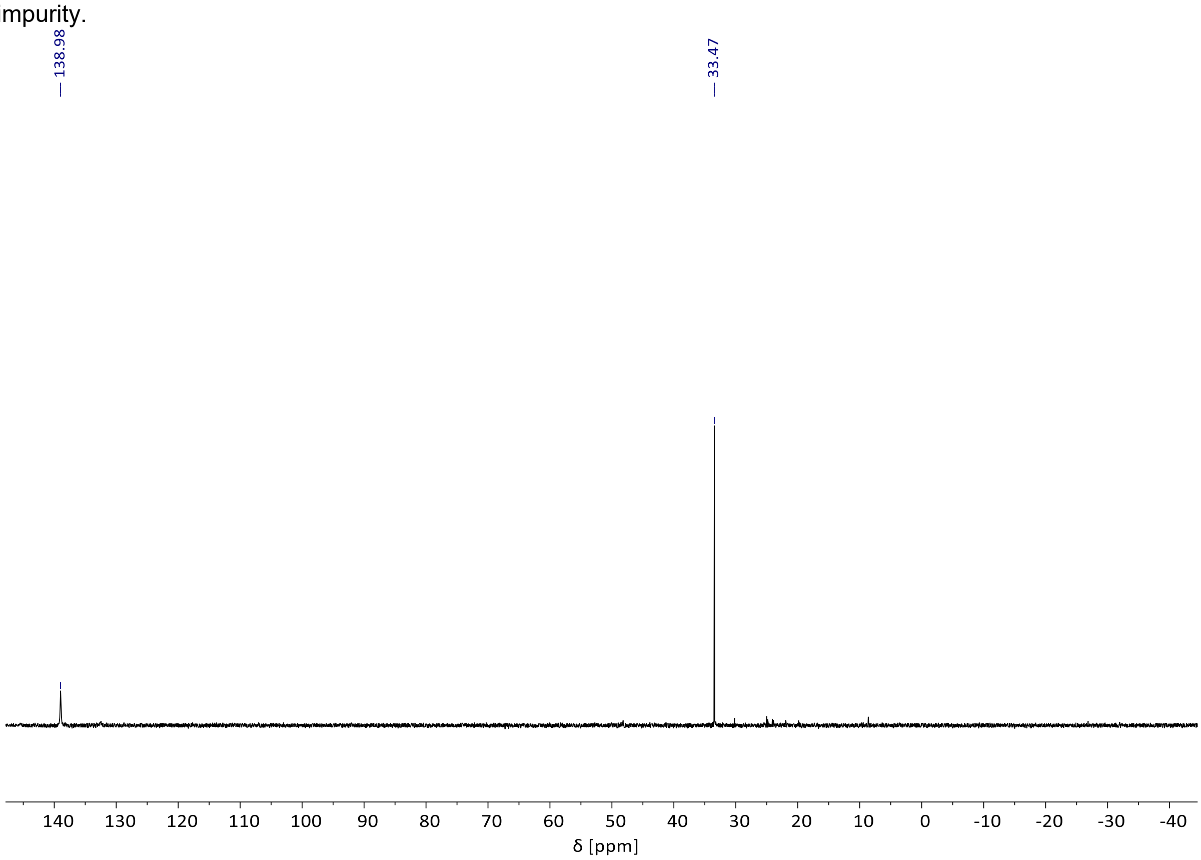

Figure S56. $^{31}P\{^1H\}$ NMR spectrum of **7** as a solution in CD_2Cl_2 at ambient temperature.

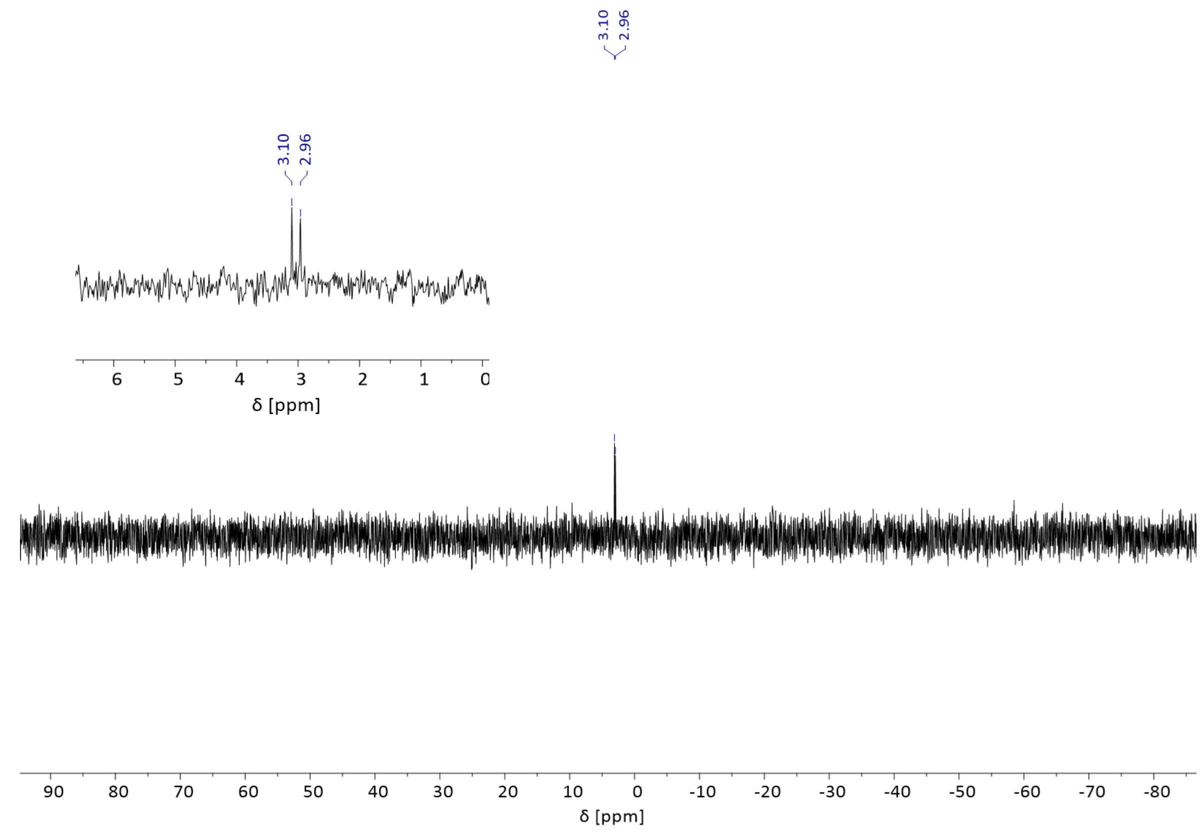

Figure S57. $^{29}Si\{^{1}H\}$ NMR spectrum of **7** as a solution in CD_2Cl_2 at ambient temperature.

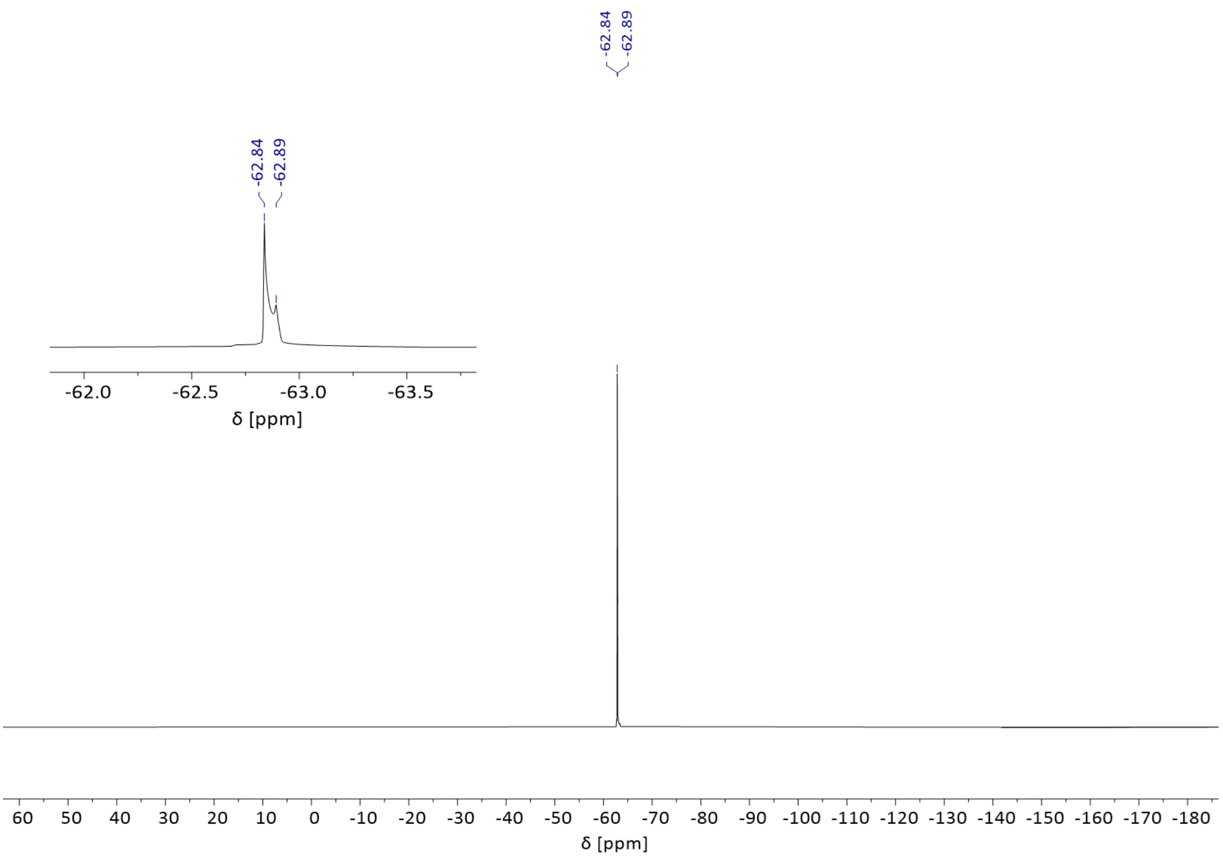

Figure S58. $^{19}F\{^{1}H\}$ NMR spectrum of **7** as a solution in CD_2Cl_2 at ambient temperature.

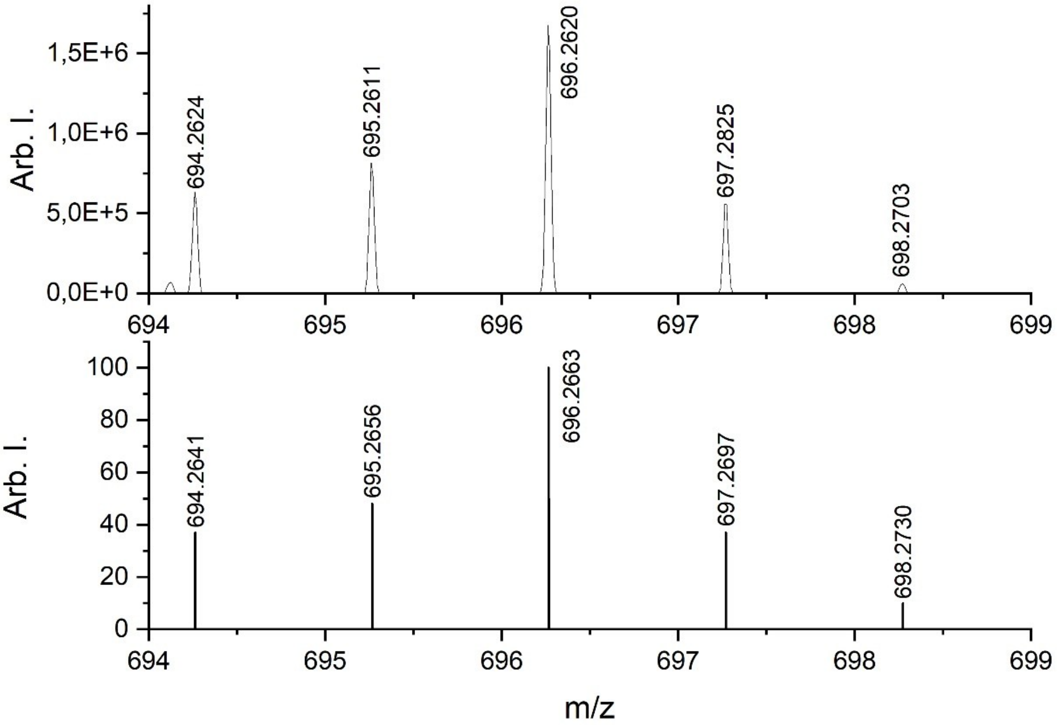

Figure S59. Cutout from LIFDI/MS of **7**; **Top**: found MS for [M-BAr$^F{}_4$]$^+$; **Bottom**: Calculated MS spectrum of [M-BAr$^F{}_4$]$^+$.

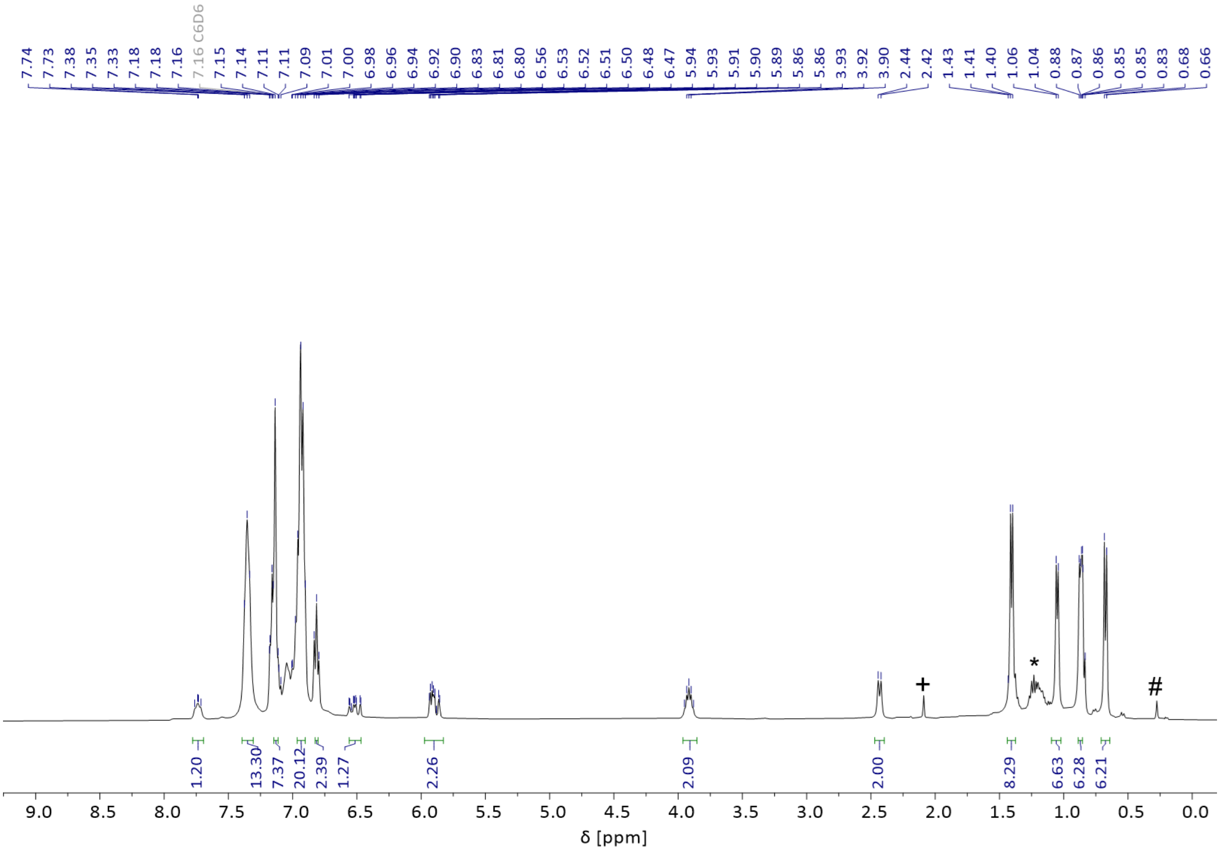

Figure S60. ^{1}H NMR spectrum of **8** as a solution in C_6D_6 at ambient temperature; * indicates small amounts of pentene, + of toluene, while # marks an unknown impurity.

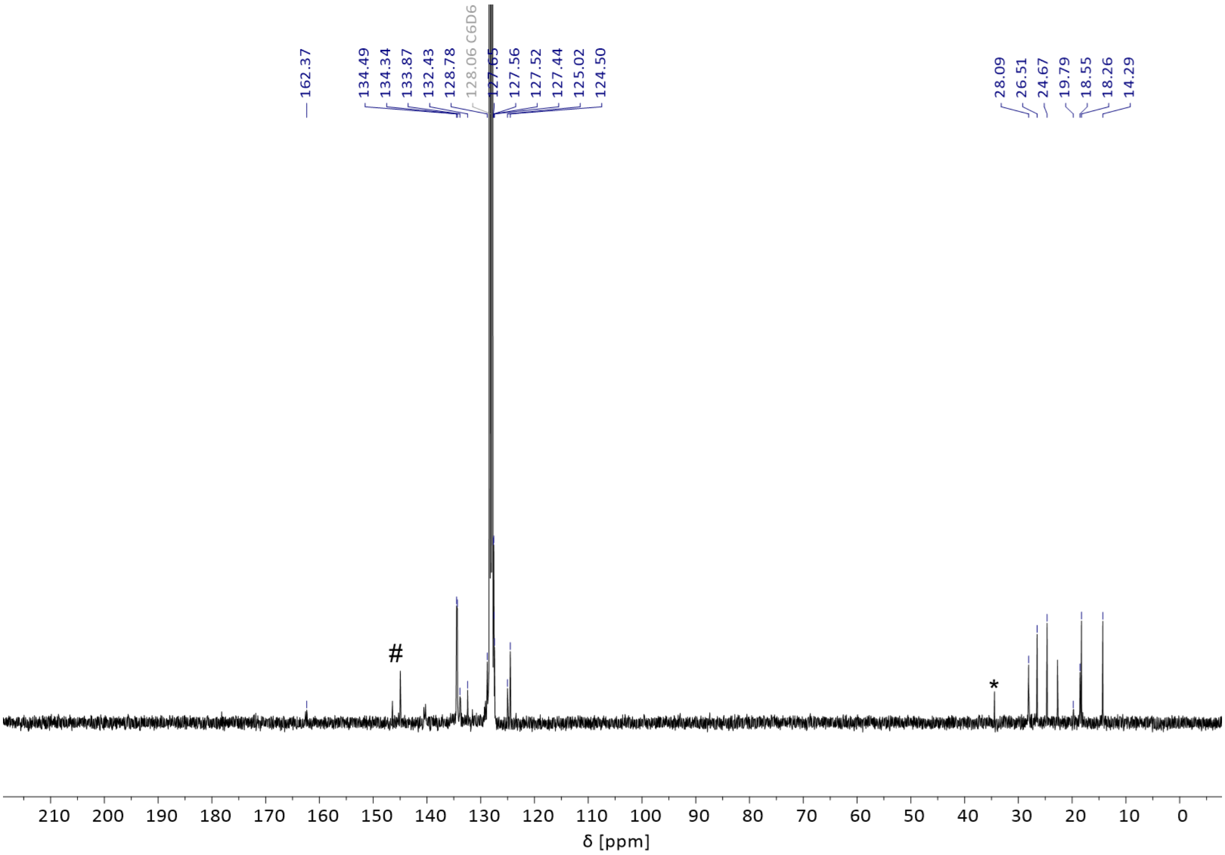

Figure S61. $^{13}C\{^1H\}$ NMR spectrum of **8** as a solution in C_6D_6 at ambient temperature; * indicates small amounts of pentane, while # marks an unknown impurity.

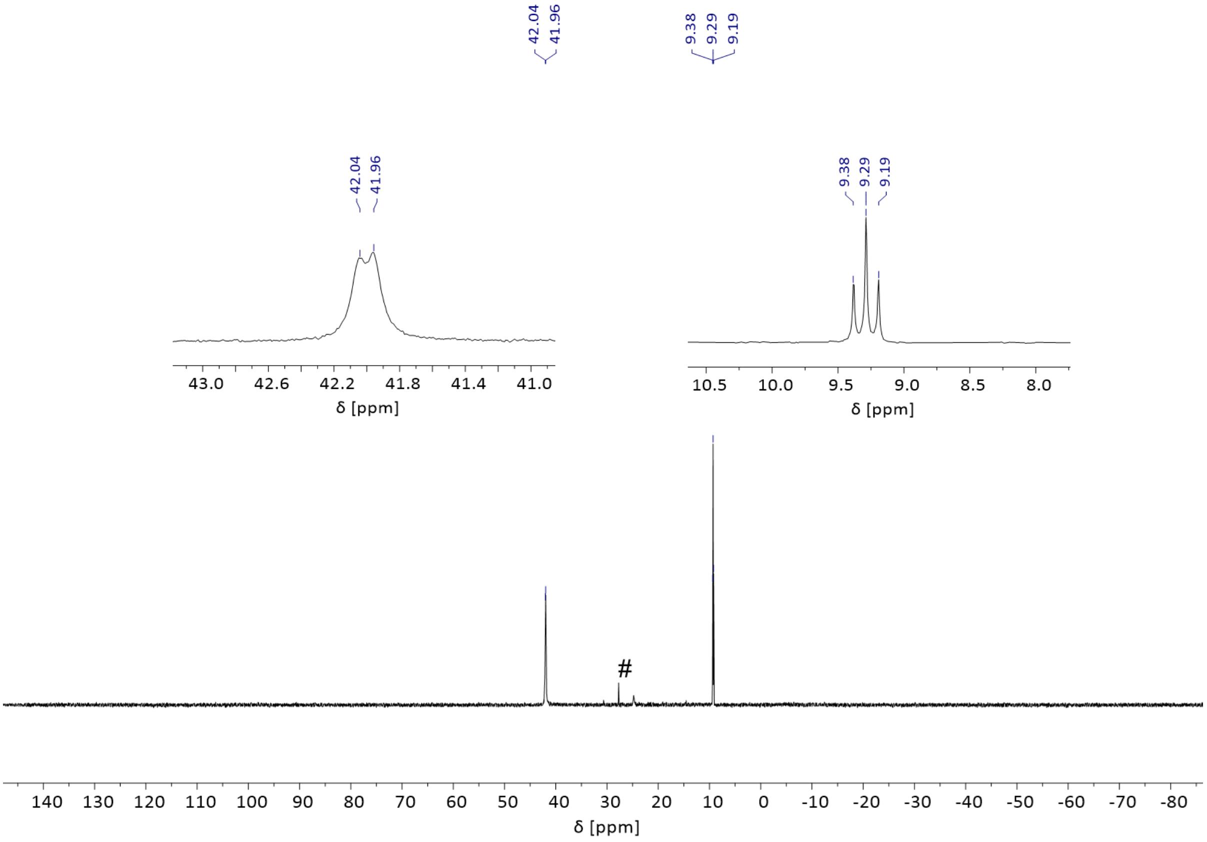

Figure S62. $^{31}P\{^1H\}$ NMR spectrum of **8** as a solution in C_6D_6 at ambient temperature; # marks an unknown impurity.

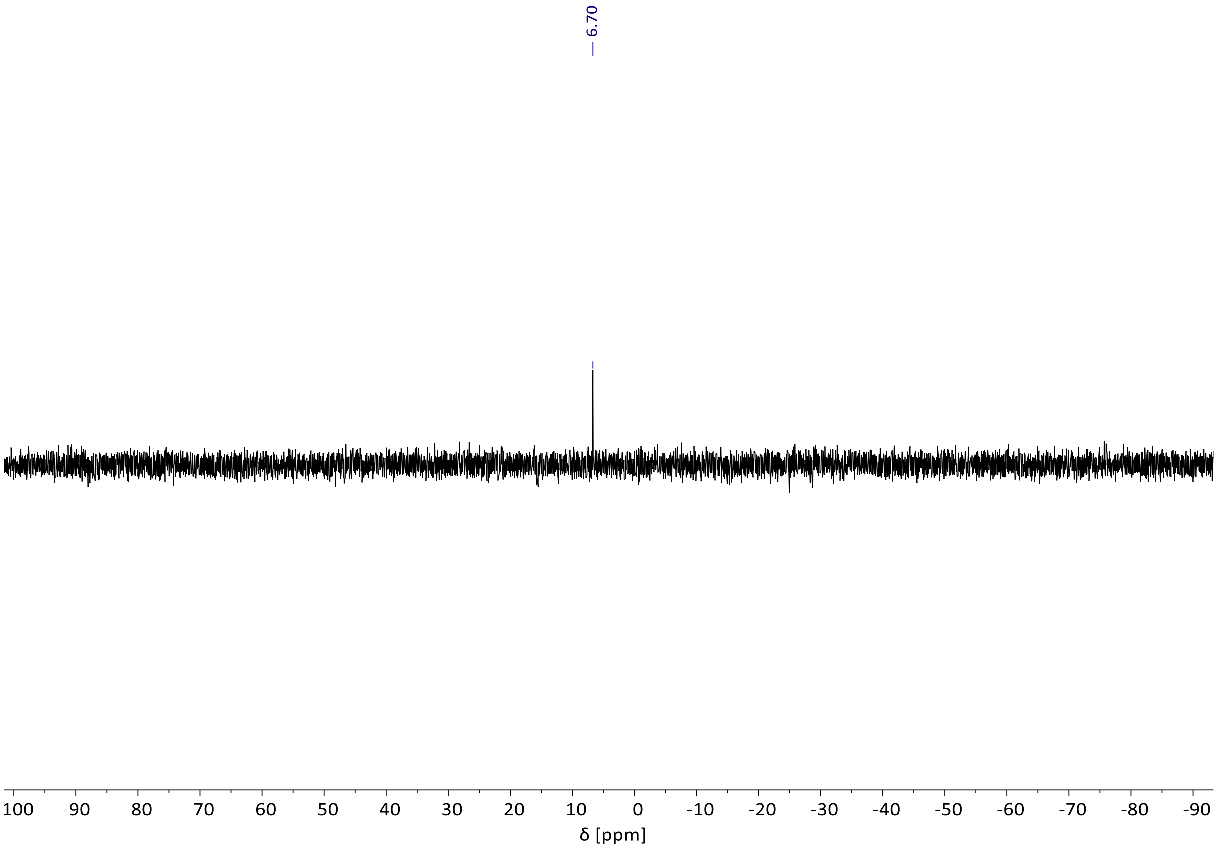

Figure S63. $^{29}Si\{^{1}H\}$ NMR spectrum of **8** as a solution in C_6D_6 at ambient temperature.

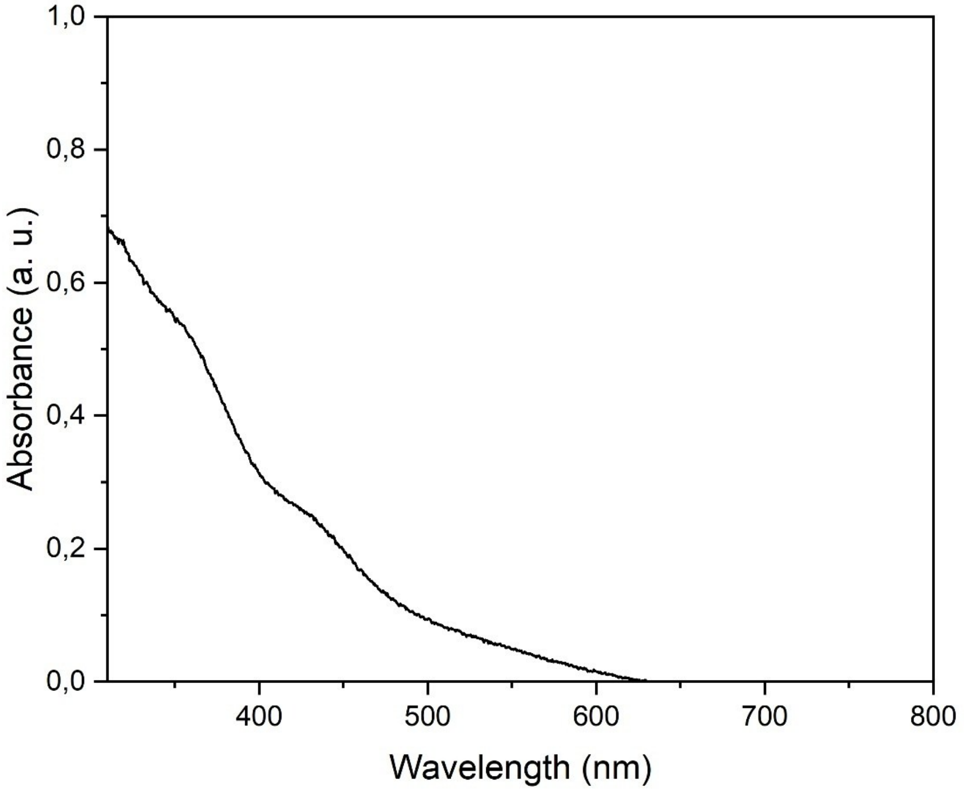

Figure S64. UV/Vis spectrum of a $5x10^{-5}$ M solution of **8** in toluene at ambient temperature.

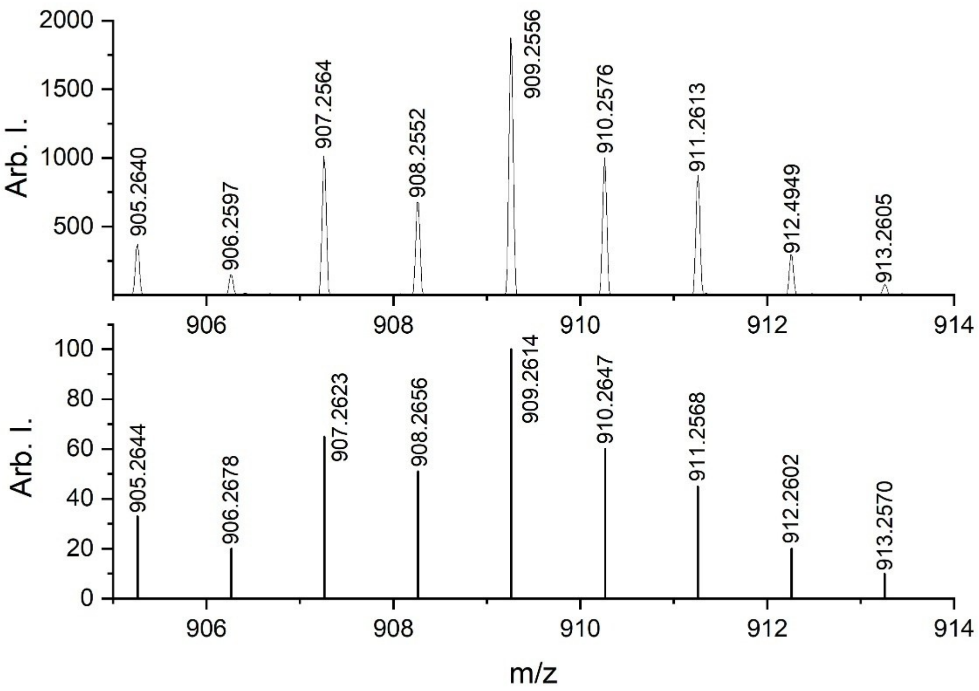

Figure S65. Cutout from LIFDI/MS of **8**; **Top**: found MS for [M-PPh_3]; **Bottom**: Calculated MS spectrum of [M-PPh_3].

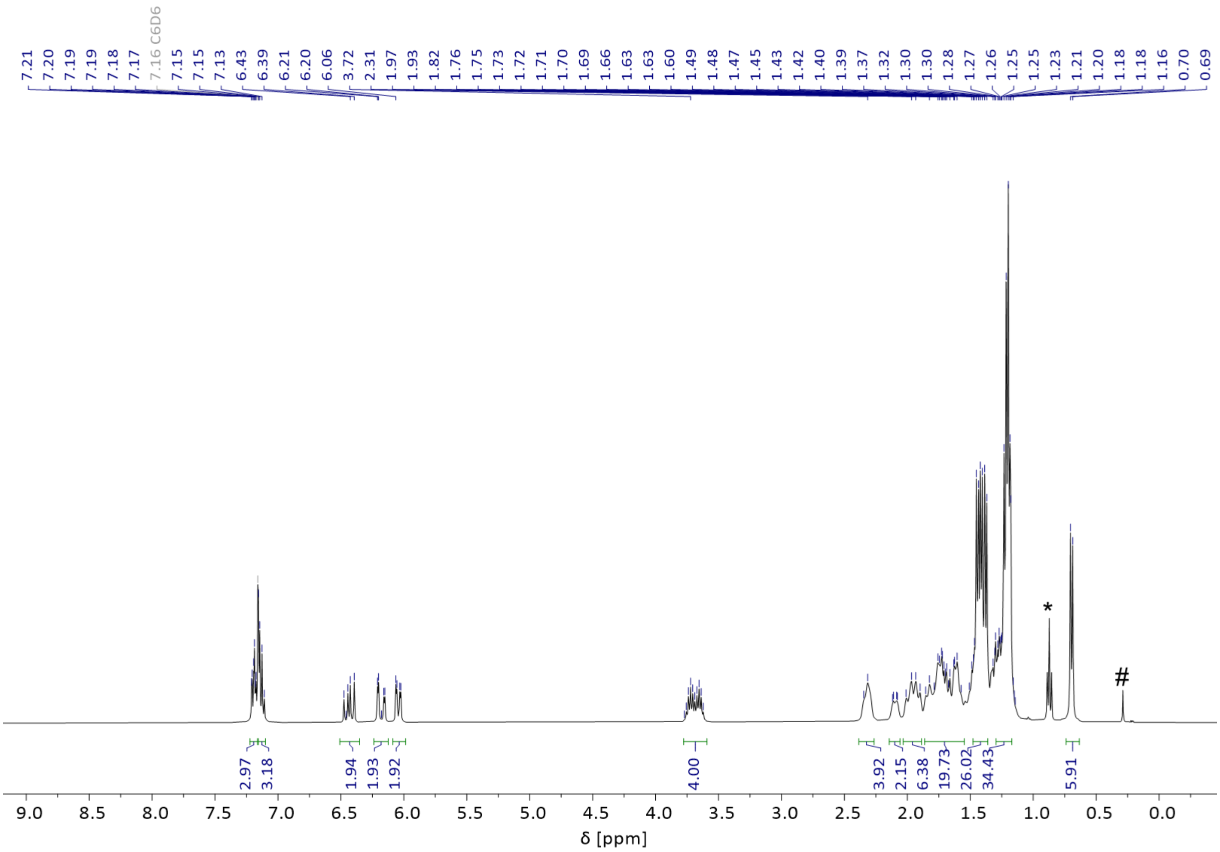

Figure S66. ^{1}H NMR spectrum of **9** as a solution in C_6D_6 at ambient temperature; * indicates small amounts of pentane, while # marks an unknown impurity.

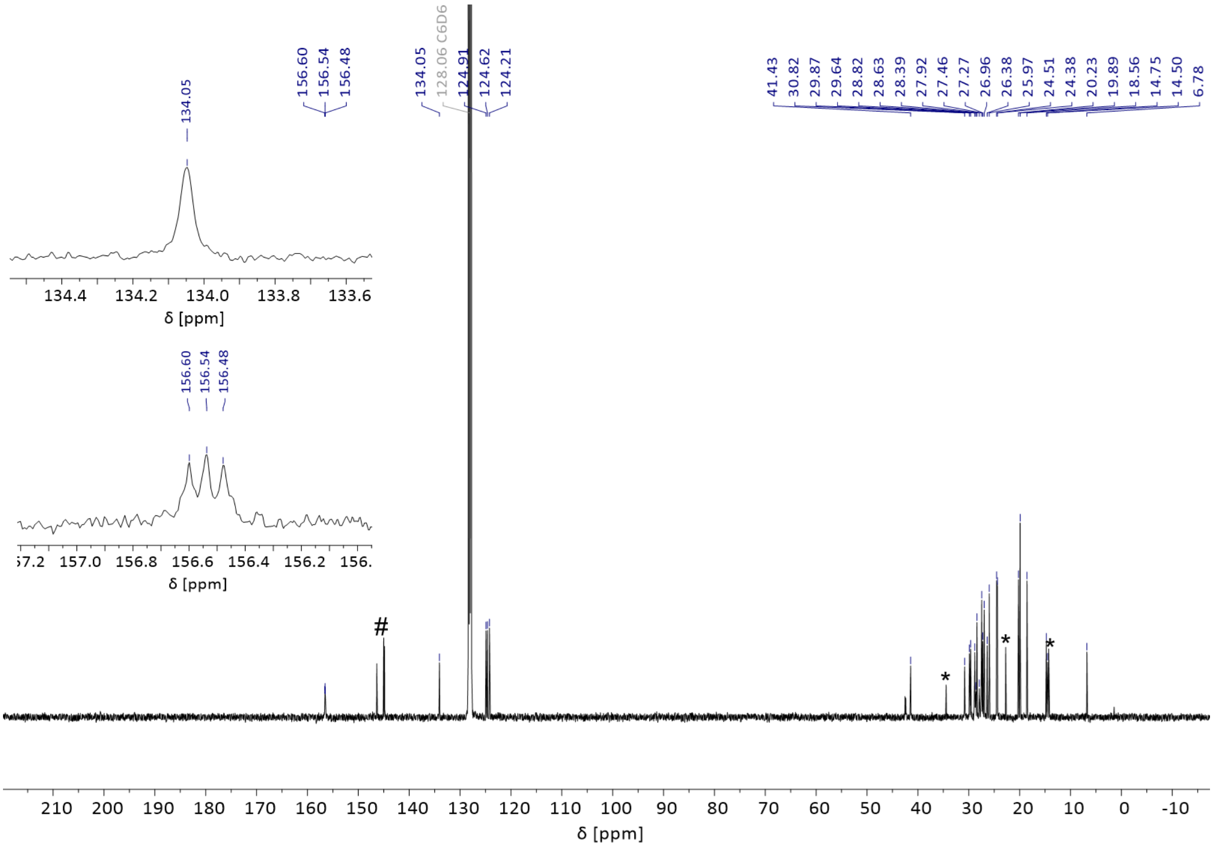

Figure S67. $^{13}C\{^1H\}$ NMR spectrum of **9** as a solution in C_6D_6 at ambient temperature; * indicates small amounts of pentane, while # marks an unknown impurity.

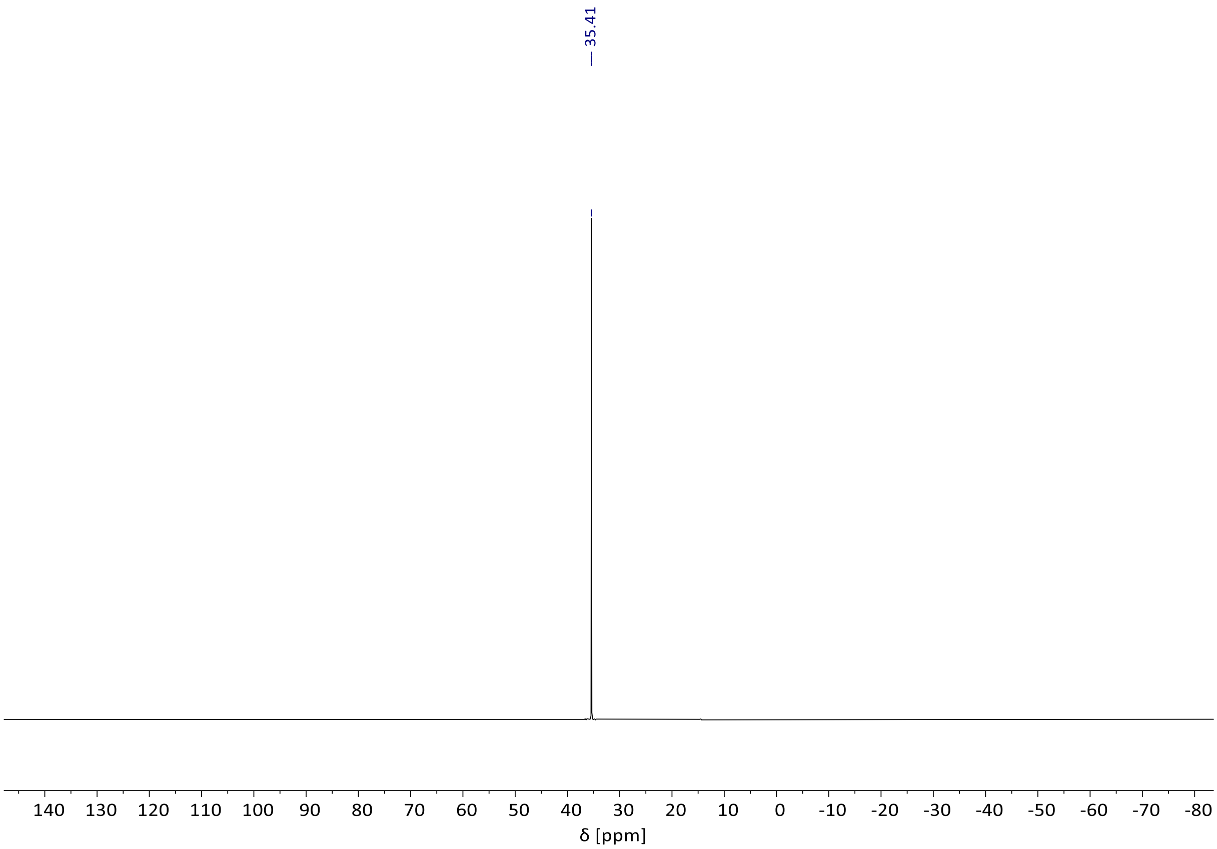

Figure S68. $^{31}P\{^1H\}$ NMR spectrum of **9** as a solution in C_6D_6 at ambient temperature.

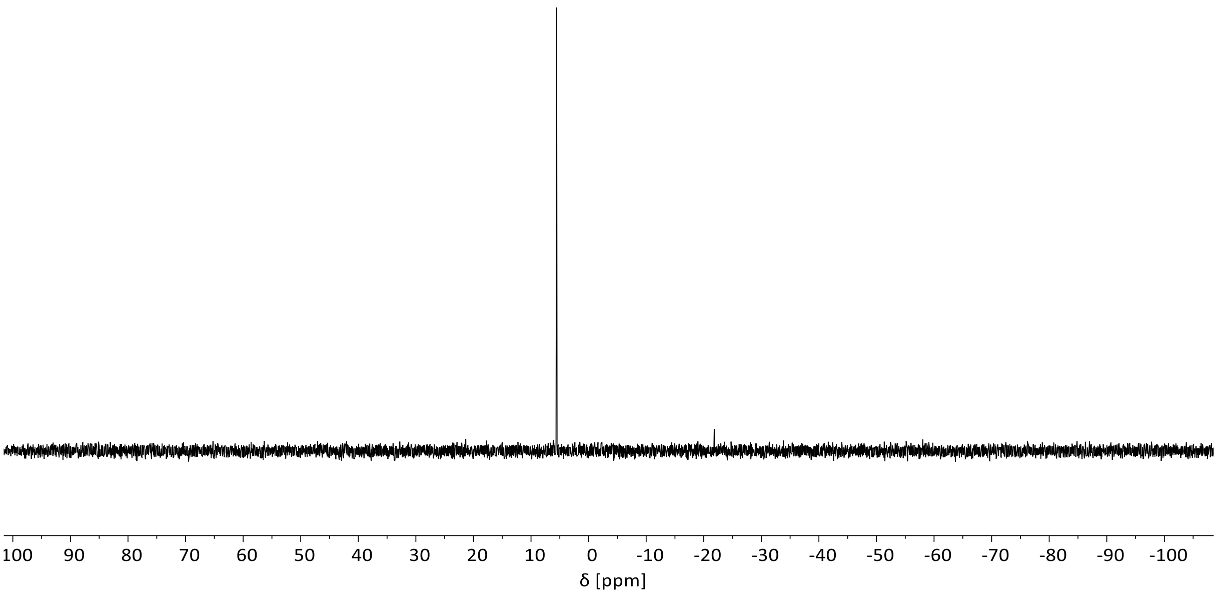

Figure S69. $^{29}Si\{^1H\}$ NMR spectrum of **9** as a solution in C_6D_6 at ambient temperature.

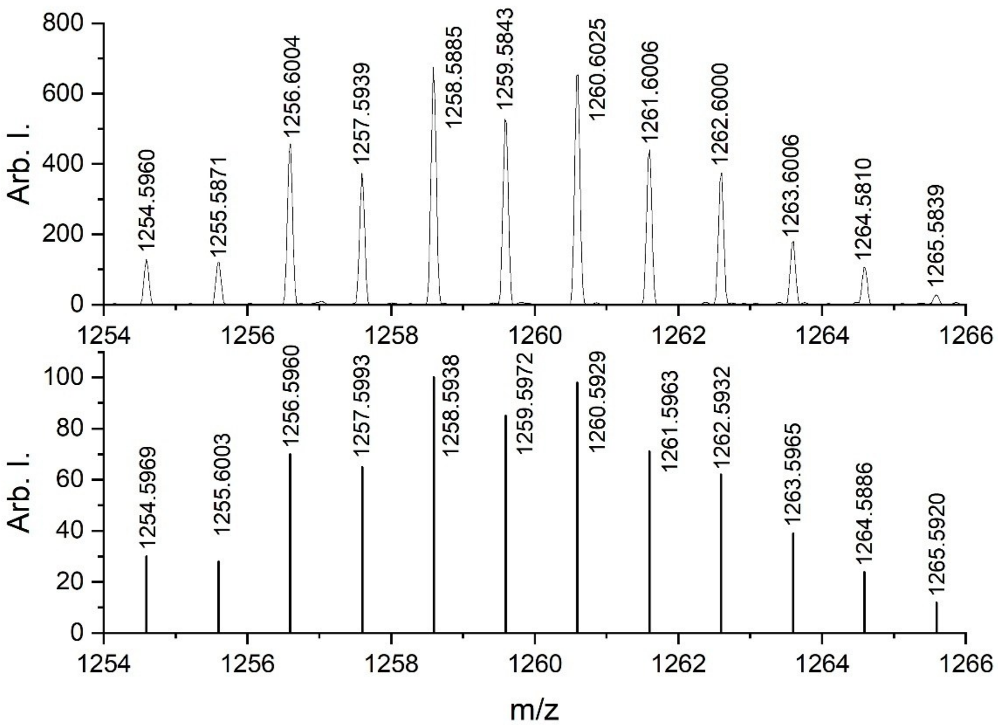

Figure S70. Cutout from LIFDI/MS of **9**; **Top**: found MS for [M]; **Bottom**: Calculated MS spectrum of [M].

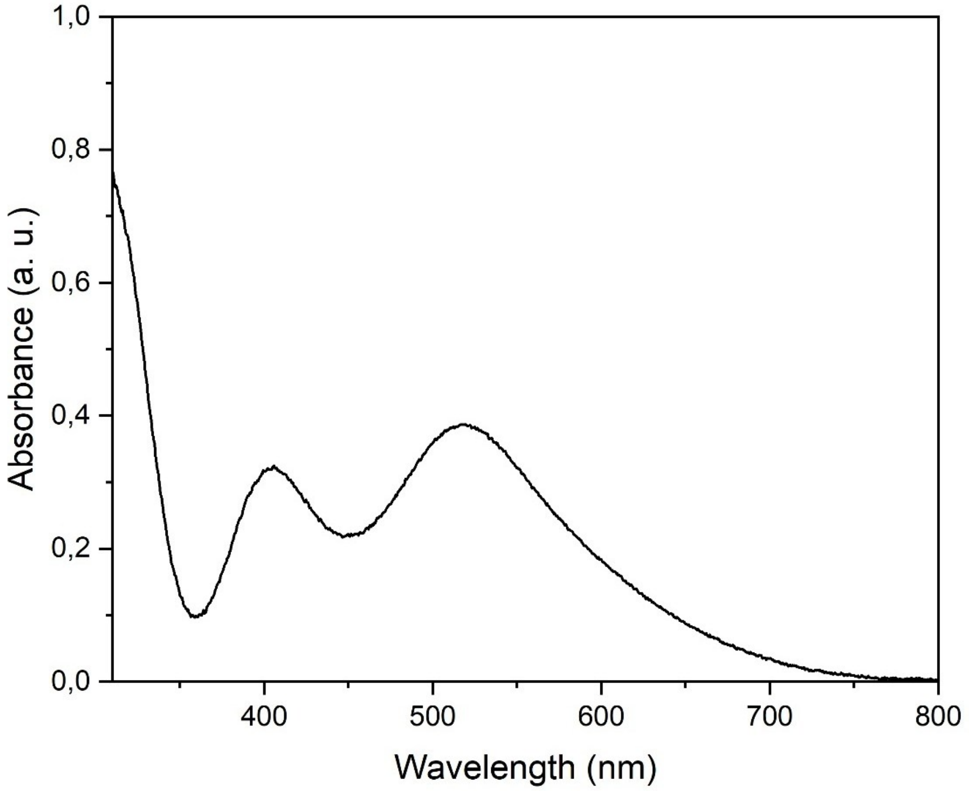

Figure S71. UV/Vis spectrum of a $5x10^{-5}$ M solution of **9** in toluene at ambient temperature.

2. X-ray Crystallographic Details

Single crystals of **4a**, **5b**, **6b**, **7**, **8**, and **9** suitable for X-ray structural analysis were mounted in perfluoroalkyl ether oil on a nylon loop and positioned in a 150 K cold N_2 gas stream. Data collection was performed with a STOE StadiVari diffractometer (MoKα radiation) equipped with a DECTRIS PILATUS 300K detector. Structures were solved using SHELXT-16,[6] and refined by full-matrix least-squares calculations against F_2 (SHELXL-2018).[7] The positions of the hydrogen atoms were calculated and refined using a riding model. All non-hydrogen atoms were treated with anisotropic displacement parameters. Crystal data, details of data collections, and refinements for all structures can be found in their CIF files, which are available free of charge *via* www.ccdc.cam.ac.uk/data_request/cif. Details of crystallographic details are summarized in Tables S1 and S2.

Table S1. Crystallographic details for compound **3a**, **4a**, **5b**, and **6b**.

	**3a**	**4a**	**5b**	**6b**
empirical form.	$C_{31}H_{43}BrNPPbSi$	$C_{33}H_{46}GeNPSi$	$C_{33}H_{58}NPSiSn$	$C_{33}H_{58}NPPbSi$
formula wt	775.82	588.36	646.55	738.82
crystal syst.	monoclinic	monoclinic	monoclinic	triclinic
space group	$P2_1/c$	$P2_1$	$P2_1$	*P-1*
a (Å)	17.600(4)	14.664(3)	15.200(3)	11.099(2)
b (Å)	11.440(2)	11.879(2)	12.070(2)	11.787(2)
c (Å)	17.720(4)	18.395(4)	18.856(4)	14.516(3)
α (deg.)	90	90	90	112.01(3)
β (δεγ)	112.70(3)	90.49(3)	90.70(3)	95.00(3)
γ (deg.)	90	90	90	100.93(3)
vol (Å^3)	3291.4(13)	3204.1(11)	3459.2(12)	1702.4(7)
Z	4	4	4	2
ρ(calc) (g.cm^{-3})	1.566	1.220	1.241	1.441
μ (mm^{-1})	6.444	1.065	0.840	5.140
F(000)	1528	1248	1368	751
T (K)	150(2)	150(2)	150(2)	150(2)
reflns collect.	16630	40912	51050	30438
unique reflns	6403	12355	12824	6666
R_{int}	0.0502	0.1102	0.0872	0.0293
R1 [$I>2\sigma(I)$]	0.0500	0.0658	0.0448	0.0214
*w*R2 (all data)	0.1289	0.1575	0.1073	0.0478
CCDC No.	2489031	2488940	2488941	2488942

Table S2. Crystallographic details for compound **7**, **8**, and **9**.

	**7**	**8**	**9**
empirical form.	$C_{63}H_{55}BF_{24}NPPbSi$	$C_{69}H_{76}GeNNiP_3Si$	$C_{66}H_{116}Ge_2N_2Ni$, 1.5($C_4H_{10}O$)
formula wt	1559.14	1171.60	1364.98
crystal syst.	triclinic	triclinic	monoclinic
space group	*P-1*	*P-1*	*C2/c*
a (Å)	13.664(3)	14.167(3)	44.108(9)
b (Å)	15.947(3)	20.711(4)	17.792(4)
c (Å)	17.014(3)	24.342(5)	20.664(4)
α (deg.)	71.03(3)	66.46(3)	90
β (δεγ)	68.89(3)	83.90(3)	106.58(3)
γ (deg.)	80.34(3)	89.87(3)	90
vol ($Å^3$)	3265.2(14)	6505(3)	15543(6)
Z	2	2	8
ρ(calc) ($g.cm^{-3}$)	1.586	1.196	1.167
μ (mm^{-1})	2.734	0.882	1.120
F(000)	1544	2464	5885
T (K)	150(2)	150(2)	150(2)
reflns collect.	45370	77037	107888
unique reflns	12818	20048	15286
R_{int}	0.0970	0.1360	0.1494
R1 [$I>2\sigma(I)$]	0.0586	0.0898	0.0670
*w*R2 (all data)	0.1355	0.2547	0.1891
CCDC No.	2488943	2488944	2488945

<u>Response to B-level alerts:</u>

Compound 7:

PLAT213

PROBLEM: Atom F20 has ADP max/min Ratio ..... 4.2 prolat

RESPONSE: This is due to disorder in the BArF anion, which has been modelled.

PLAT220

PROBLEM: NonSolvent Resd 1 C Ueq(max)/Ueq(min) Range 7.3 Ratio

RESPONSE: This is due to disorder in the BArF anion, which has been modelled.

Compound 8:

PLAT910

PROBLEM: Missing FCF Reflection(s) Below Theta(Min)[Deg]= 2.14 Note

RESPONSE: Given the overall quality and completeness of the data (R1 = 8.98%; completeness = 99.9%), this does not affect the accuracy of the structure.

Compound 9:

PLAT351

PROBLEM: Long C-H (X0.96,N1.08A) C66 - H66B . 1.17 Ang.

RESPONSE: This relates to one vinylic C-H proton, which was located and freely refined.

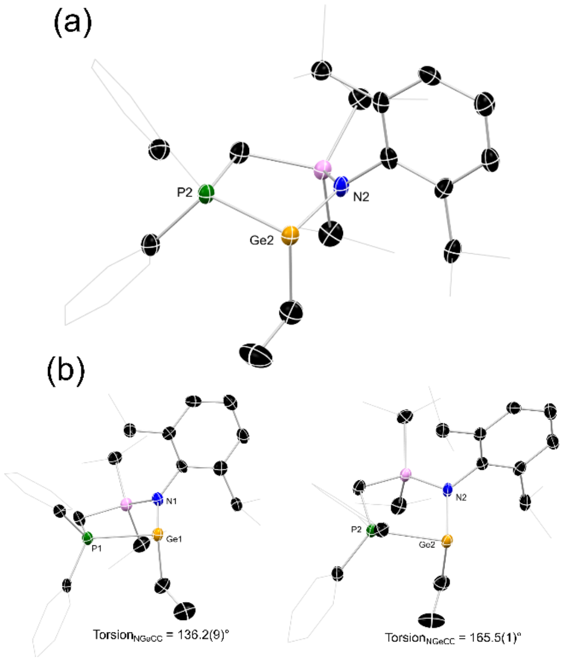

Figure S72. (a) The second molecule in the ASU for **4a**; (b) a comparison of the torsion angles in the two independent molecules of **4a**.

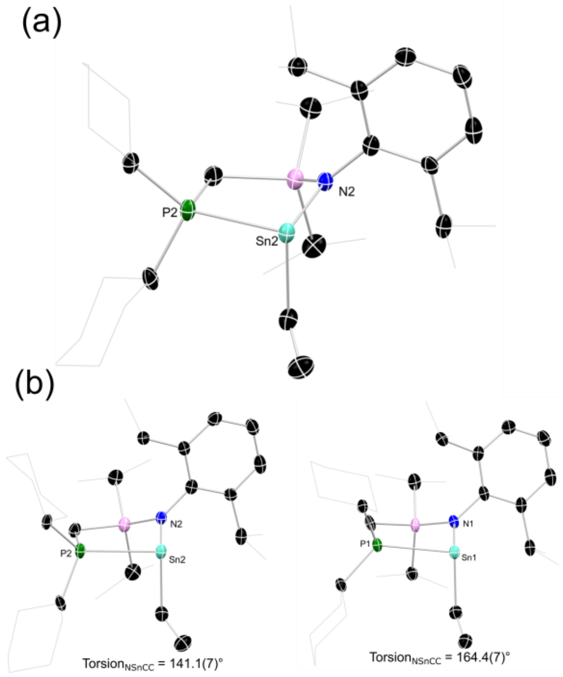

Figure S73. (a) The second molecule in the ASU for **4a**; (b) a comparison of the torsion angles in the two independent molecules of **4a**.

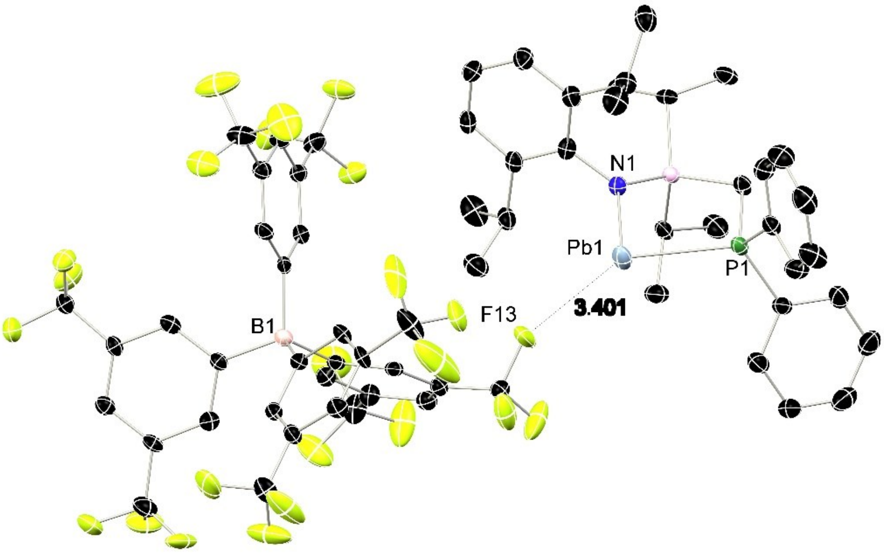

Figure S74. (a) The second molecule in the ASU for **4a**; (b) a comparison of the torsion angles in the two

3. Computational methods and details

Computational experiments were performed using the ORCA 6.0.1 program.[8] Geometry optimization was carried out at the ωB97XD level with the def2-TZVPP basis set for Ge, Ni, and P, and at the def2-SVP basis set for all atoms.[9,10,11,12] Stationary points were confirmed as true minima by vibrational frequency analysis (no negative eigenvalues). Bond indices (Wiberg Bond Index, Mayer Bond Order) and NPA charges were determined using the NBO 7.0 program implemented in ORCA 6.0.1, using optimized geometries from above.[13] Dative interactions were determined through analysis of the NBO output, and visualized in ChemCraft through combination of the associated MOs.

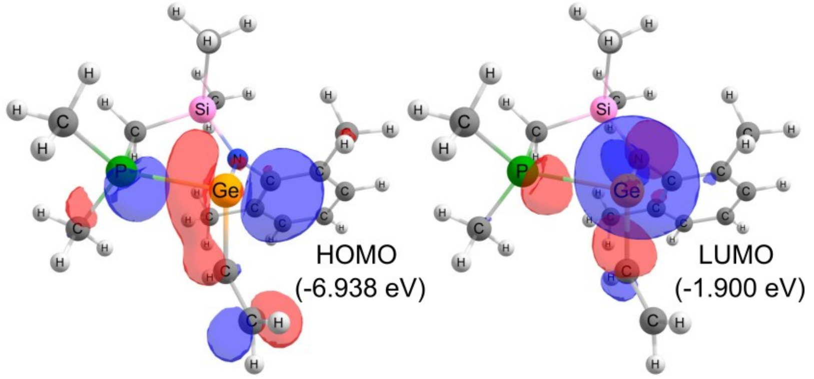

Figure S75. Calculated frontier orbitals for a reduced model of **4a** (*viz*. **4'**).

Table S3. Key orbitals derived from an NBO analysis of germylene-nickel complexes.

		Occupancy	Atom	Orbital Character (%)		
				s	*p*	*d*
**L'(NH_2)Ge·Ni**	Lone Pair	1.75264	Ge	80.60	19.29	0.10
	Lone Pair	1.95153	Ni	0.00	0.00	99.99
	Lone Pair	1.94462	Ni	0.04	0.01	99.95
	Lone Pair	1.94438	Ni	0.01	0.01	99.99
	Lone Pair	1.92052	Ni	0.00	0.01	99.99
	Lone Pair	1.84076	Ni	0.01	0.02	99.98
	Vacant orbital	0.46817	Ge	2.28	97.57	0.08
	Vacant orbital	0.42272	Ni	99.89	0.02	0.09
**L'(Cl)Ge·Ni**	Lone Pair	1.80957	Ge	86.87	13.03	0.08
	Lone Pair	1.95733	Ni	0.08	0.01	99.91
	Lone Pair	1.95325	Ni	0.00	0.01	99.99
	Lone Pair	1.95082	Ni	0.00	0.01	99.99

	Lone Pair	1.91935	Ni	0.00	0.00	99.99
	Lone Pair	1.79666	Ni	0.01	0.03	99.97
	Vacant orbital	0.51781	Ge	3.47	96.38	0.08
	Vacant orbital	0.42221	Ni	99.85	0.02	0.13
**L'(C_2H_3)Ge·Ni**	Lone Pair	1.75099	Ge	78.71	21.18	0.09
	Lone Pair	1.95455	Ni	0.00	0.00	99.99
	Lone Pair	1.94733	Ni	0.04	0.01	99.95
	Lone Pair	1.94592	Ni	0.01	0.01	99.99
	Lone Pair	1.92142	Ni	0.00	0.01	99.99
	Lone Pair	1.80542	Ni	0.00	0.01	99.98
	Vacant orbital	0.45549	Ge	0.45	99.35	0.15
	Vacant orbital	0.42047	Ni	99.89	0.02	0.08

Table S4. NBO derived parameters for bonding in germylene-nickel complexes.

	**L'(NH_2)Ge·Ni**	**L'(Cl)Ge·Ni**	**L'(C_2H_3)Ge·Ni**
**Bond Length**	2.1449	2.1762	2.1712
**NPA Charge**	Ge: 0.85050<br>Ni: -0.04495	Ge: 1.00335<br>Ni: -0.06648	Ge: 0.88891<br>Ni: -0.03696
**MBO**	1.0363	1.0825	1.1356
**WBI**	0.5006	0.4409	0.4972

Table S5. Dative interactions between Ge and Ni with a value >2 kcal·mol^{-1}, from a Second Order Perturbation Theory analysis. Values given in kcal·mol^{-1}.

	**Ge to Ni**	**Ni to Ge**
**L'(NH_2)Ge·Ni**	119.04	3.29<br>3.32<br>20.91
	Sum:	**146.56**
**L'(Cl)Ge·Ni**	102.33	2.03<br>4.59<br>29.6
	Sum:	**138.55**
**L'(C_2H_3)Ge·Ni**	121.3<br>3.96<br>2.27	2.62<br>2.3<br>26.21
	Sum:	**158.66**

Table S6. Cartesian geometry of **4'**.

Atom	x-coordinate	y-coordinate	z-coordinate
Ge	1.30556	0.62437	-0.59694
P	-0.23849	0.86714	1.29498
Si	1.52853	-1.50917	1.81437
N	2.27653	-0.66315	0.48901
C	0.39351	-2.93925	1.34543
C	0.05134	-0.59098	-1.58128
C	4.54004	0.21944	0.86566
C	3.67442	-0.71962	0.25631
C	-1.94528	0.33781	1.01182
C	-0.37008	2.56147	1.91572
C	0.43348	-0.16505	2.62069
H	-0.37037	-0.54736	3.26815
H	1.09085	0.47429	3.23126
C	4.21136	-1.70392	-0.60466
C	5.59117	-1.74061	-0.83362
H	5.99854	-2.50513	-1.50254
C	3.99102	1.27926	1.78525
C	2.82183	-2.16185	3.00945
C	6.44447	-0.81992	-0.23104
H	7.52047	-0.85937	-0.42092
C	5.91459	0.15316	0.61294
H	6.57823	0.88171	1.08911
C	-0.53701	-0.19543	-2.71712
C	3.30573	-2.71093	-1.26442
H	3.86294	-3.32963	-1.98198
H	2.47597	-2.21897	-1.79405
H	2.84714	-3.38448	-0.52318
H	3.5408	0.83811	2.68864
H	3.20085	1.86594	1.29073
H	4.78277	1.9698	2.10721
H	3.4452	-2.91981	2.50788
H	2.34376	-2.6401	3.8788
H	3.48868	-1.36557	3.37312
H	0.94624	-3.74018	0.83017
H	-0.43107	-2.6243	0.68827
H	-0.05108	-3.36908	2.25843
H	-1.96151	-0.73206	0.76542
H	-2.36037	0.89524	0.16061
H	-2.5583	0.51744	1.90658
H	0.63694	2.94301	2.13451
H	-0.98147	2.59502	2.8288
H	-0.82759	3.19567	1.14331
H	-0.1421	-1.61987	-1.24447
H	-0.37482	0.81162	-3.12636
H	-1.2044	-0.84294	-3.30058

Table S7. Cartesian geometry of **8'**.

Atom	x-coordinate	y-coordinate	z-coordinate
Ge	7.343895	13.061338	7.554842
Ni	8.833227	12.932821	5.980365
P	10.440996	11.482823	6.077526
P	7.643613	12.628326	4.182504
P	9.733355	14.882717	5.935341
Si	4.815241	12.74066	5.71019
N	5.487817	13.260283	7.262352
C	7.441027	13.600671	9.456478
C	11.201751	15.045228	4.830957
C	10.414158	15.618776	7.479812
C	8.730491	16.301404	5.319308
C	5.924556	13.289949	4.272181
H	5.38079	13.092354	3.329559
H	6.025766	14.386336	4.361401
C	9.968996	9.752505	6.498484
C	11.807144	11.749289	7.285992
C	4.63453	13.88576	8.212241
C	7.369708	10.906155	3.581006
C	3.878139	13.118192	9.124783
C	5.419356	16.131499	7.348898
C	8.218008	13.378652	2.595266
C	4.558703	15.29672	8.257793
C	3.981846	11.616418	9.142346
C	8.633656	13.541754	10.063649
C	3.036001	13.769018	10.034481
H	2.451116	13.168371	10.737757
C	2.936375	15.157008	10.059863
H	2.270919	15.650156	10.773342
C	11.41244	11.18546	4.537753
C	4.57775	10.87153	5.631922
C	3.70491	15.912504	9.177359
H	3.647567	17.00502	9.200454
C	3.139738	13.560208	5.471203
H	6.57127	13.923154	10.044661
H	9.530912	13.221258	9.515108
H	8.784119	13.804712	11.118348
H	4.40967	10.543189	4.593566
H	3.705201	10.56326	6.227743
H	5.45822	10.338692	6.023943
H	2.701991	13.244001	4.510659
H	3.220404	14.658479	5.467451
H	2.440253	13.275768	6.273142
H	5.02797	11.287239	9.054889
H	3.435258	11.161363	8.301867
H	3.561173	11.206289	10.071039

H	5.275366	15.872001	6.288773
H	6.487364	15.96872	7.569715
H	5.208015	17.202111	7.477499
H	6.635655	10.858396	2.760561
H	7.033279	10.263969	4.405848
H	8.33059	10.50945	3.218837
H	10.895865	16.593245	7.30186
H	11.150429	14.92648	7.914825
H	9.600328	15.743171	8.209712
H	12.303452	12.70864	7.074868
H	12.557321	10.94321	7.253775
H	11.382078	11.806727	8.299427
H	7.535004	13.160408	1.758685
H	9.217933	12.985789	2.354588
H	8.302014	14.469386	2.709864
H	10.923005	14.742235	3.810528
H	11.99843	14.371548	5.180182
H	11.59121	16.075285	4.806345
H	10.73065	10.828705	3.750908
H	12.212698	10.442342	4.682174
H	11.856833	12.130247	4.192117
H	9.222595	9.395382	5.773371
H	9.501714	9.743014	7.49429
H	10.833603	9.069443	6.494731
H	8.361791	16.077475	4.307445
H	9.311262	17.236882	5.289405
H	7.857654	16.440811	5.971224

Table S8. Cartesian geometry of [^{MeMe}Xyl(Cl)Ge·Ni·$(PMe_3)_2$].

**Atom**	**x-coordinate**	**y-coordinate**	**z-coordinate**
Ge	7.329361	13.158754	7.479383
Ni	8.840175	12.967133	5.968969
P	10.408171	11.452667	6.030639
P	7.600666	12.677304	4.199718
P	9.807691	14.898203	6.014898
Si	4.795674	12.691981	5.737621
N	5.480336	13.345542	7.243103
Cl	7.464608	14.2865	9.487199
C	11.249088	15.049917	4.880061
C	10.544733	15.4936	7.589203
C	8.833284	16.376747	5.516489
C	5.854013	13.259701	4.269123
H	5.327895	13.008001	3.330467
H	5.901199	14.361532	4.327549
C	9.85551	9.742005	6.41675
C	11.763243	11.664251	7.255859
C	4.617334	14.001451	8.167888

C	7.408309	10.978489	3.51413
C	3.979067	13.269918	9.191082
C	5.167049	16.199222	7.044355
C	8.202316	13.536177	2.684168
C	4.417617	15.394984	8.070077
C	4.22508	11.793017	9.349081
C	3.125136	13.937478	10.076438
H	2.63179	13.367919	10.869902
C	2.90412	15.307675	9.967637
H	2.234302	15.815841	10.666471
C	11.374901	11.167628	4.490681
C	4.723728	10.810557	5.775171
C	3.55565	16.028441	8.969928
H	3.404275	17.109103	8.888766
C	3.05743	13.36095	5.514326
H	4.518216	10.405578	4.771244
H	3.923896	10.466362	6.448643
H	5.672327	10.380018	6.133406
H	2.620552	12.951739	4.589293
H	3.044115	14.459377	5.444605
H	2.412392	13.066074	6.356967
H	5.302703	11.567767	9.366071
H	3.792303	11.217545	8.516867
H	3.781611	11.41939	10.282327
H	5.008046	15.827132	6.021106
H	6.248771	16.139615	7.242271
H	4.872299	17.257077	7.077765
H	6.722216	10.958692	2.652604
H	7.038117	10.29149	4.286613
H	8.395258	10.61737	3.188151
H	11.034536	16.472818	7.472141
H	11.283416	14.758977	7.942508
H	9.750671	15.564166	8.346828
H	12.31324	12.591338	7.034426
H	12.467456	10.817621	7.244105
H	11.326756	11.760983	8.260985
H	7.549932	13.346556	1.817342
H	9.220205	13.186508	2.453794
H	8.248187	14.619432	2.868049
H	10.938169	14.801318	3.854526
H	12.028167	14.335262	5.184258
H	11.674092	16.065717	4.892172
H	10.692153	10.855237	3.686831
H	12.147063	10.394268	4.626309
H	11.856292	12.105591	4.178168
H	9.106909	9.428266	5.674754
H	9.373857	9.738363	7.405526
H	10.691635	9.024944	6.414877
H	8.377071	16.205613	4.530305

H	9.453195	17.285972	5.475061
H	8.021058	16.53205	6.239872

Table S9. Cartesian geometry of [^{MeMe}Xyl(H_2N)Ge·Ni·(PMe_3)$_2$].

Atom	x-coordinate	y-coordinate	z-coordinate
Ge	7.347313	13.174025	7.54052
Ni	8.8278	12.940733	5.962683
P	10.37948	11.440613	6.051263
P	7.624606	12.652456	4.182981
P	9.774814	14.870142	5.969537
Si	4.819271	12.699618	5.732321
N	5.484442	13.323871	7.243589
N	7.391296	13.962095	9.194974
C	11.276821	15.063558	4.916294
C	10.412513	15.524596	7.570234
C	8.803541	16.329387	5.396942
C	5.884474	13.262013	4.266092
H	5.345769	13.033454	3.327793
H	5.95072	14.362235	4.340471
C	9.875154	9.702864	6.402748
C	11.731475	11.64198	7.28851
C	4.636173	13.959379	8.188388
C	7.392521	10.949241	3.510411
C	3.980188	13.208537	9.189294
C	5.23225	16.187989	7.148683
C	8.19938	13.472524	2.630444
C	4.476543	15.364478	8.154988
C	4.169146	11.717372	9.277916
C	3.153935	13.86498	10.109612
H	2.647984	13.27785	10.882198
C	2.974177	15.24436	10.060551
H	2.325323	15.7429	10.785594
C	11.37659	11.182216	4.520456
C	4.705725	10.817199	5.73937
C	3.641262	15.985742	9.088019
H	3.520735	17.072816	9.051619
C	3.089206	13.396594	5.496598
H	4.505118	10.429729	4.727516
H	3.891707	10.479257	6.398884
H	5.642116	10.365522	6.103505
H	2.650622	13.003917	4.56515
H	3.096764	14.496009	5.435224
H	2.429975	13.108624	6.330911
H	5.231966	11.443954	9.198702
H	3.645535	11.195157	8.462257
H	3.777553	11.328201	10.228079
H	5.009276	15.888449	6.113204

H	6.315555	16.054749	7.293208
H	5.000235	17.256474	7.257439
H	6.682968	10.92633	2.66763
H	7.039328	10.269955	4.297585
H	8.367347	10.576529	3.160428
H	10.885524	16.514015	7.467352
H	11.144374	14.814249	7.983287
H	9.571648	15.589717	8.277031
H	12.262192	12.586779	7.095278
H	12.45554	10.812091	7.260359
H	11.289876	11.704171	8.29445
H	7.529567	13.274266	1.778367
H	9.209272	13.108104	2.38657
H	8.262161	14.559084	2.788964
H	11.02519	14.810417	3.875271
H	12.053051	14.362481	5.258195
H	11.681827	16.087389	4.949036
H	10.707801	10.874738	3.702542
H	12.15765	10.416394	4.651354
H	11.849496	12.130855	4.227001
H	9.141322	9.381402	5.648869
H	9.384371	9.665444	7.386695
H	10.729746	9.007305	6.395441
H	8.434726	16.148714	4.376451
H	9.395575	17.258282	5.405176
H	7.929166	16.455272	6.050188
H	6.578577	14.194066	9.758483
H	8.241046	13.905891	9.745639

4. References

(1) Keil, P. M.; Szilvási, T.; Hadlington, T. J. Reversible Metathesis of Ammonia in an Acyclic Germylene–Ni^0 Complex. *Chemical Science* **2021**, *12* (15), 5582–5590. https://doi.org/10.1039/D1SC00450F.

(2) Kalkuhl, T. L.; Fernández, I.; Hadlington, T. J. Cooperative Hydrogenation Catalysis at a Constrained Gallylene-Nickel(0) Interface. *Chem* **2025**, *11* (4), 102349. https://doi.org/10.1016/j.chempr.2024.10.016.

(3) Keil, P. M.; Hadlington, T. J. Geometrically Constrained Cationic Low-Coordinate Tetrylenes: Highly Lewis Acidic σ-Donor Ligands in Catalytic Systems. *Angew Chem Int Ed* **2022**, *61* (8), e202114143. https://doi.org/10.1002/anie.202114143.

(4) Sicard, A. J.; Baker, R. T. Safe and Expeditious Preparation of Ni(Cod)2 for Same-Day High-Throughput Screening. *Org. Process Res. Dev.* **2020**, *24* (12), 2950–2952. https://doi.org/10.1021/acs.oprd.0c00435.

(5) Maciejewski, H.; Sydor, A.; Marciniec, B.; Kubicki, M.; Hitchcock, P. B. Intermediates in Nickel(0)–Phosphine Complex Catalyzed Dehydrogenative Silylation of Olefins. *Inorganica Chimica Acta* **2006**, *359* (9), 2989–2997. https://doi.org/10.1016/j.ica.2005.12.067.

(6) Sheldrick, G. M. *SHELXT* – Integrated Space-Group and Crystal-Structure Determination. *Acta Crystallogr A Found Adv* **2015**, *71* (1), 3–8. https://doi.org/10.1107/S2053273314026370.

(7) Sheldrick, G. M. Crystal Structure Refinement with *SHELXL*. *Acta Crystallogr C Struct Chem* **2015**, *71* (1), 3–8. https://doi.org/10.1107/S2053229614024218.

(8) Neese, F. The ORCA Program System. *WIREs Comput Mol Sci* **2012**, *2* (1), 73–78. https://doi.org/10.1002/wcms.81.

(9) Becke, A. D. Density-Functional Thermochemistry. V. Systematic Optimization of Exchange-Correlation Functionals. *The Journal of Chemical Physics* **1997**, *107* (20), 8554–8560. https://doi.org/10.1063/1.475007.

(10) Weigend, F.; Ahlrichs, R. Balanced Basis Sets of Split Valence, Triple Zeta Valence and Quadruple Zeta Valence Quality for H to Rn: Design and Assessment of Accuracy. *Phys. Chem. Chem. Phys.* **2005**, *7* (18), 3297. https://doi.org/10.1039/b508541a.

(11) Chai, J.-D.; Head-Gordon, M. Systematic Optimization of Long-Range Corrected Hybrid Density Functionals. *The Journal of Chemical Physics* **2008**, *128* (8), 084106. https://doi.org/10.1063/1.2834918.

(12) Grimme, S.; Antony, J.; Ehrlich, S.; Krieg, H. A Consistent and Accurate Ab Initio Parametrization of Density Functional Dispersion Correction (DFT-D) for the 94 Elements H-Pu. *The Journal of Chemical Physics* **2010**, *132* (15), 154104. https://doi.org/10.1063/1.3382344.

(13) Glendening, E. D.; Badenhoop, J. K.; Reed, A. E.; Carpenter, J. E.; Bohmann, J. A.; Moralis, C. M.; Landin, C. R.; Weinhold, F. NBO 7.0, 2013.
